# Supplementary material for: The ERBB-STAT3 Axis Drives Tasmanian Devil Facial Tumor Disease
Source: Cancer Cell. 2019 Jan 14;35(1):125–139.e9. doi: 10.1016/j.ccell.2018.11.018 (PMC6335503; doi:10.1016/j.ccell.2018.11.018)
Supplement: Document S1. Figures S1–S6 [file mmc1.pdf]

Cancer Cell, Volume *35*

Supplemental Information

The ERBB-STAT3 Axis Drives Tasmanian Devil Facial Tumor Disease

Lindsay Kosack, Bettina Wingelhofer, Alexandra Popa, Anna Orlova, Benedikt Agerer, Bojan Vilagos, Peter Majek, Katja Parapatics, Alexander Lercher, Anna Ringler, Johanna Klughammer, Mark Smyth, Kseniya Khamina, Hatoon Baazim, Elvin D. de Araujo, David A. Rosa, Jisung Park, Gary Tin, Siawash Ahmar, Patrick T. Gunning, Christoph Bock, Hannah V. Siddle, Gregory M. Woods, Stefan Kubicek, Elizabeth P. Murchison, Keiryn L. Bennett, Richard Moriggl, and Andreas Bergthaler

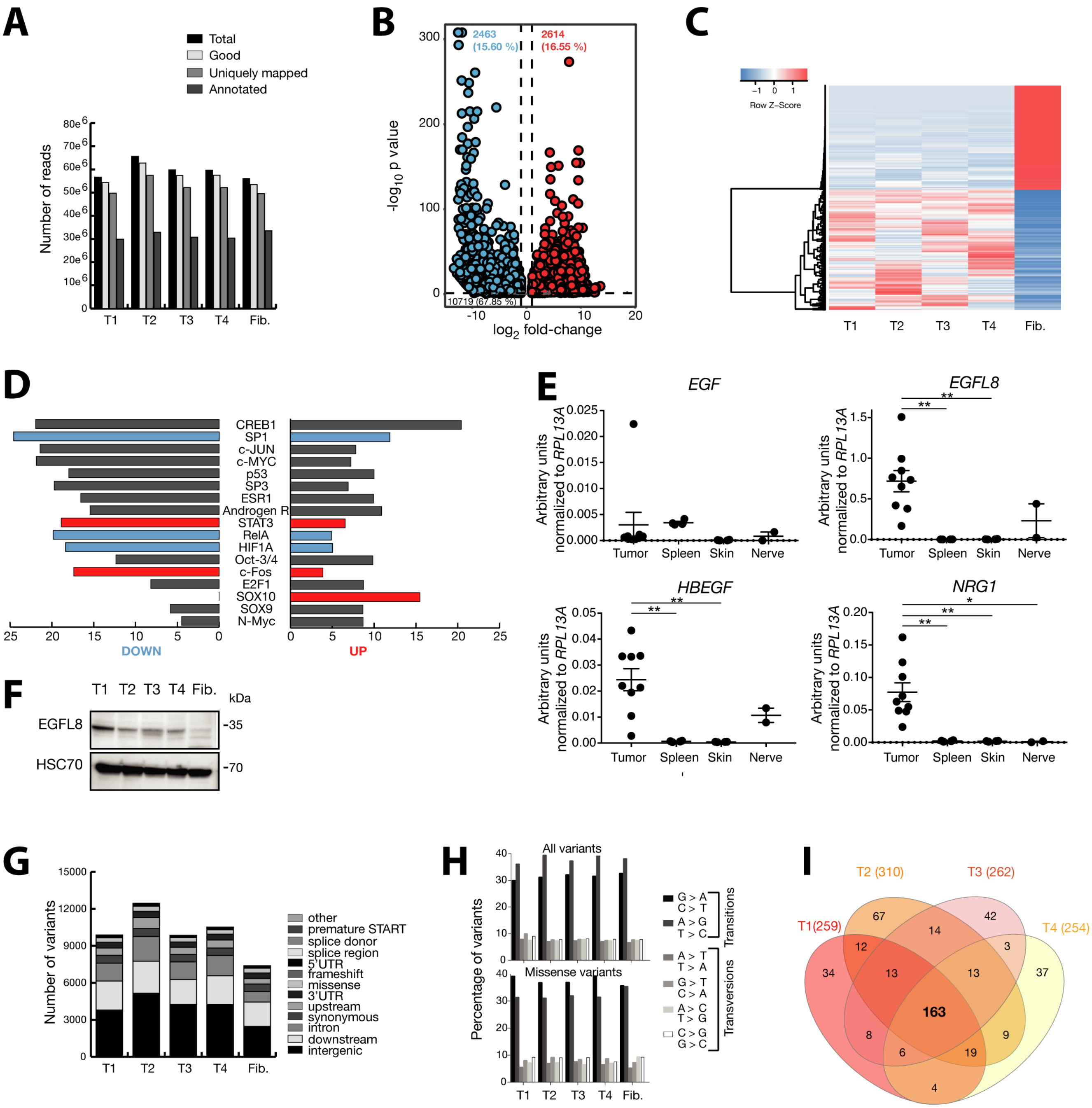

A
Total
Good
Uniquely mapped
Annotated
Number of reads
T1
T2
T3
T4
Fib.
B
2463
(15.60 %)
2614
(16.55 %)
10719 (67.85 %)
-log10 p value
log2 fold-change
C
Row Z-Score
T1
T2
T3
T4
Fib.
D
CREB1
SP1
c-JUN
c-MYC
p53
SP3
ESR1
Androgen R
STAT3
RelA
HIF1A
Oct-3/4
c-Fos
E2F1
SOX10
SOX9
N-Myc
DOWN
UP
E
EGF
EGFL8
HBEGF
NRG1
Arbitrary units normalized to RPL13A
Tumor
Spleen
Skin
Nerve
F
T1
T2
T3
T4
Fib.
kDa
EGFL8
HSC70
-35
-70
G
Number of variants
other
premature START
splice donor
splice region
5'UTR
frameshift
missense
3'UTR
upstream
synonymous
intron
downstream
intergenic
H
All variants
Missense variants
Percentage of variants
G > A
C > T
A > G
T > C
Transitions
A > T
T > A
G > T
C > A
A > C
T > G
C > G
G > C
Transversions
I
T1(259)
T2 (310)
T3 (262)
T4 (254)

Figure S1, Related to Figure 1: RNA-seq and expression analysis

(**A**) Number of reads across four DFTD cell lines (T1-T4) and fibroblasts (Fib.). Total number of reads, reads passing the quality filters (good reads), reads mapping at unique positions on the genome and reads mapping on annotated genes are reported. (**B**) Volcano plot of the differentially expressed genes between the four DFTD and the fibroblast cell lines. 2463 genes are down-regulated (blue), and 2614 genes are up-regulated (red). (**C**) Heatmap of the 5077 differentially expressed genes between the DFTD and the fibroblast cell lines (expression ≥ 50 read counts, absolute log2 fold-change ≥ 1, and adjusted p value ≤ 0.05). (**D**) MetaCore Transcription Factor enrichment analysis. Enrichments analysis has been performed separately on the genes UP and DOWN modulated in the DFTD cells compared to fibroblasts. Transcription factors that are themselves modulated in our dataset are reported in bold and their bars colored in red or blue, for up and respectively down modulated. All transcription factors with a reported bar have significant enrichment p values (≤ 0.05). SOX10 that is highly expressed in the tumor cell lines and enriched exclusively for up-regulated genes is a transcription factor critical for Schwann cell development. Other enriched factors, like STAT3 are more abundant in the DFTD cell lines while, ESR1 is not expressed in the tumor cell lines. (**E**) *EGF, EGFL8, HBEGF* and *NRG1* were measured by real-time PCR from primary biopsies of DFTD tumors, spleen, skin and peripheral nerve. Statistical significance was calculated by One-way ANOVA. Scatter plots are shown with mean +/- SEM. (**F**) Western blot for EGFL8 from DFTD cell lines and Tasmanian devil fibroblasts. (**G**) Annotation classes of variant effects across the five cell lines. The vast majority of variants are localized in intergenic, downstream or intronic regions. (**H**) Transitions and transversions distribution of single nucleotide variants (SNVs) across samples. All the five samples are enriched for transitions across all or only missense SNVs. (**I**) Venn diagram of missense variants shared or specific for the tumor strains and absent from fibroblasts. 38% (163 out of 444 variants) are shared across the four strains.

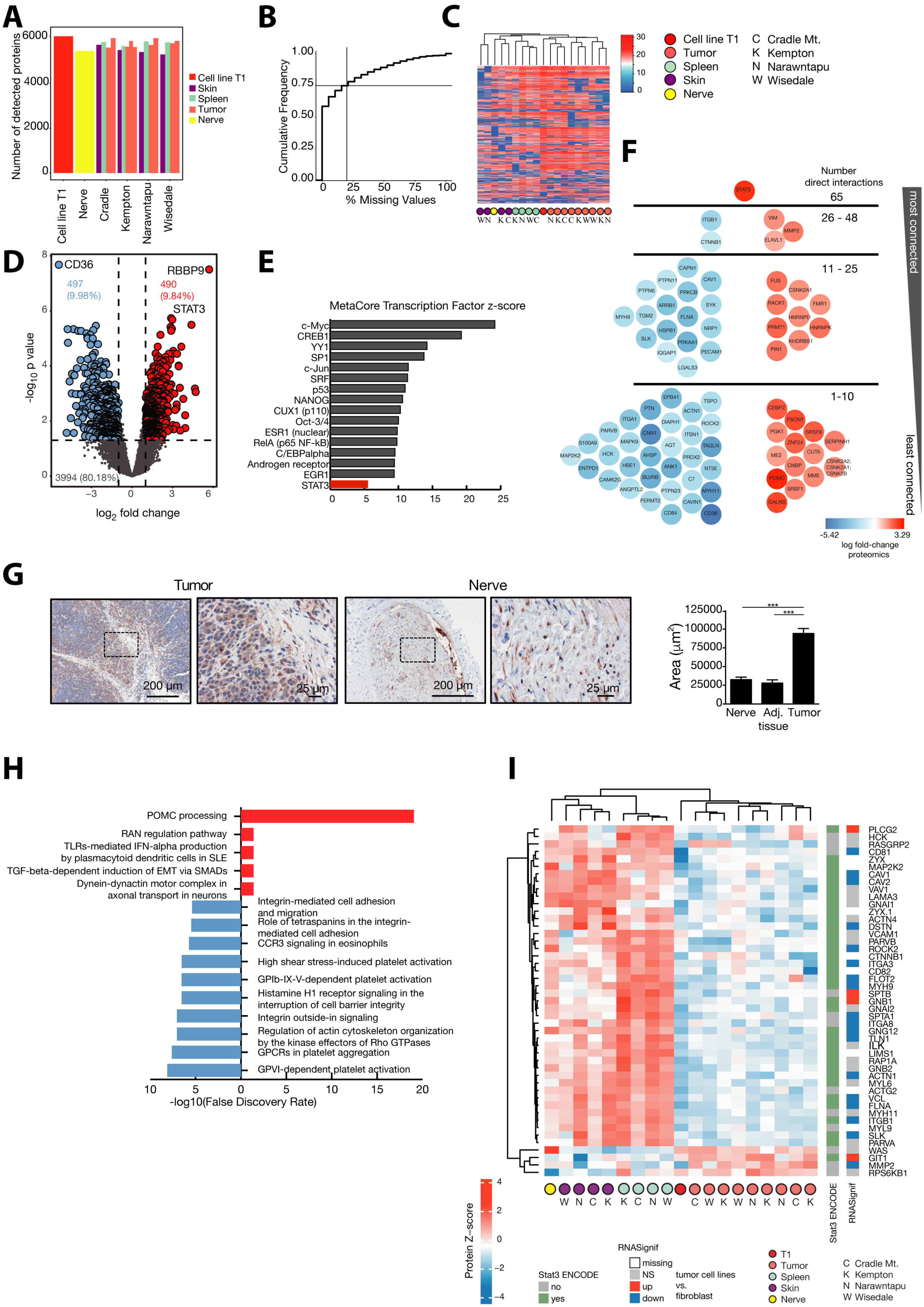

A
Number of detected proteins
6000
4000
2000
0
Cell line T1
Skin
Spleen
Tumor
Nerve
Cell line T1
Nerve
Cradle
Kempton
Narawntapu
Wisedale
B
Cumulative Frequency
1.00
0.75
0.50
0.25
0.00
0
25
50
75
100
% Missing Values
C
30
20
10
0
Cell line T1
Tumor
Spleen
Skin
Nerve
C Cradle Mt.
K Kempton
N Narawntapu
W Wisedale
D
CD36
RBBP9
497
(9.98%)
490
(9.84%)
STAT3
3994 (80.18%)
-log10 p value
log2 fold change
E
MetaCore Transcription Factor z-score
c-Myc
CREB1
YY1
SP1
c-Jun
SRF
p53
NANOG
CUX1 (p110)
Oct-3/4
ESR1 (nuclear)
RelA (p65 NF-kB)
C/EBPalpha
Androgen receptor
EGR1
STAT3
0
5
10
15
20
25
F
Number
direct interactions
65
26 - 48
11 - 25
1-10
most connected
least connected
-5.42
3.29
log fold-change
proteomics
G
Tumor
Nerve
200 μm
25 μm
200 μm
25 μm
125000
100000
75000
50000
25000
0
Area (μm²)
***
***
Nerve
Adj.
tissue
Tumor
H
POMC processing
RAN regulation pathway
TLRs-mediated IFN-alpha production
by plasmacytoid dendritic cells in SLE
TGF-beta-dependent induction of EMT via SMADs
Dynein-dynactin motor complex in
axonal transport in neurons
Integrin-mediated cell adhesion
and migration
Role of tetraspanins in the integrin-
mediated cell adhesion
CCR3 signaling in eosinophils
High shear stress-induced platelet activation
GPIb-IX-V-dependent platelet activation
Histamine H1 receptor signaling in the
interruption of cell barrier integrity
Integrin outside-in signaling
Regulation of actin cytoskeleton organization
by the kinase effectors of Rho GTPases
GPCRs in platelet aggregation
GPVI-dependent platelet activation
10
5
0
5
10
15
20
-log10(False Discovery Rate)
I
PLCG2
HCK
RASGRP2
CD81
ZYX
MAP2K2
CAV1
CAV2
VAV1
LAMA3
GNAI1
ZYX.1
ACTN4
DSTN
VCAM1
PARVB
ROCK2
CTNNB1
ITGA3
CD82
FLOT2
MYH9
SPTB
GNB1
GNAI2
SPTA1
ITGA8
GNG12
TLN1
ILK
LIMS1
RAP1A
GNB2
ACTN1
MYL6
ACTG2
VCL
FLNA
MYH11
ITGB1
MYL9
SLK
PARVA
WAS
GIT1
MMP2
RPS6KB1
Stat3 ENCODE
RNASignif
Protein Z-score
4
2
0
-2
-4
RNASignif
missing
NS
up
down
tumor cell lines
vs.
fibroblast
Stat3 ENCODE
no
yes
T1
Tumor
Spleen
Skin
Nerve
C Cradle Mt.
K Kempton
N Narawntapu
W Wisedale

Figure S2, Related to Figure 2: Proteomic analysis

(**A**) Number of quantified proteins for each sample. Individual Tasmanian devils are named according to their geographical sampling locations. "Cell line" denotes the DFTD cell line 06/2887 (labeled as T1 throughout the study) and "Nerve" stands for a healthy nerve biopsy (**Table S1**). (**B**) Cumulative frequency distribution of the percentage missing (non-quantified) protein abundance across the samples. We focus on proteins quantified in at least 80% of the samples (less than 20% missing values). This represents 4981/6672 proteins, almost 75% of the total identified proteins. (**C**) Hierarchical clustering of missing values (0-blue values) across samples. Missing values are not specific to one sample or condition but are distributed across all samples. (**D**) Volcano plot of the 4981 proteins on which we performed differential analysis. (**E**) Top 15 enriched transcription factors (TF) and STAT3 for the differentially modulated proteins: tumor versus healthy samples. The enrichment was performed with MetaCore™ (Thomson Reuters, version 6.32 build 69020) and the reported TF have all p values ≤ 0.05 and are ordered according to their z-score of enrichment. STAT3 is the most enriched TF that is itself differentially modulated in the proteomics dataset. (**F**) Proteins directly connected to and downstream of STAT3 (based on MetaCore database). Red proteins are more abundant in tumors compared to healthy biopsies, while blue stands for reduced protein in tumors. Proteins are ordered vertically according to how many connections they have inside the STAT3 downstream network, from the most connected (UP) to the least connected (DOWN). (**G**) Immunohistochemical stainings for MMP2 from tumor and peripheral nerve tissue biopsies. Shown images are consecutive images related to **Fig. 1E**. Dotted rectangle indicates magnified area. Quantification of MMP2 in tumor, adjacent tissue and peripheral nerve tissue. Scale bars, 200 μm and 25 μm. Statistical significance was calculated by unpaired t-test. Bars represent mean +/- SEM. (**H**) MetaCore Pathway Maps enrichments separately for UP (red) and DOWN-regulated (blue) proteins in tumor versus healthy tissues. Only the first ten pathways and with a false discovery rate ≤ 0.05 are reported. (**I**) Heatmap of the modulated proteins between tumor biopsies

versus the healthy tissue and involved in chemotaxis and cytoskeleton remodeling (46 proteins). The vast majority of these proteins (42/46) show down-regulation both in the tumor biopsies as well as the DFTD cell line. 34 out of these 46 genes have been shown to be bound by STAT3 across the ENCODE Chip datasets. The modulation status of the transcripts of each gene in the RNA-seq dataset tumor cell lines versus fibroblast is also reported.

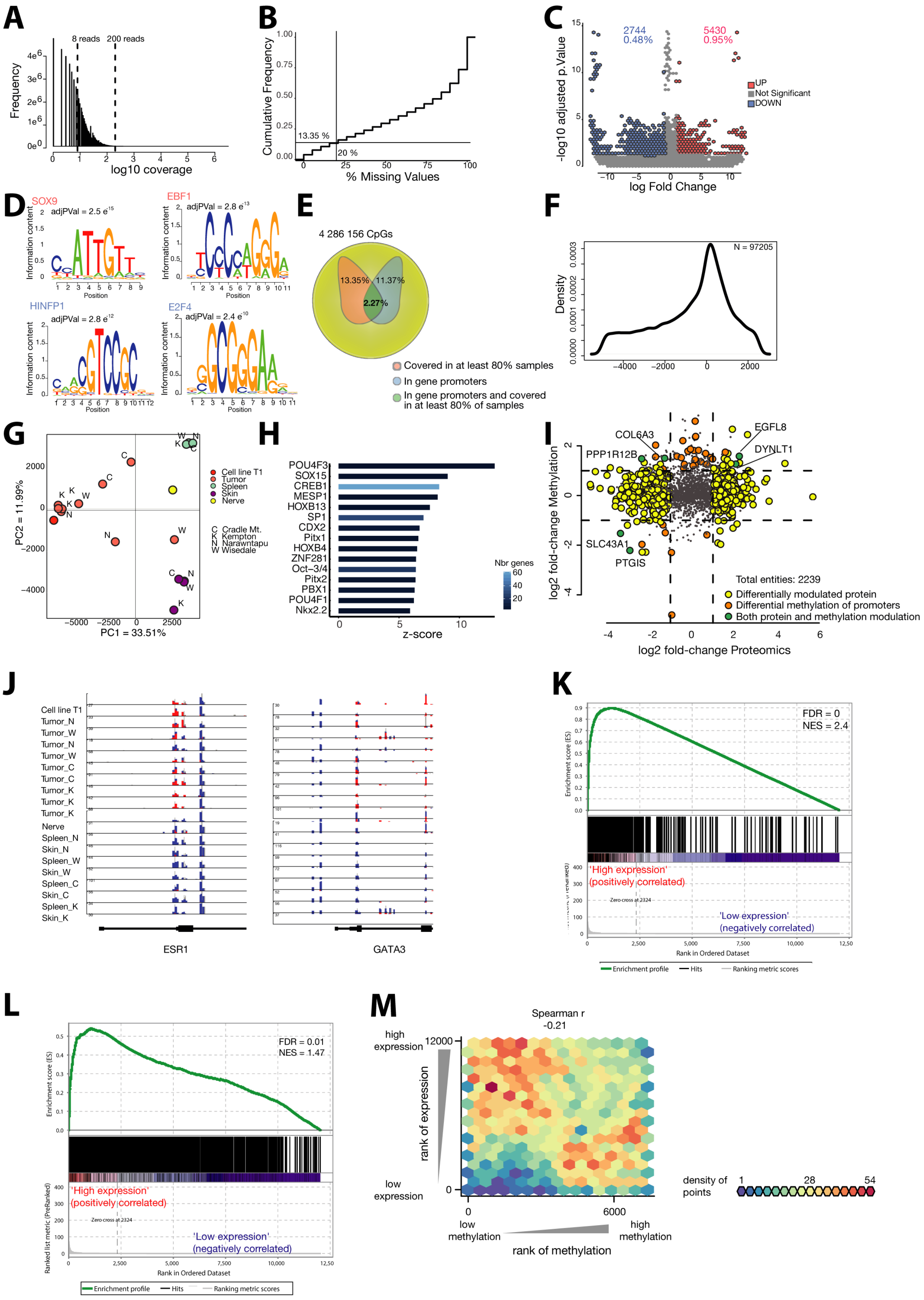
A
8 reads
200 reads
Frequency
log10 coverage
B
Cumulative Frequency
13.35 %
20 %
% Missing Values
C
2744
0.48%
5430
0.95%
-log10 adjusted p.Value
log Fold Change
UP
Not Significant
DOWN
D
SOX9
adjPVal = 2.5 e15
EBF1
adjPVal = 2.8 e13
HINFP1
adjPVal = 2.8 e12
E2F4
adjPVal = 2.4 e10
Information content
Position
E
4 286 156 CpGs
13.35%
11.37%
2.27%
Covered in at least 80% samples
In gene promoters
In gene promoters and covered in at least 80% of samples
F
N = 97205
Density
G
PC2 = 11.99%
PC1 = 33.51%
Cell line T1
Tumor
Spleen
Skin
Nerve
C Cradle Mt.
K Kempton
N Narawntapu
W Wisedale
H
POU4F3
SOX15
CREB1
MESP1
HOXB13
SP1
CDX2
Pitx1
HOXB4
ZNF281
Oct-3/4
Pitx2
PBX1
POU4F1
Nkx2.2
z-score
Nbr genes
I
COL6A3
PPP1R12B
EGFL8
DYNLT1
SLC43A1
PTGIS
Total entities: 2239
Differentially modulated protein
Differential methylation of promoters
Both protein and methylation modulation
log2 fold-change Methylation
log2 fold-change Proteomics
J
Cell line T1
Tumor_N
Tumor_W
Tumor_N
Tumor_W
Tumor_C
Tumor_C
Tumor_K
Tumor_K
Tumor_K
Nerve
Spleen_N
Skin_N
Spleen_W
Skin_W
Spleen_C
Skin_C
Spleen_K
Skin_K
ESR1
GATA3
K
FDR = 0
NES = 2.4
Enrichment score (ES)
'High expression'
(positively correlated)
Zero cross at 2324
'Low expression'
(negatively correlated)
Rank in Ordered Dataset
Enrichment profile
Hits
Ranking metric scores
L
FDR = 0.01
NES = 1.47
Enrichment score (ES)
Ranked list metric (PreRanked)
'High expression'
(positively correlated)
Zero cross at 2324
'Low expression'
(negatively correlated)
Rank in Ordered Dataset
Enrichment profile
Hits
Ranking metric scores
M
Spearman r
-0.21
high expression
low expression
rank of expression
low methylation
high methylation
rank of methylation
density of points

Figure S3, Related to Figure 2: Global DNA methylation analysis and correlations

(**A**) Coverage histogram across the individual CpGs (4,286,156). CpGs covered by at least eight and at most 200 reads are considered for further analysis. Coverage lower than eight reads was deemed insufficient for computing accurate DNA methylation levels. Furthermore, sites covered by more than 200 reads might harbor unreliable measurements introduced by excessive amplification or repetitive sequences. (**B**) Cumulative frequency distribution of the percentage of not detected CpGs across the samples. 572018 (13.35%) of CpGs are detected in at least 80% of samples (15 out of 19). (**C**) Healthy versus Tumor volcano plot of individual detected CpGs in more than 80% of the samples (572018 CpGs). The differential analysis of healthy versus tumor samples (cell line excluded) revealed a total of 2744 tumor hypermethylated and 5430 tumor hypomethylated CpGs (absolute log Fold-change $\geq$1, adjusted p value $\leq$ 0.05). (**D**) AME motif enrichment in fragments hypomethylated or hypermethylated in tumor samples compared to healthy tissues, red and blue respectively. The top two enriched motifs (sorted according to the adjusted p value of enrichment) are shown for each class of CpGs. (**E**) Characterizing individual CpGs: detected, annotated. 97 205 individual CpGs are detected in at least 80% of the samples and localized in the promoters of annotated genes (from -5 kb to 2.5 kb around the TSS). (**F**) Density distribution of the CpG distance to the Transcription Start Site (TSS) of annotated genes. (**G**) Principal component analysis on the promoter averaged CpGs across the 19 samples. Samples separate mainly on the tumor versus healthy profiles (PC1 – 33.5% of the variability), with spleen versus skin differences driving the PC2 (11.99% of the variability). Sampling locations are indicated in capital letters (C Cradle Mountain, K Kempton, N Narawntapu, W Wisedale). (**H**) Top 15 enriched transcription factors (TF) for the differentially methylated gene promoters: healthy versus tumor samples. The enrichment was performed with MetaCore and the reported TF have all p values <= 0.05 and are ordered according to their z-score of enrichment. (**I**) Comparison of the proteomics and methylation hits. Log fold-change of tumor versus healthy analysis was compared to the log fold-change of promoter averaged CpGs healthy versus tumor. 2239 entities were detected in both analyses. Yellow, orange and green circles stand for

entities found as differentially modulated only in proteomics, only in methylation or in both, respectively. (**J**) Integrative Genomics Viewer plots of promoter CpG methylation across samples for *ESR1* and *GATA3*. Red stands for methylated, while blue stands for un-methylated. (**K** and **L**) Gene set enrichment analysis pre-ranked by the RNA-seq expression of the top 15% most abundant proteins (817) (**K**) and the top 15% least methylated (936) gene promoters (**L**) of DFTD cell line (T1). (**M**) Comparison of ranked gene expression and ranked gene promoter methylation of the DFTD cell line (T1) (6974 genes). Spearman correlation is reported.

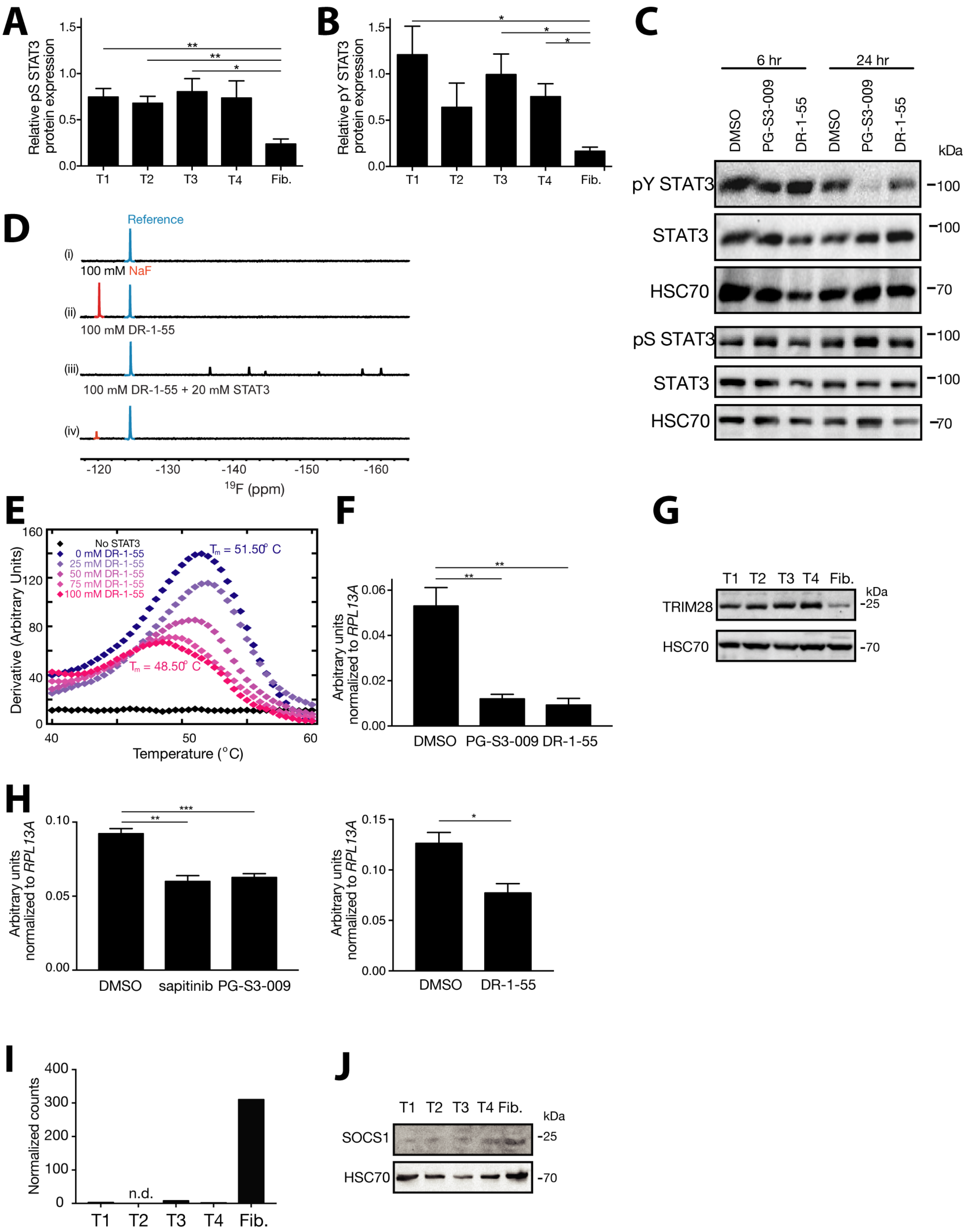

A
Relative pS STAT3 protein expression
T1
T2
T3
T4
Fib.
B
Relative pY STAT3 protein expression
C
6 hr
24 hr
DMSO
PG-S3-009
DR-1-55
kDa
pY STAT3
STAT3
HSC70
pS STAT3
D
Reference
100 mM NaF
100 mM DR-1-55
100 mM DR-1-55 + 20 mM STAT3
(i)
(ii)
(iii)
(iv)
19F (ppm)
E
No STAT3
0 mM DR-1-55
25 mM DR-1-55
50 mM DR-1-55
75 mM DR-1-55
100 mM DR-1-55
Tm = 51.50° C
Tm = 48.50° C
Derivative (Arbitrary Units)
Temperature (°C)
F
Arbitrary units normalized to RPL13A
DMSO
PG-S3-009
DR-1-55
G
TRIM28
HSC70
H
sapitinib
I
Normalized counts
n.d.
J
SOCS1

Figure S4, Related to Figure 3: Analyses of STAT3, TRIM28, SOCS1 and characterization of STAT3 inhibitor DR-1-55

(**A** and **B**) Quantification of pS-STAT3 (**A**) and pY-STAT3 (**B**) normalized to HSC70 and total STAT3 from Western blots of total STAT3, pS-STAT3, pY-STAT3 shown in **Figure 3A**. Statistical significance was calculated by unpaired t-test. Bars represent mean +/- SEM. (**C**) Western blots of total STAT3, pS-STAT3 and pY-STAT3 upon treatment with the STAT3 inhibitors PG-S3-009 (2 µM) and DR-1-55 (4 µM). (**D**) ^{19}F NMR spectra of (i) 100 µM reference (5-fluorotryptophan). (ii) 100 µM sodium fluoride with reference. (iii) 100 µM DR-1-55 with reference. (iv) 100 µM DR-1-55 and with 20 µM STAT3 protein and the reference. All samples were prepared in PBS buffer with 2% glycerol, 10% DMSO and 10% D_2O. (**E**) Thermal shift assays of 2 µM STAT3 protein with varying concentrations of DR-1-55. STAT3 was incubated with varying concentrations of DR-1-55 for 10 min and Sypro orange (at a final concentration of 5x) was introduced to the sample well. The temperature was increased in 0.5 ^{o}C increments from 20 – 80 ^{o}C and the fluorescence intensity was recorded at each step. The first derivative plot of the melting curve was determined and the denaturation temperature (local maxima) was calculated from the average of three independent measurements and a representative denaturation curve is shown. (**F**) *MMP2* was measured by real-time PCR from DFTD cells treated with PG-S3-009, DR-1-55 or DMSO as control for 24 hours (n=3 replicates). One out of two similar experiments shown. Statistical significance was calculated by One-way ANOVA. Bars represent mean +/- SEM. (**G**) Western blot for TRIM28 from DFTD cell lines and Tasmanian devil fibroblasts. (**H**) *TRIM28* was measured by real-time PCR from DFTD cells treated with sapitinib, PG-S3-009, DR-1-55 or DMSO as control for 24 hours (n=3 replicates). One out of two similar experiments shown. Statistical significance was calculated by One-way ANOVA. Bars represent mean +/- SEM. (**I**) RNAseq-derived expression values of *SOCS1* from DFTD cell lines and Tasmanian devil fibroblasts. (**J**) Western blot for SOCS1 from DFTD cell lines and Tasmanian devil fibroblasts.

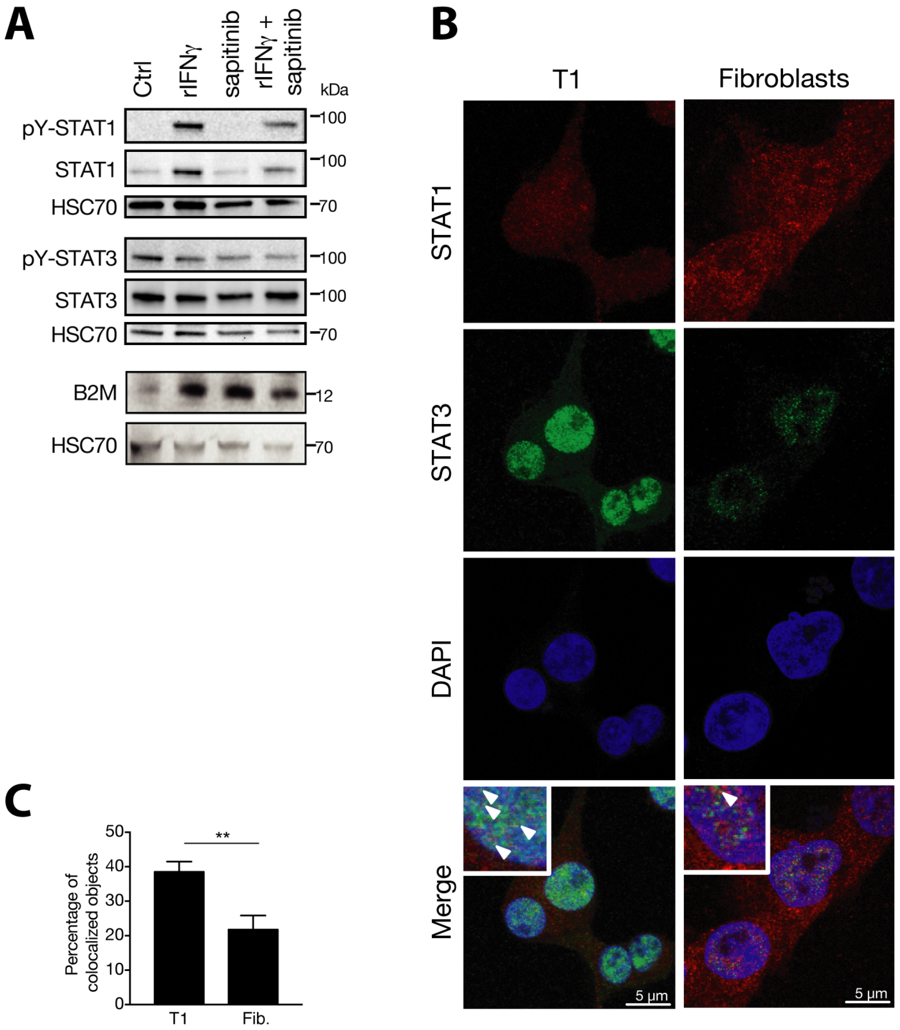

Figure S5, Related to Figure 4: Effects of recombinant interferon gamma, sapitinib and STAT3-STAT1 co-localization

(**A**) Western blots for pY-STAT1, STAT1, pY-STAT3, STAT3 and B2M from DFTD cell line (T1) upon treatment with rIFNγ, sapitinib or rIFNγ and sapitinib for 6 hours. (**B**) Representative images of immunofluorescent staining for STAT1 and STAT3 co-localization in T1 and Fib. White arrows point toward co-localization events. (**C**) Quantification of STAT3-STAT1 signal. Statistical significance was calculated by unpaired t-test. Bars represent mean +/- SEM.

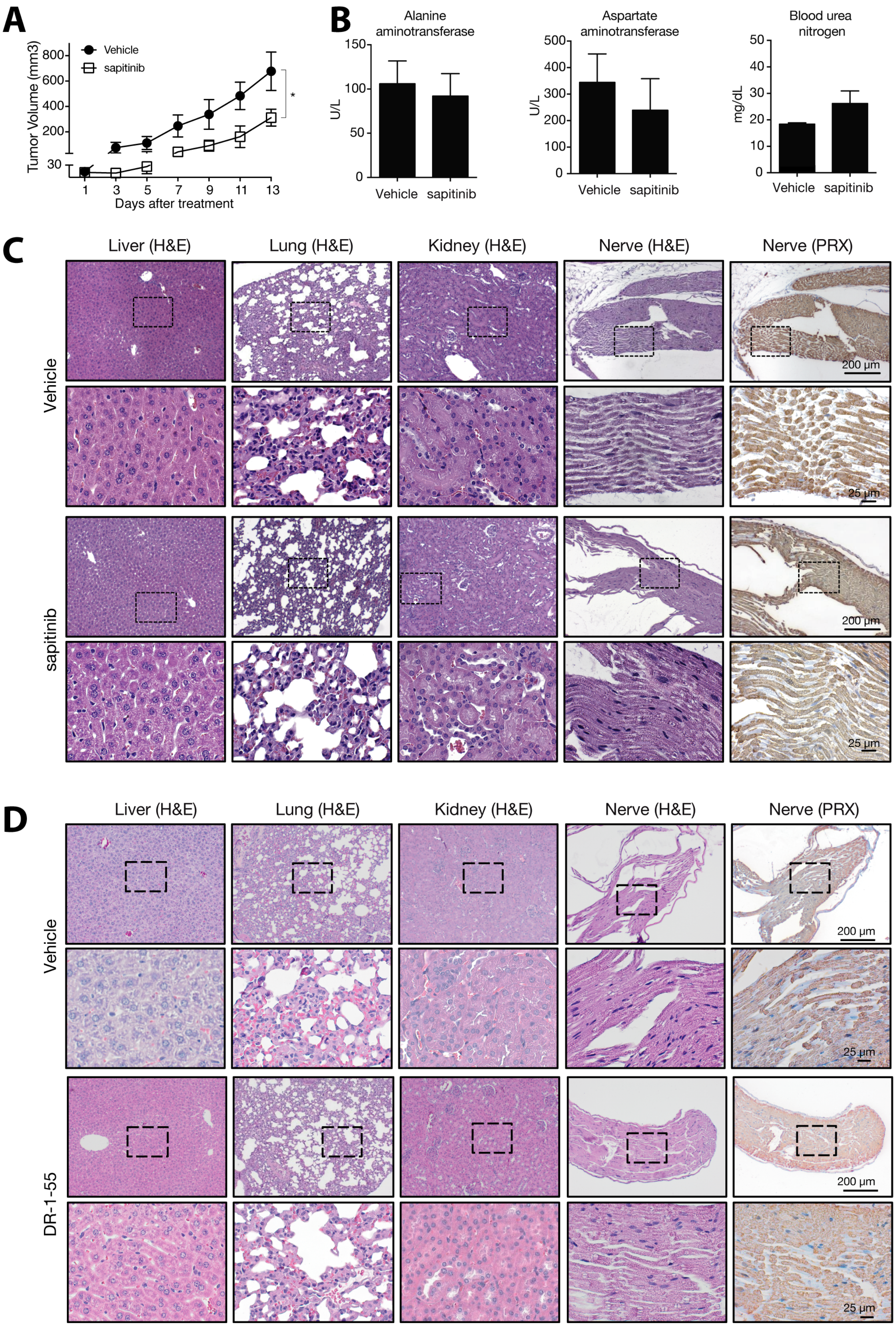

A
Tumor Volume (mm3)
800
600
400
200
30
Vehicle
sapitinib
*
1
3
5
7
9
11
13
Days after treatment
B
Alanine aminotransferase
150
100
50
0
U/L
Vehicle
sapitinib
Aspartate aminotransferase
500
400
300
200
100
0
U/L
Vehicle
sapitinib
Blood urea nitrogen
50
40
30
20
10
0
mg/dL
Vehicle
sapitinib
C
Liver (H&E)
Lung (H&E)
Kidney (H&E)
Nerve (H&E)
Nerve (PRX)
Vehicle
sapitinib
200 μm
25 μm
200 μm
25 μm
D
Liver (H&E)
Lung (H&E)
Kidney (H&E)
Nerve (H&E)
Nerve (PRX)
Vehicle
DR-1-55
200 μm
25 μm
200 μm
25 μm

Figure S6, Related to Figures 4 and 5: Pathologic examination of xenograft mouse models

(**A**) Tumor volume of NSG mice transplanted with DFTD tumor cells 27 days prior to treatment with either vehicle or 50 mg/kg sapitinib daily (bilateral tumors, n = 5 mice per group). Symbols represent mean +/- SEM. (**B**) Serum concentration of alanine aminotransferase, aspartate aminotransferase and blood urea nitrogen from mice treated with either vehicle or 50 mg/kg sapitinib daily for 13 days. Bars represent the mean of 3 to 5 mice +/- SEM. (**C**) H&E stainings for liver, lung, kidney and nerve tissue of mice treated with either vehicle or 50 mg/kg sapitinib daily for 13 days. Nerve tissue was also immunohistochemically stained for Periaxin (PRX). Scale bars, 200 µm and 25 µm. (**D**) H&E stainings for liver, lung, kidney and nerve tissue of mice treated with either vehicle or 10 mg/kg DR-1-55 each second day. Nerve tissue was also immunohistochemically stained for Periaxin (PRX). Scale bars, 200 µm and 25 µm. Statistical significance was calculated by (**A**) Two-way ANOVA with Bonferroni correction or (**B**) unpaired t-test.
