# Supplementary material for: The ERBB-STAT3 Axis Drives Tasmanian Devil Facial Tumor Disease
Source: Cancer Cell. 2019 Jan 14;35(1):125–139.e9. doi: 10.1016/j.ccell.2018.11.018 (PMC6335503; doi:10.1016/j.ccell.2018.11.018)
Supplement: Document S2. Article plus Supplemental Information [file mmc9.pdf]

Article

Cancer Cell

The ERBB-STAT3 Axis Drives Tasmanian Devil Facial Tumor Disease

Graphical Abstract

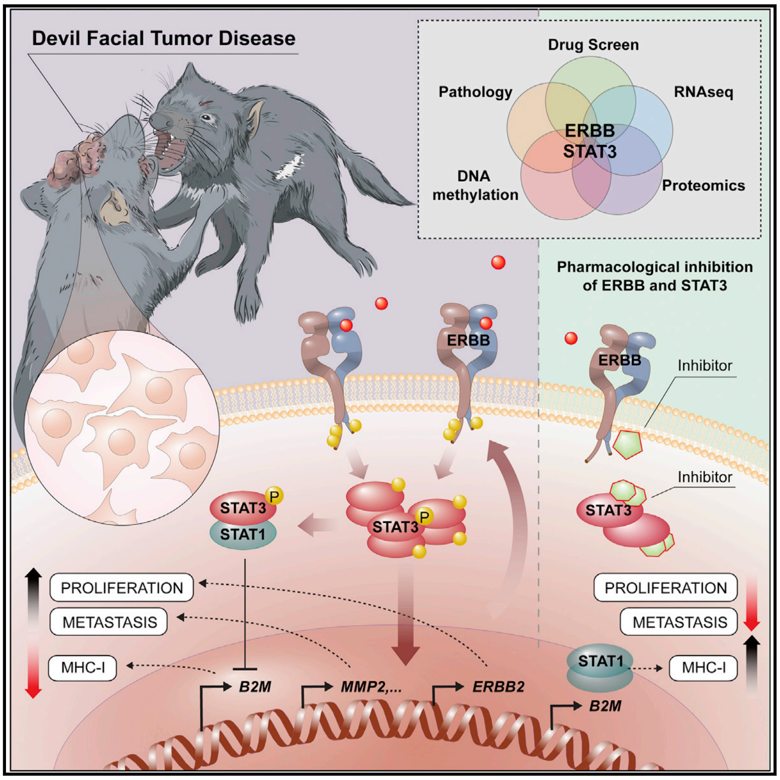

Highlights

- Tasmanian devil proteome and DNA methylome reveal modulated tumor environment
- Identification of cancer-driving hyperactive ERBB-STAT3 axis as a druggable target
- ERBB-STAT3-driven suppression of MHC class I genes may facilitate immune evasion
- Successful pharmacological treatments of Tasmanian devil tumors in a xenograft model

Authors

Lindsay Kosack, Bettina Wingelhofer, Alexandra Popa, ..., Keiryn L. Bennett, Richard Moriggl, Andreas Bergthaler

Correspondence

abergthaler@cemm.oeaw.ac.at

In Brief

Kosack et al. identify the ERBB-STAT3 signaling axis as a central molecular driver of Tasmanian devil transmissible facial tumors. Inhibition of ERBB or STAT3 prevents tumor growth in xenograft models and restores MHC class I expression, suggesting a chemo-immunotherapeutic strategy to save Tasmanian devils.

Kosack et al., 2019, Cancer Cell *35*, 125–139
January 14, 2019 © 2018 The Authors. Published by Elsevier Inc.
https://doi.org/10.1016/j.ccell.2018.11.018

CellPress

CellPress

The ERBB-STAT3 Axis Drives Tasmanian Devil Facial Tumor Disease

Lindsay Kosack,[1,11] Bettina Wingelhofer,[2,11] Alexandra Popa,[1,11] Anna Orlova,[2,3,11] Benedikt Agerer,[1] Bojan Vilagos,[1] Peter Majek,[1] Katja Parapatics,[1] Alexander Lercher,[1] Anna Ringler,[1] Johanna Klughammer,[1] Mark Smyth,[1] Kseniya Khamina,[1] Hatoon Baazim,[1] Elvin D. de Araujo,[4] David A. Rosa,[4] Jisung Park,[4] Gary Tin,[4] Siawash Ahmar,[4] Patrick T. Gunning,[4] Christoph Bock,[1,5,6] Hannah V. Siddle,[7] Gregory M. Woods,[8] Stefan Kubicek,[1] Elizabeth P. Murchison,[9] Keiryn L. Bennett,[1] Richard Moriggl,[2,3,10,12] and Andreas Bergthaler[1,12,13,*]
[1]CeMM Research Center for Molecular Medicine of the Austrian Academy of Sciences, 1090 Vienna, Austria
[2]Institute of Animal Breeding and Genetics, University of Veterinary Medicine Vienna, 1210 Vienna, Austria
[3]Ludwig Boltzmann Institute for Cancer Research, 1090 Vienna, Austria
[4]University of Toronto, Mississauga, ON L5L 1C6, Canada
[5]Department of Laboratory Medicine, Medical University of Vienna, 1090 Vienna, Austria
[6]Max Planck Institute for Informatics, Saarland Informatics Campus, 66123 Saarbrücken, Germany
[7]Department of Biological Science, University of Southampton, Southampton SO17 1BJ, UK
[8]Menzies Institute for Medical Research, University of Tasmania, Hobart, TAS 7000, Australia
[9]Transmissible Cancer Group, Department of Veterinary Medicine, University of Cambridge, Cambridge CB3 0ES, UK
[10]Medical University of Vienna, 1090 Vienna, Austria
[11]These authors contributed equally
[12]Senior author
[13]Lead Contact
*Correspondence: abergthaler@cemm.oeaw.ac.at
https://doi.org/10.1016/j.ccell.2018.11.018

SUMMARY

The marsupial Tasmanian devil (*Sarcophilus harrisii*) faces extinction due to transmissible devil facial tumor disease (DFTD). To unveil the molecular underpinnings of this transmissible cancer, we combined pharmacological screens with an integrated systems-biology characterization. Sensitivity to inhibitors of ERBB tyrosine kinases correlated with their overexpression. Proteomic and DNA methylation analyses revealed tumor-specific signatures linked to the evolutionary conserved oncogenic STAT3. ERBB inhibition blocked phosphorylation of STAT3 and arrested cancer cells. Pharmacological blockade of ERBB or STAT3 prevented tumor growth in xenograft models and restored MHC class I expression. This link between the hyperactive ERBB-STAT3 axis and major histocompatibility complex class I-mediated tumor immunosurveillance provides mechanistic insights into horizontal transmissibility and puts forward a dual chemo-immunotherapeutic strategy to save Tasmanian devils from DFTD.

INTRODUCTION

Cancer cells do not usually transmit between individuals. No human examples of transmissible cancers are known apart from rare iatrogenic cases during surgery and transplantation or materno-fetal transmission (Isoda et al., 2009; Matser et al., 2018; Metzger and Goff, 2016). Accordingly, horizontal transmission is not considered a hallmark of cancer (Hanahan and Weinberg, 2011). Yet, at least six species in the animal kingdom harbor clonal cancers which spread horizontally within populations (Metzger et al., 2016; Ostrander et al., 2016). These diseases include the fatal devil facial tumor disease (DFTD) in Tasmanian

Significance

Tasmanian devils are threatened with extinction by a horizontally transmitted cancer termed devil facial tumor disease (DFTD). Using an integrative systems-wide approach, this study identifies the ERBB-STAT3 signaling axis as a central molecular driver of DFTD. Pharmacological targeting of this axis in cell culture and in xenograft mouse models inhibited tumor growth and restored the expression of MHC class I genes. These results extend our understanding of the molecular signaling cascades driving DFTD and provide a potential explanation why horizontally transmitted DFTD cells are not rejected by the immune system. Pharmacological interference with ERBB-STAT3 may provide a much-needed addition to the ongoing efforts to save Tasmanian devils from transmissible cancer.

This is an open access article under the CC BY license (http://creativecommons.org/licenses/by/4.0/).

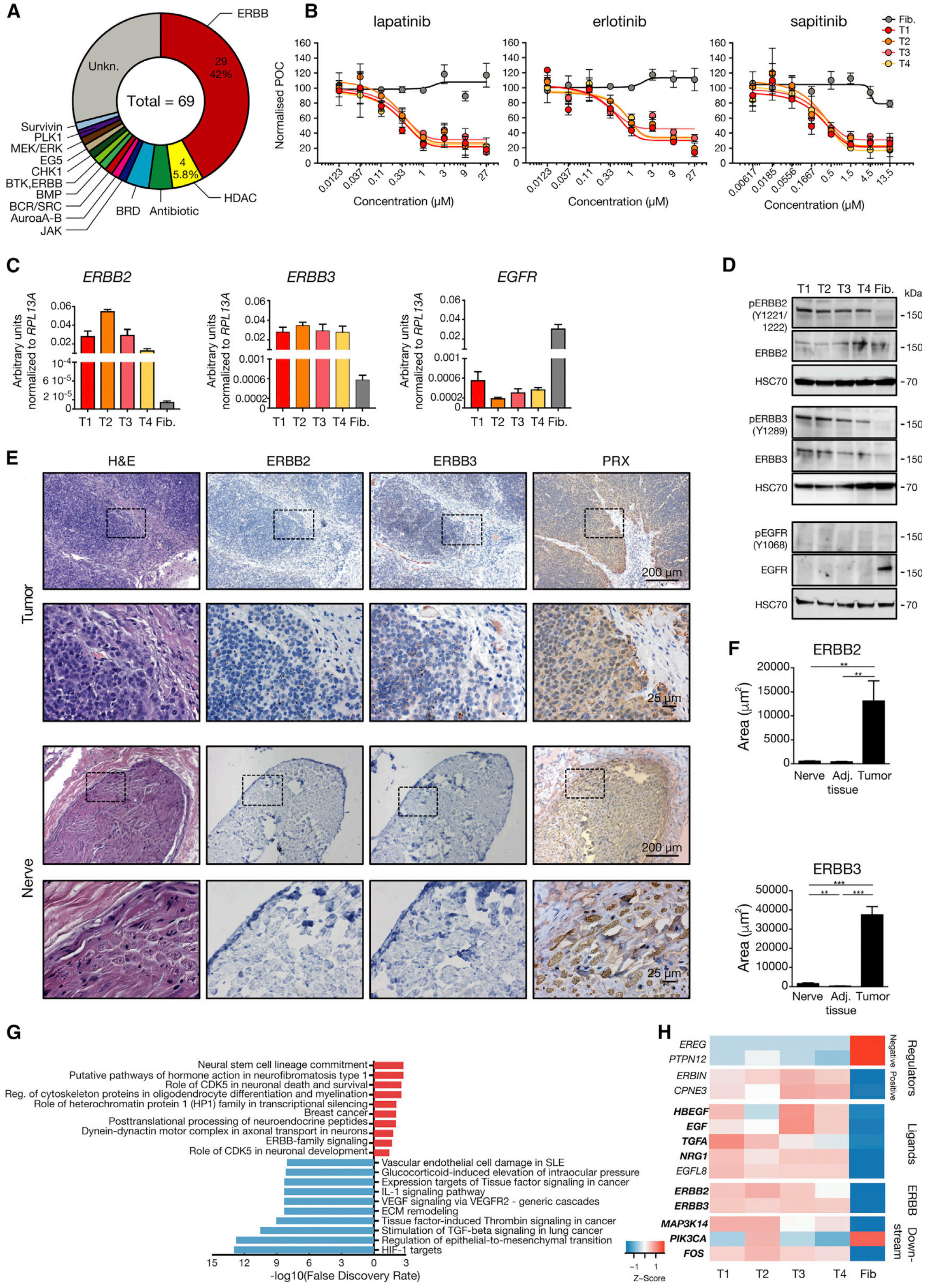

Figure 1. A Pharmacological Screen Identified ERBB-Specific Vulnerability of DFTD

(A) The targets of the 69 drug hits in the 4-point drug screen showing a reduction in cell viability in at least 1 of the 4 DFTD cell lines compared with healthy fibroblasts.

(legend continued on next page)

devils (Murchison et al., 2010, 2012; Pearse and Swift, 2006; Stammnitz et al., 2018), a sexually transmitted sarcoma in dogs (Frampton et al., 2018; Murchison et al., 2014; Murgia et al., 2006), and leukemia-like cancers in mollusks (Metzger et al., 2015, 2016). Genetic studies provided invaluable insights into these unusual cancers but the molecular underpinnings of malignancy and transmissibility remain poorly understood.

DFTD is an allogeneic graft of Schwann cell origin, which is transmitted by direct transfer of living cancer cells from one individual to another as a result of biting behavior during feeding or mating interactions (Murchison et al., 2010; Pearse and Swift, 2006). Diseased devils succumb to the disease within months (Loh et al., 2006a), rendering DFTD a serious threat to the survival of the population of the largest living marsupial carnivore. Several mechanisms are thought to confer the tumor cells with the property of being successfully transmitted between individual Tasmanian devils, including the lack of rejection due to the low expression levels of major histocompatibility complex (MHC) class I genes and diminished genetic diversity (Siddle et al., 2007, 2013). Despite recent efforts, vaccines and treatments showed limited success against DFTD (Kreiss et al., 2015; Tovar et al., 2017). A second transmissible cancer was discovered in 2014 (Pye et al., 2016), which has been termed DFT2 to distinguish it from the first transmissible DFTD, now termed DFT1. In this study DFTD refers to DFT1, first identified in 1996 (Pearse and Swift, 2006).

In this study we investigate DFTD-specific aberrant signaling pathways in order to understand and exploit the underlying molecular wiring of this transmissible cancer for potential therapeutic avenues.

RESULTS

ERBB-Specific Vulnerability of DFTD Identified by Pharmacological Screening

To address the inherent limitations of working with non-model organisms such as the Tasmanian devil, we characterized cell lines and primary biopsies (Table S1) through an integrative and unbiased systems-biology approach consisting of pharmacological screens, transcriptomics, proteomics, and epigenomics. First, we performed a cell viability screen with over 2,500 selected compounds against 4 DFTD tumor cell lines (T1–T4) on an automated high-throughput screening platform to identify potential pharmacological vulnerabilities (Table S2). A fibroblast cell line of Tasmanian devil origin served as control (Murchison et al., 2012). Sixty-nine compounds killed at least one out of four DFTD tumor cell lines, but did not affect the viability of fibroblasts, as measured by intracellular ATP levels (Figure 1A; Table S2). Interestingly, this unbiased approach yielded a substantial enrichment of tyrosine kinase inhibitors targeting selectively the ERBB receptors (29/69; 42%) including lapatinib, erlotinib, and sapitinib (Figure 1B, Table S2). In addition, we observed DFTD tumor cell-specific killing for inhibitors for histone deacetylases (HDAC), BET bromodomains and other potential therapeutic targets (Figure 1A, Table S2). The human ERBB family has four members (*EGFR*, *ERBB2*, *ERBB3*, and *ERBB4*) (Hynes and Lane, 2005), of which the devil genome has all orthologs annotated except *ERBB4*. Interestingly, DFTD cells expressed higher transcript levels of *ERBB2* and *ERBB3* compared with fibroblasts, while *EGFR* was barely detectable (Figure 1C). To validate this finding on the protein level, we tested antibodies with cross-species recognition (Table S1). Western blot analysis confirmed increased levels of total ERBB2 and ERBB3 in DFTD cell lines compared with fibroblasts (Figure 1D). The phosphorylated residues Y1221/1222 (ERBB2) and Y1289 (ERRB3) are conserved across species (Table S1), highlighting the evolutionary impact of tyrosine kinase signaling in cancer cells. Likewise, sequence alignments with >95% conserved amino acids in its protein kinase domain suggest that Tasmanian devil ERBB3 is a pseudokinase as known from other vertebrates. Phospho-site-specific antibodies provided evidence for persistent activation of ERBB2 and ERBB3 (Figure 1D). These results were corroborated by immunohistochemical detection of increased expression of ERBB2 and ERBB3 in primary tumor biopsies of diseased Tasmanian devils compared with adjacent non-tumor tissue (Figures 1E and 1F). Tumor cells were identified by the DFTD diagnostic marker Periaxin (PRX) (Murchison et al., 2010; Tovar et al., 2011). To control for the Schwann cell origin of DFTD, we also analyzed PRX-positive peripheral nerve tissue and found that DFTD tumors express elevated levels of ERBB2 and ERBB3 compared with nerve tissue (Figures 1E and F). Transcriptional profiling of DFTD tumor cell lines T1-T4 and fibroblasts revealed differentially expressed transcripts and predicted transcription factors driving this differential gene regulation (Figures S1A–S1D; Table S3). Among the predicted upregulated pathways was ERBB family signaling (Figure 1G). The genes driving this enrichment included *HBEGF*, *EGF*, and *NRG1* that encode ERBB ligands, *ERBIN* and *CPNE3* that encode positive regulators, and the proto-oncogene *FOS* (all increased expression), as well as *EREG* and *PTPN12* (decreased expression) that encode negative regulators (Figure 1G). We corroborated the differential expression of the ERBB ligands in

(B) Eight-point dose-response curves for lapatinib, erlotinib, and sapitinib in four tumor cell lines (T1–T4) and fibroblasts (Fib.) shown as normalized percentage of control (POC).

(C) Expression of the ERBB family members quantified by real-time PCR (n = 3 replicates).

(D) Western blots of the three annotated ERBB proteins (total and phosphorylated).

(E) Histopathological analysis of tumor and peripheral nerve biopsies for H&E and immunohistochemistry (IHC) against Periaxin (PRX), ERBB2, and ERBB3 on serial consecutive sections. Dotted rectangles indicate magnified areas. Scale bars, 200 and 25 μm.

(F) Quantification of ERBB2 and ERBB3 signal in tumor biopsies, adjacent tissue, and peripheral nerve tissue. Statistical significance was calculated by unpaired t test.

(G) MetaCore pathway maps enrichments separately for up- (red) and downregulated (blue) transcripts in tumor cell lines versus fibroblasts. Only the first ten pathways and with a false discovery rate ≤ 0.05 are reported.

(H) Heatmap of RNA-seq expression for genes driving the MetaCore pathway "ERBB-family signaling" (bold gene symbols) as well as known positive (*ERBIN* and *CPNE3*) and negative (*EREG* and *PTPN12*) regulators of the ERBB pathway and *EGFL8* in four DFTD cell lines (T1–T4) and fibroblasts (Fib.).

Graphs represent the mean ± SEM. See also Figure S1 and Tables S1, S2, S3, and S4.

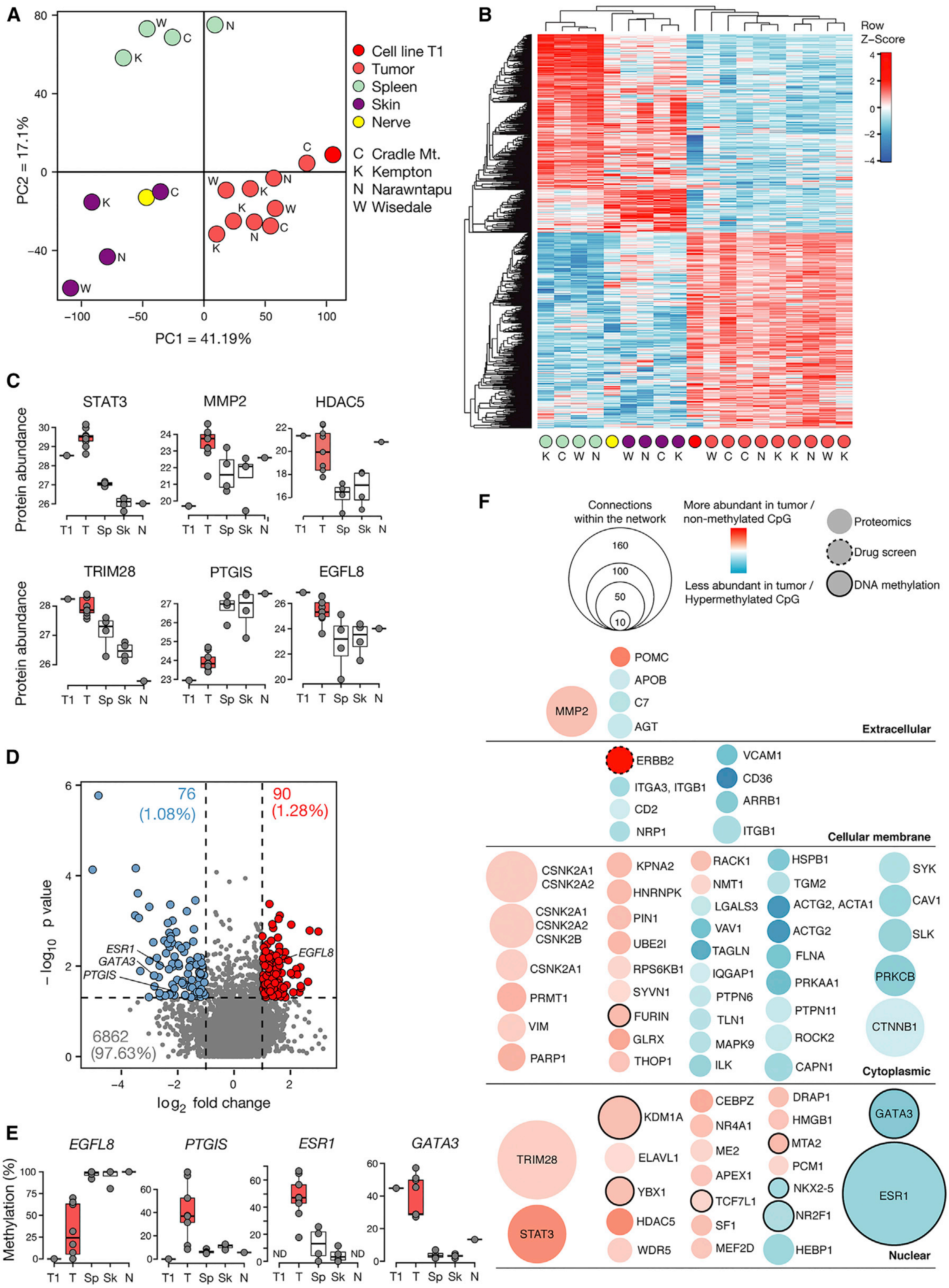

Figure 2. Integrative Systems-Level Analysis of DFTD

(A) Principal-component analysis of the 3,894 proteins quantified in all samples. Sampling locations are indicated in capital letters. Cell line denotes the DFTD cell line 06/2887 (T1) and "Nerve" stands for a healthy nerve biopsy.

(B) Hierarchical clustering of the 987 differentially modulated proteins between tumor and healthy biopsies.

(C) Boxplots of selected protein abundance across conditions in DFTD cell line (T1) and biopsies of tumor (T), spleen (Sp), skin (Sk), and nerve tissue (N).

(legend continued on next page)

primary DFTD tumor tissue by real-time PCR (Figure S1E). Due to its marked upregulation, we also included *EGFL8*, which encodes the putative ERBB ligand epithelial growth factor-like domain multiple 8, in our analysis (Figures 1H, S1E, and S1F). Of note, variant analysis of our high-coverage RNA sequencing (RNA-seq) data did not provide any evidence for activating mutations in expressed devil orthologous genes of the involved pathways (Figures S1G–S1I; Table S4). Together, these data indicate that DFTD tumor cells, which express high levels of phosphorylated ERBB2 and ERBB3 and show activation of the ERBB signaling pathway, are exquisitely vulnerable to ERBB kinase inhibitors.

Characterization of DFTD by Integrated Proteomic and DNA Methylation Analysis

To unravel the involved signaling cascades in DFTD, we investigated global changes in protein abundance in primary biopsies of diseased devils by proteomic analysis. This approach included DFTD tumor tissue as well as healthy control tissue from skin and spleen from four Tasmanian devils from different geographical locations as well as peripheral nerve tissue and the DFTD tumor cell line T1 (Table S1). Overall, we identified 6,672 unique proteins across all samples searched against a Uniprot reference library of the devil (Figure S2A, Table S5). A total of 4,981 of these identified proteins were detected across more than 80% of the samples (Figures S2B and S2C). This unbiased expression proteomic approach was not specifically designed to enrich for hydrophobic transmembrane proteins such as ERBB2 and ERBB3. Principal-component analysis of the 3,894 proteins quantified in all samples distinguished the replicates according to the tissue of origin, with the first principal component accounting for 41.1% of the inter-sample variability differentiating tumor from healthy samples (Figure 2A). Upon differential analysis of tumor versus healthy tissues we defined a tumor-modulated signature of 987 proteins (Figure 2B; Table S5). Among the most prominent proteins overexpressed in tumor tissue we identified the oncogenic transcription factor STAT3 (Figures 2C and S2D). Downstream targets of STAT3, among which matrix metalloproteinase 2 (MMP2) (Figures 2C and S2E–S2G) (Xie et al., 2004) is also differentially modulated in the tumor biopsies. We detected MMP2, which is secreted as a proprotein, at high abundance in tumor biopsies but not in the DFTD cell line. This may be due to technical limitations or could indicate its extracellular secretion in the tumor microenvironment. Tumor tissue also expressed high levels of the histone deacetylase HDAC5 and the SUMO/ubiquitin E3 ligase TRIM28, which is linked to STAT3 signaling (Tsuruma et al., 2008), as well as low expression of the tumor suppressor PTGIS (Figure 2C). Further, our proteomic analysis confirmed the high expression levels of the EGFL8 in DFTD tumor cells (Figures 2C, 1H, and S1F). Pathway enrichment analysis highlighted downregulation of processes related to chemotaxis, cell adhesion, and cytoskeleton remodeling (Figures S2H and S2I; Table S5), some of which are negatively regulated by STAT3 (Kortylewski et al., 2005; Yu et al., 2007).

In complementation to the proteomic characterization we mapped DNA methylation by reduced representation bisulfite sequencing to depict the landscape of epigenetic regulation in the aforementioned primary biopsies from Tasmanian devils. Quantification of CpG methylation was performed by an unbiased *de novo* approach (Klughammer et al., 2015). For the subsequent analysis, due to the imperfect genome annotation of this non-model organism, we focused on the identification of methylated CpGs in promoter regions. DNA methylation marks readily distinguished tumor and healthy tissue and identified tumor-specific methylation signatures and their putative transcription factor binding sites (Figures S3A–S3J). Differential analysis of tumor versus healthy biopsies highlighted 166 candidate genes with different DNA methylation levels in their promoters (Figures 2D and 2E; Table S6), which included the tumor-specific hypomethylated *EGFL8* promoter as well as hypermethylated promoters of *ESR1*, *PTGIS*, and *GATA3* (Figure 2E). Comparative analysis by gene set enrichment revealed a high concordance of RNA-seq-derived transcript levels and proteomic data in the DFTD cell line (Figure S3K) and was in line with an inverse correlation pattern of methylated promoter regions and respective gene transcript level (Figures S3L and S3M). An integrative network analysis of the identified drug vulnerabilities, protein and methylation signatures revealed a high connectivity in the molecular wiring of DFTD (Figure 2F; Table S6). This suggested a critical involvement of central oncoprotein hubs consisting of STAT3, TRIM28, and others, which may be triggered by ERBB kinase action.

Molecular Dissection of ERBB-STAT3 Axis in DFTD

The proteomic tumor signatures revealed increased levels of STAT3, which can become activated by ERBB receptor tyrosine kinase signaling, as well as an enrichment of STAT3 target genes (Figures 2C, S2E, and S2F; Table S5). Due to the central roles of STAT3 in cancer and immunity (Villarino et al., 2017; Yu et al., 2014) and the remarkably conserved amino acid sequences of STAT3 between *H. sapiens* and *S. harrisii* (99.09%), we investigated the levels of expression and activation of STAT3 by western blot. STAT3 is activated by phosphorylation of residues Y705 and S727. Thus, we tested antibodies specific to these phosphorylated residues and found highly increased

(D) Volcano plot of genes with differentially methylated promoters between healthy and tumor biopsies (hypermethylated in tumor [blue], hypomethylated in tumor [red]).

(E) Boxplot of differentially methylated gene promoters for selected genes.

(F) Direct connection proteins network among the 987 tumor-modulated proteins, 166 tumor differentially methylated gene promoters, and *ERBB2* and *ERBB3* from the drug screen and RNA-seq. The direct network interactions were built with MetaCore based on protein-protein binding, transcriptional regulation, and phosphorylation interactions. Tumor signature proteins do not have a border, while methylation candidates are represented with a black border. The ERBB2 candidate from the drug screen has a dashed black border. Of the 632 entities showing direct connectivity, only nodes with ten or more connections in MetaCore are displayed. The area of each entity is proportional to the number of connections within the network. Modulation on tumor versus healthy proteomics differential analysis, or healthy versus tumor for methylation, is colored from blue (down-modulated) to red (up-modulated).

Boxplot boundaries mark the first and third quartiles, whiskers extending to 1.5 interquartile range from the boundaries, with the median in the center. See also Figures S2 and S3 and Tables S5 and S6.

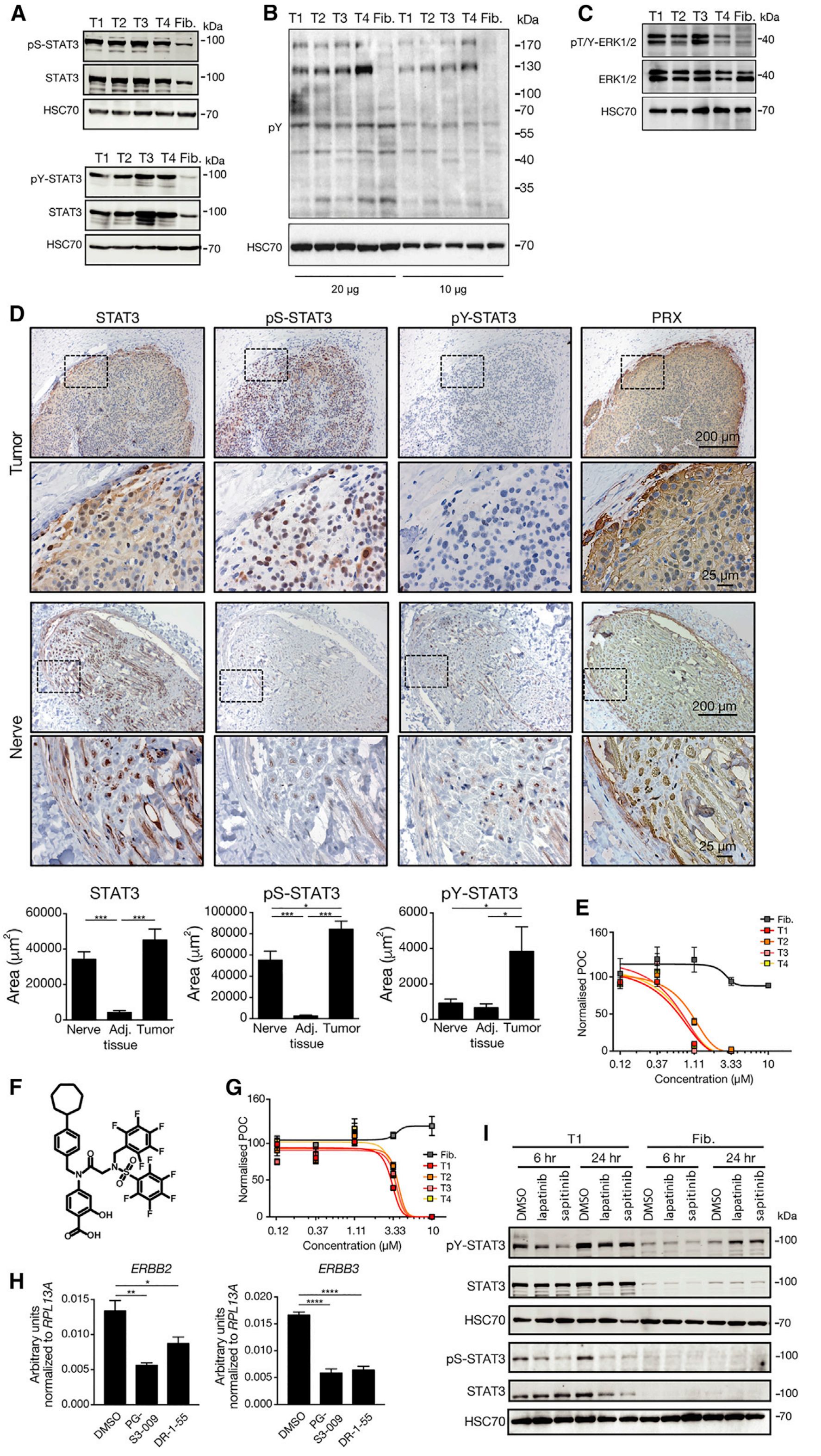

Figure 3. Molecular Dissection of ERBB-STAT3 Axis in DFTD

(A) Western blots of total STAT3, pS-STAT3, and pY-STAT3 of four DFTD cell lines (T1–T4) and fibroblasts (Fib.).

(B) Total protein phosphorylation immunoblots from lysates of four DFTD cell lines (T1–T4) and fibroblasts (Fib.) using a global anti-pY monoclonal antibody (4G10) in different input amounts.

(C) Western blots of total ERK1/2 and pT/Y-ERK1/2 of four DFTD cell lines (T1–T4) and fibroblasts (Fib.).

(D) Representative images of IHC for total STAT3, pS-STAT3, pY-STAT3, and PRX in primary tumor and peripheral nerve biopsy on serial consecutive sections and quantification of total STAT3, pS-STAT3, and pY-STAT3 in tumor biopsies, adjacent tissue, and peripheral nerve tissue. Dotted rectangles indicate magnified areas. Scale bars, 200 and 25 μm.

(E) Five-point dose-response curve of cell lines to the STAT3 inhibitor PG-S3-009 with DFTD and fibroblast cell lines.

(F) Structure of DR-1-55.

(G) Five-point dose-response curve of cell lines to the STAT3 inhibitor DR-1-55 with DFTD and fibroblast cell lines.

(H) DFTD cells treated with 2 μM PG-S3-009, 4 μM DR-1-55, or DMSO as control. Twenty-four hours after treatment, expression of *ERBB2* and *ERBB3* was measured by real-time PCR (n = 3 replicates).

(I) Western blots of total STAT3, pS-STAT3, and pY-STAT3 upon treatment with the ERBB inhibitors lapatinib (1 μM) and sapitinib (1 μM).

Statistical significance was calculated by (D and H) unpaired t test. Graphs represent the mean ± SEM. See also Figure S4.

STAT3 phosphorylation of both residues in DFTD tumor cells compared with fibroblasts (Figure 3A, S4A, and S4B). Interestingly, we also found a robust upregulation of protein tyrosine phosphorylation compared with fibroblasts (Figure 3B), indicating increased tyrosine kinase signaling. ERBB family members activate RAS-RAF-MAPK/ERK, which in turn phosphorylates STAT3 at S727 (Chung et al., 1997). Indeed, we detected increased ERK1/2 phosphorylation in DFTD tumor cells (Figure 3C). STAT3 phosphorylation was corroborated by immunohistochemical stainings in primary tumor biopsies, showing higher levels of STAT3 phosphorylation at residues S727 and Y705 compared with adjacent non-tumor tissue or peripheral nerve tissue (Figure 3D).

Treatment with the covalent STAT3-selective inhibitor PG-S3-009 (Garg et al., 2017) resulted in DFTD tumor cell-specific killing (Figure 3E) and reduced STAT3 phosphorylation of residue Y705 (Figure S4C). In addition, treatment with DR-1-55, another selective STAT3 inhibitor that covalently modifies a cysteine in the SH2 domain of STAT3 via a nucleophilic attack (Figures 3F, S4D, and S4E), reduced the expression of the STAT3 target gene *MMP2* (Figure S4F).

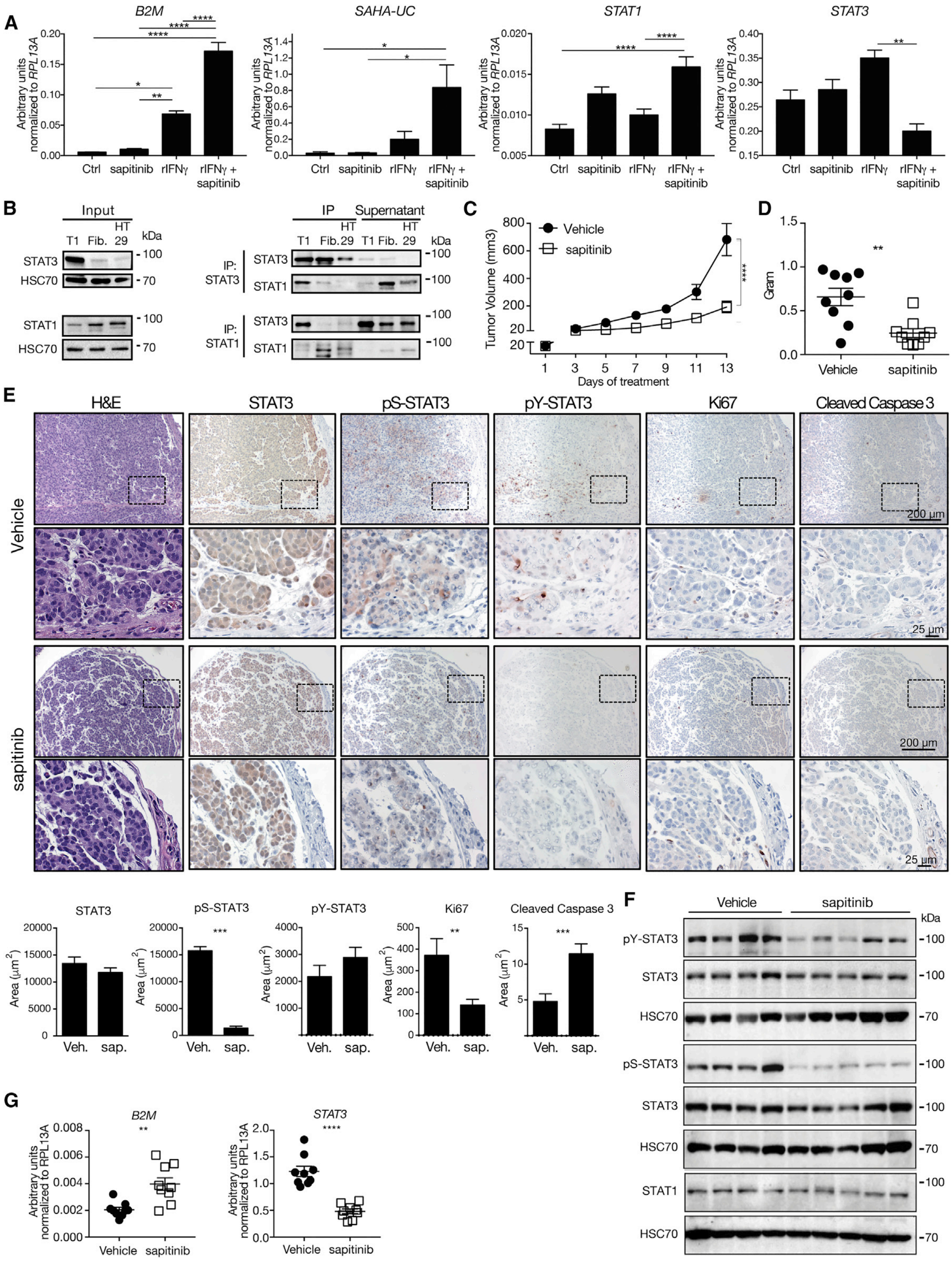

Figure 4. Blockade of ERBB Induces MHC Class I Gene Expression

(A) DFTD tumor cell line T1 was treated with recombinant interferon-γ (rIFN-γ) and/or 1 μM sapitinib. Control cells were treated with solvents. Forty-eight hours after treatment, expression of *B2M*, *SAHA-UC*, *STAT1*, and *STAT3* were measured by real-time PCR (n = 3 replicates).

(B) Reciprocal co-immunoprecipitation of STAT3 and STAT1 followed by western blots for STAT3 and STAT1 in DFTD tumor cell line (T1), fibroblasts, and human HT29 colon cancer cells as control.

(legend continued on next page)

We observed similar effects of DFTD tumor cell-specific killing with both STAT3 inhibitors (Figure 3G). Further, pharmacological inhibition of STAT3 by either PG-S3-009 or DR-1-55 led to reduced expression of *ERBB2* and *ERBB3* (Figure 3H). In line with this, DFTD tumor cells expressed higher levels of TRIM28 compared with fibroblasts (Figure S4G), and blockade of either ERBB signaling or STAT3 led to reduced transcription of *TRIM28* (Figure S4H). We also found abolished expression of the suppressor of cytokine signaling 1 (SOCS1) in DFTD tumor cells (Figures S4I and S4J), a known inhibitor of STAT3 activation (Song and Shuai, 1998). Intriguingly, treatment with ERBB inhibitors lapatinib and sapitinib inhibited serine and tyrosine phosphorylation of STAT3 (Figure 3I). In summary, our results suggest that DFTD tumor cells exhibit an ERBB-dependent constitutive activation of STAT3 in a positive feedforward loop.

Hyperactivated ERBB-STAT3 Reduces Expression of MHC Class I-Related Genes

Transmissibility of DFTD has been linked to reduced expression of MHC class I genes (Siddle and Kaufman, 2013), which prompted us to assess a potential link between hyperactive ERBB-STAT3 and MHC class I gene expression. To this end we stimulated DFTD tumor cells with recombinant autologous interferon γ (rIFN-γ). This induced the expression of *B2M* and *SAHA-UC*, one of Tasmanian devil's MHC class I genes, as shown previously (Figure 4A) (Siddle and Kaufman, 2013). Treatment with the ERBB inhibitor sapitinib alone was insufficient to induce MHC class I genes, which suggested an additional signaling requirement. Importantly, concomitant treatment of rIFN-γ with sapitinib amplified increased expression of *B2M* and *SAHA-UC* (Figures 4A and 4B). In addition, we observed increased expression of *STAT1* and a trend toward reduced *STAT3* expression upon treatment with rIFN-γ and sapitinib (Figures 4A and S5A). *B2M* and *SAHA-UC* are bona fide STAT1 target genes. Thus, we hypothesized that high levels of STAT3 may interfere with STAT1 transcriptional regulation. Indeed, reciprocal co-immunoprecipitation confirmed physical interaction between STAT3 and STAT1 (Figure 4B). This was corroborated by co-localization of STAT3 and STAT1 in DFTD tumor cells (Figures S5B and S5C).

To test the effects of ERBB inhibition *in vivo*, we transplanted DFTD tumor cells (T1) subcutaneously into the flanks of NOD/SCID gamma (NSG) mice and, 21 days later when tumors were palpable, started to administer 50 mg/kg sapitinib or vehicle once daily. DFTD xenografts in the control group proliferated rapidly after transplantation, while treatment with sapitinib effectively stalled tumor growth (Figures 4C, 4D, and S6A). No drug toxicity was observed as assessed by the serum concentration of the liver transaminases alanine aminotransferase and aspartate aminotransferase and the kidney parameter blood urea nitrogen (Figure S6B) and by histopathology (Figure S6C). The observed anti-tumor effect of sapitinib was corroborated by histological analysis of tumor tissue for total STAT3, pS-STAT3, and pY-STAT3, as well as by staining for Ki67 and Cleaved Caspase 3 (Figure 4E). DFTD xenograft tumors from mice treated with sapitinib showed reduced STAT3 serine and tyrosine phosphorylation and increased expression of *B2M* (Figures 4F and 4G). Moreover, we also assessed the therapeutic effects of STAT3 inhibition by treating NSG mice 22 days after DFTD cell transplantation with 10 mg/kg DR-1-55 each second day. Similar to sapitinib, DR-1-55 resulted in reduced tumor growth (Figures 5A and 5B) without observed toxicity (Figures 5C and S6D). Treatment with DR-1-55 resulted in pronounced reduction of pY STAT3 (Figures 5D and 5E) and reduced MMP2 expression (Figure 5D). Further, DR-1-55 increased the expression of *B2M*, while *STAT3* expression was decreased (Figure 5F). In contrast to our results *in vitro* (Figure 4A), we noted that treatment of xenografted mice with sapitinib or DR-1-55 alone was sufficient to increase *B2M* (Figures 4G and 5F), possibly indicating additional host-derived signals *in vivo*. Taken together, our results derived from cell culture and xenograft models provide strong evidence for a central role of the ERBB-STAT3 axis in growth and immune evasion of DFTD.

DISCUSSION

ERBB signaling is influenced by ligand-induced activation and triggers downstream processes, such as context-dependent activation of transcriptional regulators, including members of the activator protein-1 family (AP-1, also bona fide STAT3 targets), ETS, and STAT3/5 transcription factors (Lemmon et al., 2014; Schneider and Yarden, 2016). ERBB family genes are frequently overexpressed, amplified or mutated in human solid cancers and ERBB family members are targets of clinical therapies (Appert-Collin et al., 2015; Bae and Schlessinger, 2010; Roskoski, 2014; Yarden and Pines, 2012). In Schwann cells, ERBB2/3 signaling regulates expansion and migration of progenitor cells as well as different functions in myelination and repair of axons (Corfas et al., 2004; Newbern and Birchmeier, 2010; Stassart et al., 2013). Moreover, we found increased expression of proteins associated with epithelial to mesenchymal transition (EMT) process in DFTD tumors that are also upregulated in Schwann cells upon nerve injury to support axon regeneration (Chen et al., 2007; Ferguson and Muir, 2000; Weiss et al., 2016). This included the EMT-inducing zinc-finger E-box-binding homeobox factor (ZEB2) (Comijn et al., 2001) and the STAT3 target MMP2, which enhances the degradation of extracellular matrix proteins and cancer cell invasion (Nistico et al., 2012). The regenerative properties of Schwann cells are highly linked to their plasticity whereby, upon nerve injury, they reversibly de-differentiate, acquire high motility,

(C and D) Tumor volume (C) and tumor weight (D) of DFTD tumor cell line T1 transplanted into NSG mice and treated with either vehicle or 50 mg/kg sapitinib once daily (bilateral tumors, n = 5 mice per group). One out of two representative experiments is shown.

(E) H&E and IHC analyses for total STAT3, pS-STAT3, pY-STAT3, Ki67, and Cleaved Caspase 3 of tumor tissues. Pictures shown are from contiguous sections. Dotted rectangles indicate magnified areas. Quantification of total STAT3, pS-STAT3, pY-STAT3, Ki67, and Cleaved Caspase 3. Scale bars, 200 and 25 μm.

(F) Western blots for total STAT3, pY-STAT3, pS-STAT3, and STAT1 from representative xenograft tumors.

(G) Expression of *B2M* and *STAT3* by real-time PCR from xenograft tumor tissue.

Statistical significance was calculated by (A) one-way or (C) two-way ANOVA with Bonferroni correction or (D, E, and G) unpaired t test. Graphs represent the mean ± SEM. See also Figures S5 and S6.

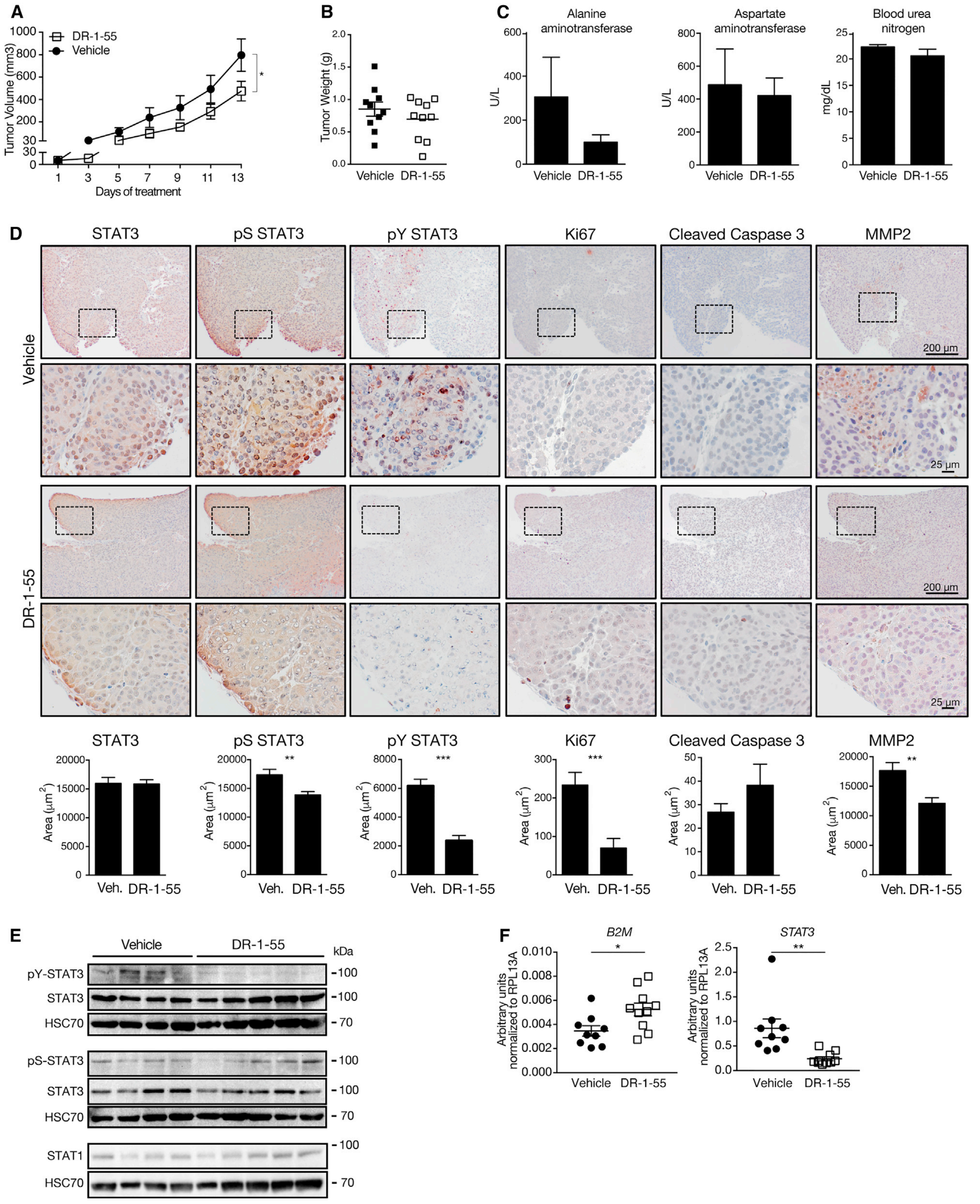

Figure 5. Xenograft Model with STAT3 Inhibitor DR-1-55

(A and B) Tumor volume (A) and tumor weight (B) of NSG mice transplanted with DFTD tumor cell line T1 and, 22 days after transplantation, treated with either vehicle or 10 mg/kg DR-1-55 each day (bilateral tumors, n = 5 mice per group).

(legend continued on next page)

and guide the growth of the damaged axons (Kim et al., 2013). Thus, the identification of hyperactivated ERBB-STAT3 signaling in DFTD may suggest aberrant regulation of the Schwann cell-intrinsic repair program (Arthur-Farraj et al., 2017). Of note, DFT2 tumors are pathologically similar but stain-negative for the Schwann cell marker PRX (Pye et al., 2016) and display copy-number gains for PDGFR (Stammnitz et al., 2018). It will be interesting to investigate the involvement of similar driver tyrosine kinase-STAT3 pathways blocking MHC class I.

A major enigmatic question of DFTD concerns the molecular properties that are required for transmission between individuals to explain the lack of rejection. Changes in MHC class I expression and diversity have been described in transmissible tumors of devils and dogs (Belov, 2011). Of note, recent immunotherapy trials with MHC-induced DFTD cells showed immunogenicity *in vivo* (Tovar et al., 2017). Our data indicate that targeting of the hyperactivated ERBB-STAT3 axis re-establishes the expression of MHC class I, thus facilitating MHC-mediated tumor immunosurveillance in Tasmanian devils (Figure 6). Promoters of interferon-stimulated MHC class I-related genes are targets for STAT1, while STAT3 is known to interfere with transcription by sequestering STAT1 in the cytoplasm through heterodimerization (Friedrich et al., 2017; Nivarthi et al., 2016; Stancato et al., 1996). We hypothesize that the endogenous tonic interferon-STAT-MHC class I axis in DFTD is disrupted due to high STAT3 action promoting cancer cell proliferation, survival, and invasion. Interestingly, STAT3 overexpression was previously proposed as one of several candidate mechanisms of immune evasion in transplantation and transmissible tumors (Fassati and Mitchison, 2010). Next to the potential interference of STAT3 with expression of MHC-I, there may also be a role for TRIM28, which we found to be overexpressed in DFTD and which acts as a negative regulator of interferon signaling (Liang et al., 2011). Of note, reduced expression of MHC-I is expected to impair rejection by $CD8^+$ T cells only but not by natural killer cells, which recognize cells in the absence of MHC-I. Natural killer cells of Tasmanian devils, however, lack the ability to directly recognize DFTD cells (Brown et al., 2011, 2016; Peel and Belov, 2018), which is likely to contribute to the lack of rejection of horizontally transmitted tumor cells. Together, our findings may bear relevance also for other transmissible cancers in higher organisms including dogs, whose transmissible tumor lacks B2M and shows low MHC class I surface expression (Cohen et al., 1984; Murgia et al., 2006).

We wish to acknowledge that molecular investigations of non-model organisms can be notoriously hampered by imperfect genome annotations, orthology inferences, and lack of reagents. Several layers of controls were included in this integrative and unbiased systems-biology approach. This was complemented by comparative pathology for which we exploited the high degree of conservation of key oncoproteins driving human cancer, cross-validating a similar driver oncogene scenario in Tasmanian devils.

Our data indicate a positive feedback loop between ERBB receptor tyrosine kinases and STAT3. The underlying mechanism of maintained STAT3 activation, the role of specific STAT3-activating kinases and the apparent lack of negative regulation requires further investigation. Notably, STAT3 S727 phosphorylation is often constitutive in cancer cells associated with hyperactive RAS-RAF signaling, and modulates ATP production in mitochondria through the respiratory chain (Gough et al., 2009; Wegrzyn et al., 2009). Serine phosphorylation of STAT3 can be catalyzed by many kinases including p38 MAPK, ERK, JNK, CDKs, mTOR, or PKC in response to growth factors such as EGF, PDGF, or Insulin (Chung et al., 1997). Sapitinib efficiently blocked S727 phosphorylation of STAT3 in DFTD cells, while STAT3 inhibition tended to show stronger effects on phosphorylation at residue Y705. Thus, an inhibition of growth factor signaling through sapitinib combined with STAT3 inhibition could be beneficial to synergistically block survival, proliferation, and metabolic functions of STAT3. Not mutually exclusively, the involved processes may include recently detected copy gains of ERBB3 (Hayes et al., 2017; Taylor et al., 2017) leading to enhanced ERBB2-ERBB3 heterodimer activity, the secretion of ligands of the ERBB family, and/or blunted negative regulation by phosphatases or the SOCS pathway.

Histopathologically, DFTD presents as undifferentiated pleomorphic tumor cells with fibrous stroma and occasional infiltrating immune cells (Howson et al., 2014; Loh et al., 2006a, 2006b). It is, thus, conceivable that the complex underlying interactions within the tumor microenvironment may contribute to the activation of the ERBB-STAT3 axis. The integrated and unbiased systems-level analysis presented here is expected to provide a critical foundation for further investigations and raises general questions about the tumor biology and transmissibility of such cancers in other species. The implicated canonical cancer signatures apparently do not suffice to give rise to transmissible cancers in humans. Thus, it should be emphasized that the occurrence of transmissible cancers in mammalians is likely to depend on a complex combination of molecular as well as non-molecular context-dependent features, such as aggressive behavior, tissue wounding, and population dynamics (Epstein et al., 2016).

Blocking the ERBB-STAT3 axis may present a promising drug target whose interference arrests cancer cells and at the same time leads to increased tumor surveillance through re-expression of MHC class I (Garrido et al., 2016). While pharmacological treatments come with inherent logistic limitations for wildlife diseases, this rationalized therapeutic strategy—possibly in combination with a vaccine against DFTD and/or immunotherapeutic interventions—offers a much-needed expansion of the so far limited measures to preserve the Tasmanian devil from extinction.

(C) Serum concentration of alanine aminotransferase, aspartate aminotransferase, and blood urea nitrogen.
(D) Tumor tissue immunohistochemically stained and quantified for total STAT3, pS-STAT3, pY-STAT3, Ki67, Cleaved Caspase 3, and MMP2. Pictures shown are from contiguous sections. Dotted rectangles indicate magnified areas. Scale bars, 200 and 25 μm.
(E) Western blots for total STAT3, pY-STAT3, pS-STAT3, and STAT1 from representative xenograft tumors.
(F) Expression of *B2M* and *STAT3* by real-time PCR from xenograft tumor tissue.
Statistical significance was calculated by (A) two-way ANOVA with Bonferroni correction or (B–D and F) unpaired t test. Graphs represent the mean ± SEM. See also Figure S6.

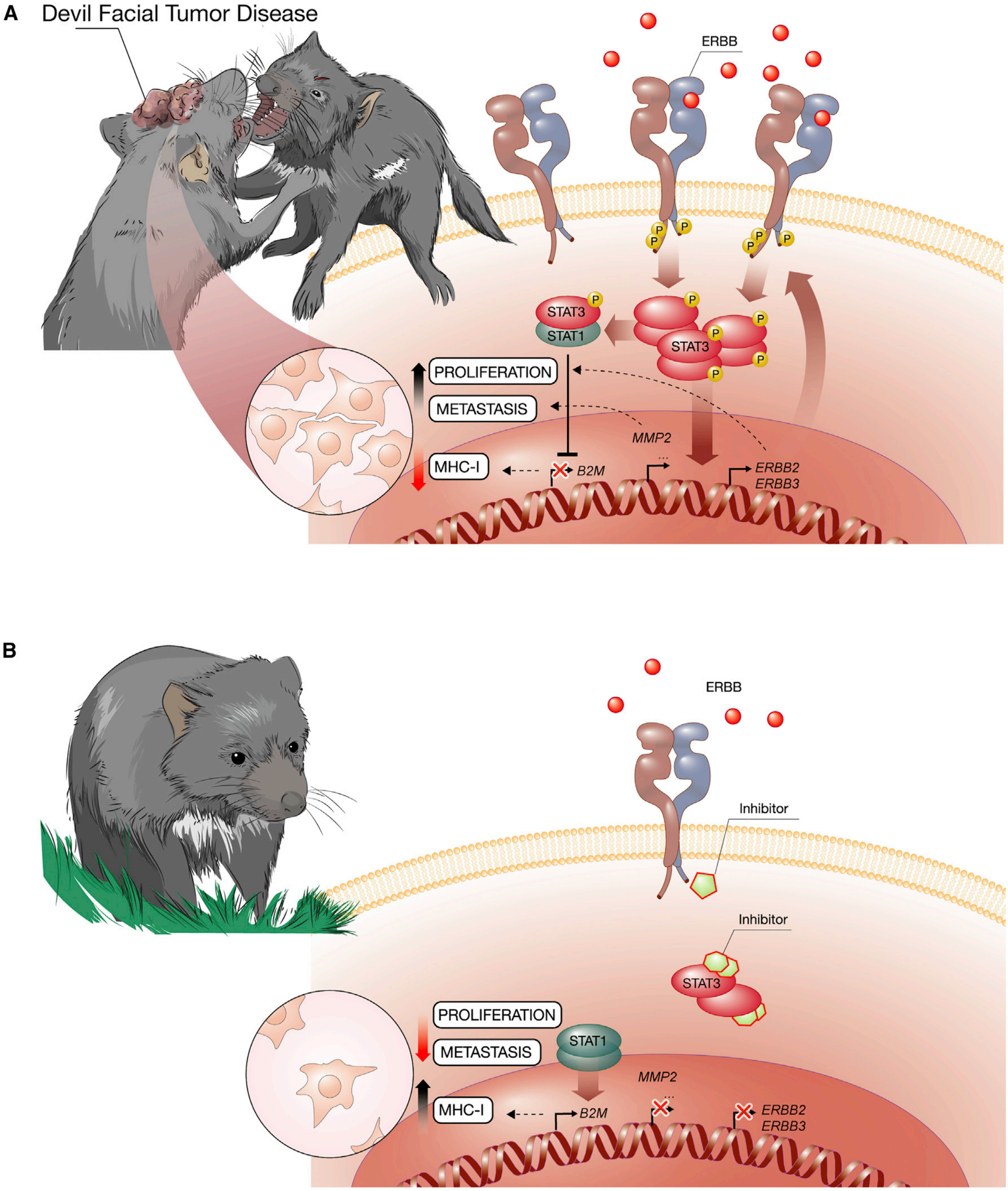

Figure 6. Working Model for the Impact of the ERBB-STAT3 Axis in DFTD

(A) Aggressive social interactions in the highly inbred population of Tasmanian devils enabled the rapid spread of DFTD with fatal consequences. The hyperactive ERBB-STAT3 axis induces the expression of downstream metastasis-related genes (i.e., *MMP2*) while suppressing the expression of MHC class I genes (i.e., *B2M*). We hypothesize that highly abundant phosphorylated STAT3 protein traps unphosphorylated STAT1 proteins in heterodimers, thereby preventing the transcriptional regulation of STAT1 downstream target genes such as *B2M*. This may contribute to immune evasion and the known lack of tumor rejection upon horizontal transmission.

(B) Interference with the ERBB-STAT3 axis by using either ERBB inhibitors or STAT3 inhibitors results in killing of DFTD tumor cells.

STAR★METHODS

Detailed methods are provided in the online version of this paper and include the following:

- KEY RESOURCES TABLE
- CONTACT FOR REAGENT AND RESOURCE SHARING
- EXPERIMENTAL MODEL AND SUBJECT DETAILS
  - Cell Culture and Tissue Biopsies
  - Mouse Xenograft Studies
- METHODS DETAILS
  - Drug Viability Screen
  - DNA and RNA Isolation
  - Real-Time PCR
  - Western Blotting
  - Immunoprecipitation
  - Histology
  - Immunofluorescence
  - Mass-Spectrometry Based Proteomics
  - DNA Methylation Analysis
  - Transcriptome Expression Analysis and Variant Calling
  - Measurement of Biochemistry Parameters
- QUANTIFICATION AND STATISTICAL ANALYSIS
  - Immunohistochemistry and Immunofluorescence Quantification
  - Mass-Spectrometry Based Proteomics - Quantification
  - DNA Methylation Analysis and Quantification
  - Western Blot Quantification
  - Transcriptome Expression Analysis and Variant Calling
  - Network Analysis
  - Motif Enrichment Analysis
  - Integrating DFTD Quantifications across Techniques
  - Characterization of the STAT3 Inhibitor DR-1-55
- DATA AND SOFTWARE AVAILABILITY

SUPPLEMENTAL INFORMATION

Supplemental Information includes six figures, six tables, and one video and can be found with this article online at https://doi.org/10.1016/j.ccell.2018.11.018.

A video abstract is available at https://doi.org/10.1016/j.ccell.2018.11.018#mmc8.

ACKNOWLEDGMENTS

We thank Thomas Penz and the Biomedical Sequencing Facility at CeMM and Fiorella Schischlik with technical support for sequencing and analysis, Kathrin Runggatscher for technical support with the drug screen, Young Mi Kwon and Jinhong Wang with logistical support, and Michael Moschinger and Theresa Pinter for discussions. We thank Safia Zahma for histopathology and immunohistochemistry processing, and Thomas Grunt and Herwig Moll for advice on reagents for ERBB family members. The authors would like to thank Ruedi Aebersold, Lukas Flatz, Stephen P. Goff, Nancy Hynes, Christopher Schliehe, Giulio Superti-Furga, and Rolf M. Zinkernagel for valuable feedback and discussions.

This work was supported by funding from the Austrian Academy of Sciences (to A.B.), the Austrian Science Fund (FWF) DK W1212 (to B.A. and A.B.), and the European Research Council (ERC) under the European Union's Seventh Framework Program and Horizon 2020 research and innovation program (grant agreement no. 677006, "CMIL" to A.B.). B.W. and R.M. received funding via FWF SFB F4707-B20 and SFB-F06105. A.O. is supported by the Austrian Research Promotion Agency (FFG, no. 854452).

AUTHOR CONTRIBUTIONS

L.K., B.W., A.O., B.A., B.V., K.P., A.L., A.R., J.K., and E.D.d.A. conducted the experiments and analyzed the data. A.P., P.M., K.K., H.B., C.B., S.K., K.L.B., R.M., and A.B. analyzed the data. D.A.R., J.P., G.T., S.A., P.T.G., H.V.S., G.M.W., and E.P.M. provided the material. L.K., A.P., B.V., R.M., and A.B. wrote the manuscript. A.B. conceived and supervised the study.

DECLARATION OF INTERESTS

The authors declare no competing interests.

Received: March 15, 2018
Revised: October 5, 2018
Accepted: November 29, 2018
Published: January 14, 2019

REFERENCES

Ali, A.M., Gomez-Biagi, R.F., Rosa, D.A., Lai, P.S., Heaton, W.L., Park, J.S., Eiring, A.M., Vellore, N.A., de Araujo, E.D., et al. (2016). Disarming an electrophilic warhead: retaining potency in tyrosine kinase inhibitor (TKI)-resistant cml lines while circumventing pharmacokinetic liabilities. ChemMedChem *11*, 850–861.

Appert-Collin, A., Hubert, P., Cremel, G., and Bennasroune, A. (2015). Role of ErbB receptors in cancer cell migration and invasion. Front. Pharmacol. *6*, 283.

Arthur-Farraj, P.J., Morgan, C.C., Adamowicz, M., Gomez-Sanchez, J.A., Fazal, S.V., Beucher, A., Razzaghi, B., Mirsky, R., Jessen, K.R., and Aitman, T.J. (2017). Changes in the coding and non-coding transcriptome and DNA methylome that define the Schwann cell repair phenotype after nerve injury. Cell Rep. *20*, 2719–2734.

Assenov, Y., Muller, F., Lutsik, P., Walter, J., Lengauer, T., and Bock, C. (2014). Comprehensive analysis of DNA methylation data with RnBeads. Nat. Methods *11*, 1138–1140.

Bae, J.H., and Schlessinger, J. (2010). Asymmetric tyrosine kinase arrangements in activation or autophosphorylation of receptor tyrosine kinases. Mol. Cells *29*, 443–448.

Belov, K. (2011). The role of the major histocompatibility complex in the spread of contagious cancers. Mamm. Genome *22*, 83–90.

Bennett, K.L., Funk, M., Tschernutter, M., Breitwieser, F.P., Planyavsky, M., Ubaida Mohien, C., Muller, A., Trajanoski, Z., Colinge, J., Superti-Furga, G., and Schmidt-Erfurth, U. (2011). Proteomic analysis of human cataract aqueous humour: comparison of one-dimensional gel LCMS with two-dimensional LCMS of unlabelled and iTRAQ(R)-labelled specimens. J. Proteomics *74*, 151–166.

Bolger, A.M., Lohse, M., and Usadel, B. (2014). Trimmomatic: a flexible trimmer for Illumina sequence data. Bioinformatics *30*, 2114–2120.

Brown, G.K., Kreiss, A., Lyons, A.B., and Woods, G.M. (2011). Natural killer cell mediated cytotoxic responses in the Tasmanian devil. PLoS One *6*, e24475.

Brown, G.K., Tovar, C., Cooray, A.A., Kreiss, A., Darby, J., Murphy, J.M., Corcoran, L.M., Bettiol, S.S., Lyons, A.B., and Woods, G.M. (2016). Mitogen-activated Tasmanian devil blood mononuclear cells kill devil facial tumour disease cells. Immunol. Cell Biol. *94*, 673–679.

Chen, Z.L., Yu, W.M., and Strickland, S. (2007). Peripheral regeneration. Annu. Rev. Neurosci. *30*, 209–233.

Chung, J., Uchida, E., Grammer, T.C., and Blenis, J. (1997). STAT3 serine phosphorylation by ERK-dependent and -independent pathways negatively modulates its tyrosine phosphorylation. Mol. Cell. Biol. *17*, 6508–6516.

Cingolani, P., Platts, A., Wang le, L., Coon, M., Nguyen, T., Wang, L., Land, S.J., Lu, X., and Ruden, D.M. (2012). A program for annotating and predicting the effects of single nucleotide polymorphisms, SnpEff: SNPs in the genome of *Drosophila melanogaster* strain w1118; iso-2; iso-3. Fly (Austin) *6*, 80–92.

Cohen, D., Shalev, A., and Krup, M. (1984). Lack of beta 2-microglobulin on the surface of canine transmissible venereal tumor cells. J. Natl. Cancer Inst. *72*, 395–401.

Comijn, J., Berx, G., Vermassen, P., Verschueren, K., van Grunsven, L., Bruyneel, E., Mareel, M., Huylebroeck, D., and van Roy, F. (2001). The two-handed E box binding zinc finger protein SIP1 downregulates E-cadherin and induces invasion. Mol. Cell *7*, 1267–1278.

Corfas, G., Velardez, M.O., Ko, C.P., Ratner, N., and Peles, E. (2004). Mechanisms and roles of axon-Schwann cell interactions. J. Neurosci. *24*, 9250–9260.

de Araujo, E.D., Manaswiyoungkul, P., Israelian, J., Park, J., Yuen, K., Farhangi, S., Berger-Becvar, A., Abu-Jazar, L., and Gunning, P.T. (2017). High-throughput thermofluor-based assays for inhibitor screening of STAT SH2 domains. J. Pharm. Biomed. Anal. *143*, 159–167.

Deakin, J.E., Bender, H.S., Pearse, A.M., Rens, W., O'Brien, P.C., Ferguson-Smith, M.A., Cheng, Y., Morris, K., Taylor, R., Stuart, A., et al. (2012). Genomic restructuring in the Tasmanian devil facial tumour: chromosome painting and gene mapping provide clues to evolution of a transmissible tumour. PLoS Genet. *8*, e1002483.

Dobin, A., Davis, C.A., Schlesinger, F., Drenkow, J., Zaleski, C., Jha, S., Batut, P., Chaisson, M., and Gingeras, T.R. (2013). STAR: ultrafast universal RNA-seq aligner. Bioinformatics *29*, 15–21.

Eng, J.K., McCormack, A.L., and Yates, J.R. (1994). An approach to correlate tandem mass spectral data of peptides with amino acid sequences in a protein database. J. Am. Soc. Mass Spectrom. *5*, 976–989.

Epstein, B., Jones, M., Hamede, R., Hendricks, S., McCallum, H., Murchison, E.P., Schonfeld, B., Wiench, C., Hohenlohe, P., and Storfer, A. (2016). Rapid evolutionary response to a transmissible cancer in Tasmanian devils. Nat. Commun. *7*, 12684.

Fassati, A., and Mitchison, N.A. (2010). Testing the theory of immune selection in cancers that break the rules of transplantation. Cancer Immunol. Immunother. *59*, 643–651.

Ferguson, T.A., and Muir, D. (2000). MMP-2 and MMP-9 increase the neurite-promoting potential of Schwann cell basal laminae and are upregulated in degenerated nerve. Mol. Cell. Neurosci. *16*, 157–167.

Frampton, D., Schwenzer, H., Marino, G., Butcher, L.M., Pollara, G., Kriston-Vizi, J., Venturini, C., Austin, R., de Castro, K.F., et al. (2018). Molecular signatures of regression of the canine transmissible venereal tumor. Cancer Cell *33*, 620–633.e6.

Friedrich, K., Dolznig, H., Han, X., and Moriggl, R. (2017). Steering of carcinoma progression by the YIN/YANG interaction of STAT1/STAT3. Biosci. Trends *11*, 1–8.

Garg, N., Bakhshinyan, D., Venugopal, C., Mahendram, S., Rosa, D.A., Vijayakumar, T., Manoranjan, B., Hallett, R., McFarlane, N., Delaney, K.H., et al. (2017). CD133+ brain tumor-initiating cells are dependent on STAT3 signaling to drive medulloblastoma recurrence. Oncogene *36*, 606–617.

Garrido, F., Aptsiauri, N., Doorduijn, E.M., Garcia Lora, A.M., and van Hall, T. (2016). The urgent need to recover MHC class I in cancers for effective immunotherapy. Curr. Opin. Immunol. *39*, 44–51.

Gough, D.J., Corlett, A., Schlessinger, K., Wegrzyn, J., Larner, A.C., and Levy, D.E. (2009). Mitochondrial STAT3 supports Ras-dependent oncogenic transformation. Science *324*, 1713–1716.

Hanahan, D., and Weinberg, R.A. (2011). Hallmarks of cancer: the next generation. Cell *144*, 646–674.

Hastie, T., Tibshirani, R., Narasimhan, B., and Chu, G. (2017). Impute: Imputation for Microarray Data. R Package Version 1501. https://rdrr.io/bioc/impute/.

Hayes, D.A., Kunde, D.A., Taylor, R.L., Pyecroft, S.B., Sohal, S.S., and Snow, E.T. (2017). ERBB3: a potential serum biomarker for early detection and therapeutic target for devil facial tumour 1 (DFT1). PLoS One *12*, e0177919.

Howson, L.J., Morris, K.M., Kobayashi, T., Tovar, C., Kreiss, A., Papenfuss, A.T., Corcoran, L., Belov, K., and Woods, G.M. (2014). Identification of dendritic cells, B cell and T cell subsets in Tasmanian devil lymphoid tissue; evidence for poor immune cell infiltration into devil facial tumors. Anat. Rec. (Hoboken) *297*, 925–938.

Huber, W., von Heydebreck, A., Sultmann, H., Poustka, A., and Vingron, M. (2002). Variance stabilization applied to microarray data calibration and to the quantification of differential expression. Bioinformatics *18* (*Suppl 1*), S96–S104.

Hynes, N.E., and Lane, H.A. (2005). ERBB receptors and cancer: the complexity of targeted inhibitors. Nat. Rev. Cancer *5*, 341–354.

Ingles, E.D., and Deakin, J.E. (2015). Global DNA methylation patterns on marsupial and devil facial tumour chromosomes. Mol. Cytogenet. *8*, 74.

Isoda, T., Ford, A.M., Tomizawa, D., van Delft, F.W., de Castro, D.G., Mitsuiki, N., Score, J., Taki, T., et al. (2009). Immunologically silent cancer clone transmission from mother to offspring. Proc. Natl. Acad. Sci. U S A *106*, 17882–17885.

Kall, L., Canterbury, J.D., Weston, J., Noble, W.S., and MacCoss, M.J. (2007). Semi-supervised learning for peptide identification from shotgun proteomics datasets. Nat. Methods *4*, 923–925.

Kall, L., Storey, J.D., and Noble, W.S. (2009). QVALITY: non-parametric estimation of q-values and posterior error probabilities. Bioinformatics *25*, 964–966.

Kersey, P., Hermjakob, H., and Apweiler, R. (2000). VARSPLIC: alternatively-spliced protein sequences derived from SWISS-PROT and TrEMBL. Bioinformatics *16*, 1048–1049.

Kim, H.A., Mindos, T., and Parkinson, D.B. (2013). Plastic fantastic: Schwann cells and repair of the peripheral nervous system. Stem Cells Transl. Med. *2*, 553–557.

Klughammer, J., Datlinger, P., Printz, D., Sheffield, N.C., Farlik, M., Hadler, J., Fritsch, G., and Bock, C. (2015). Differential DNA methylation analysis without a reference genome. Cell Rep. *13*, 2621–2633.

Kortylewski, M., Kujawski, M., Wang, T., Wei, S., Zhang, S., Pilon-Thomas, S., Niu, G., Kay, H., Mule, J., Kerr, W.G., et al. (2005). Inhibiting Stat3 signaling in the hematopoietic system elicits multicomponent antitumor immunity. Nat. Med. *11*, 1314–1321.

Kreiss, A., Brown, G.K., Tovar, C., Lyons, A.B., and Woods, G.M. (2015). Evidence for induction of humoral and cytotoxic immune responses against devil facial tumor disease cells in Tasmanian devils (*Sarcophilus harrisii*) immunized with killed cell preparations. Vaccine *33*, 3016–3025.

Lemmon, M.A., Schlessinger, J., and Ferguson, K.M. (2014). The EGFR family: not so prototypical receptor tyrosine kinases. Cold Spring Harb. Perspect. Biol. *6*, a020768.

Liang, Q., Deng, H., Li, X., Wu, X., Tang, Q., Chang, T.H., Peng, H., Rauscher, F.J., 3rd, Ozato, K., and Zhu, F. (2011). Tripartite motif-containing protein 28 is a small ubiquitin-related modifier E3 ligase and negative regulator of IFN regulatory factor 7. J. Immunol. *187*, 4754–4763.

Liao, Y., Smyth, G.K., and Shi, W. (2014). featureCounts: an efficient general purpose program for assigning sequence reads to genomic features. Bioinformatics *30*, 923–930.

Loh, R., Bergfeld, J., Hayes, D., O'Hara, A., Pyecroft, S., Raidal, S., and Sharpe, R. (2006a). The pathology of devil facial tumor disease (DFTD) in Tasmanian devils (*Sarcophilus harrisii*). Vet. Pathol. *43*, 890–895.

Loh, R., Hayes, D., Mahjoor, A., O'Hara, A., Pyecroft, S., and Raidal, S. (2006b). The immunohistochemical characterization of devil facial tumor disease (DFTD) in the Tasmanian devil (*Sarcophilus harrisii*). Vet. Pathol. *43*, 896–903.

Love, M.I., Huber, W., and Anders, S. (2014). Moderated estimation of fold change and dispersion for RNA-seq data with DESeq2. Genome Biol. *15*, 550.

Mathelier, A., Zhao, X., Zhang, A.W., Parcy, F., Worsley-Hunt, R., Arenillas, D.J., Buchman, S., Chen, C.Y., Chou, A., Ienasescu, H., et al. (2014). JASPAR 2014: an extensively expanded and updated open-access database of transcription factor binding profiles. Nucleic Acids Res. *42*, D142–D147.

Matser, Y.A.H., Terpstra, M.L., Nadalin, S., Nossent, G.D., de Boer, J., van Bemmel, B.C., van Eeden, S., Budde, K., Brakemeier, S., and Bemelman, F.J. (2018). Transmission of breast cancer by a single multiorgan donor to 4 transplant recipients. Am. J. Transplant. *18*, 1810–1814.

McLeay, R.C., and Bailey, T.L. (2010). Motif Enrichment Analysis: a unified framework and an evaluation on ChIP data. BMC Bioinformatics *11*, 165.

Metzger, M.J., and Goff, S.P. (2016). A sixth modality of infectious disease: contagious cancer from devils to clams and beyond. PLoS Pathog. *12*, e1005904.

Metzger, M.J., Reinisch, C., Sherry, J., and Goff, S.P. (2015). Horizontal transmission of clonal cancer cells causes leukemia in soft-shell clams. Cell *161*, 255–263.

Metzger, M.J., Villalba, A., Carballal, M.J., Iglesias, D., Sherry, J., Reinisch, C., Muttray, A.F., Baldwin, S.A., and Goff, S.P. (2016). Widespread transmission of independent cancer lineages within multiple bivalve species. Nature *534*, 705–709.

Mootha, V.K., Lindgren, C.M., Eriksson, K.F., Subramanian, A., Sihag, S., Lehar, J., Puigserver, P., Carlsson, E., Ridderstrale, M., Laurila, E., et al. (2003). PGC-1alpha-responsive genes involved in oxidative phosphorylation are coordinately downregulated in human diabetes. Nat. Genet. *34*, 267–273.

Murchison, E.P., Schulz-Trieglaff, O.B., Ning, Z., Alexandrov, L.B., Bauer, M.J., Fu, B., Hims, M., Ding, Z., Ivakhno, S., Stewart, C., et al. (2012). Genome sequencing and analysis of the Tasmanian devil and its transmissible cancer. Cell *148*, 780–791.

Murchison, E.P., Tovar, C., Hsu, A., Bender, H.S., Kheradpour, P., Rebbeck, C.A., Obendorf, D., Conlan, C., Bahlo, M., Blizzard, C.A., et al. (2010). The Tasmanian devil transcriptome reveals Schwann cell origins of a clonally transmissible cancer. Science *327*, 84–87.

Murchison, E.P., Wedge, D.C., Alexandrov, L.B., Fu, B., Martincorena, I., Ning, Z., Tubio, J.M.C., Werner, E.I., Allen, J., De Nardi, A.B., et al. (2014). Transmissible [corrected] dog cancer genome reveals the origin and history of an ancient cell lineage. Science *343*, 437–440.

Murgia, C., Pritchard, J.K., Kim, S.Y., Fassati, A., and Weiss, R.A. (2006). Clonal origin and evolution of a transmissible cancer. Cell *126*, 477–487.

Newbern, J., and Birchmeier, C. (2010). Nrg1/ErbB signaling networks in Schwann cell development and myelination. Semin. Cell Dev. Biol. *21*, 922–928.

Nistico, P., Bissell, M.J., and Radisky, D.C. (2012). Epithelial-mesenchymal transition: general principles and pathological relevance with special emphasis on the role of matrix metalloproteinases. Cold Spring Harb. Perspect. Biol. *4*, a011908.

Nivarthi, H., Gordziel, C., Themanns, M., Kramer, N., Eberl, M., Rabe, B., Schlederer, M., Rose-John, S., Knosel, T., Kenner, L., et al. (2016). The ratio of STAT1 to STAT3 expression is a determinant of colorectal cancer growth. Oncotarget *7*, 51096–51106.

Olsen, J.V., de Godoy, L.M., Li, G., Macek, B., Mortensen, P., Pesch, R., Makarov, A., Lange, O., Horning, S., and Mann, M. (2005). Parts per million mass accuracy on an Orbitrap mass spectrometer via lock mass injection into a C-trap. Mol. Cell. Proteomics *4*, 2010–2021.

Ostrander, E.A., Davis, B.W., and Ostrander, G.K. (2016). Transmissible tumors: breaking the cancer paradigm. Trends Genet. *32*, 1–15.

Pearse, A.M., and Swift, K. (2006). Allograft theory: transmission of devil facial-tumour disease. Nature *439*, 549.

Peel, E., and Belov, K. (2018). Lessons learnt from the Tasmanian devil facial tumour regarding immune function in cancer. Mamm. Genome *29*, 731–738.

Perkins, D.N., Pappin, D.J., Creasy, D.M., and Cottrell, J.S. (1999). Probability-based protein identification by searching sequence databases using mass spectrometry data. Electrophoresis *20*, 3551–3567.

Pye, R.J., Pemberton, D., Tovar, C., Tubio, J.M., Dun, K.A., Fox, S., Darby, J., Hayes, D., Knowles, G.W., Kreiss, A., et al. (2016). A second transmissible cancer in Tasmanian devils. Proc. Natl. Acad. Sci. U S A *113*, 374–379.

Ritchie, M.E., Phipson, B., Wu, D., Hu, Y., Law, C.W., Shi, W., and Smyth, G.K. (2015). Limma powers differential expression analyses for RNA-sequencing and microarray studies. Nucleic Acids Res. *43*, e47.

Roskoski, R., Jr. (2014). The ErbB/HER family of protein-tyrosine kinases and cancer. Pharmacol. Res. *79*, 34–74.

Rouillard, A.D., Gundersen, G.W., Fernandez, N.F., Wang, Z., Monteiro, C.D., McDermott, M.G., and Ma'ayan, A. (2016). The harmonizome: a collection of processed datasets gathered to serve and mine knowledge about genes and proteins. Database (Oxford) *2016*, https://doi.org/10.1093/database/baw100.

Schneider, M.R., and Yarden, Y. (2016). The EGFR-HER2 module: a stem cell approach to understanding a prime target and driver of solid tumors. Oncogene *35*, 2949–2960.

Sdelci, S., Lardeau, C.H., Tallant, C., Klepsch, F., Klaiber, B., Bennett, J., Rathert, P., Schuster, M., Penz, T., Fedorov, O., et al. (2016). Mapping the chemical chromatin reactivation landscape identifies BRD4-TAF1 cross-talk. Nat. Chem. Biol. *12*, 504–510.

Siddle, H.V., and Kaufman, J. (2013). How the devil facial tumor disease escapes host immune responses. Oncoimmunology *2*, e25235.

Siddle, H.V., Kreiss, A., Eldridge, M.D., Noonan, E., Clarke, C.J., Pyecroft, S., Woods, G.M., and Belov, K. (2007). Transmission of a fatal clonal tumor by biting occurs due to depleted MHC diversity in a threatened carnivorous marsupial. Proc. Natl. Acad. Sci. U S A *104*, 16221–16226.

Siddle, H.V., Kreiss, A., Tovar, C., Yuen, C.K., Cheng, Y., Belov, K., Swift, K., Pearse, A.M., Hamede, R., Jones, M.E., et al. (2013). Reversible epigenetic down-regulation of MHC molecules by devil facial tumour disease illustrates immune escape by a contagious cancer. Proc. Natl. Acad. Sci. U S A *110*, 5103–5108.

Song, M.M., and Shuai, K. (1998). The suppressor of cytokine signaling (SOCS) 1 and SOCS3 but not SOCS2 proteins inhibit interferon-mediated antiviral and antiproliferative activities. J. Biol. Chem. *273*, 35056–35062.

Stammnitz, M.R., Coorens, T.H.H., Gori, K.C., Hayes, D., Fu, B., Wang, J., Martin-Herranz, D.E., Alexandrov, L.B., Baez-Ortega, A., Barthorpe, S., et al. (2018). The origins and vulnerabilities of two transmissible cancers in Tasmanian devils. Cancer Cell *33*, 607–619.e15.

Stancato, L.F., David, M., Carter-Su, C., Larner, A.C., and Pratt, W.B. (1996). Preassociation of STAT1 with STAT2 and STAT3 in separate signalling complexes prior to cytokine stimulation. J. Biol. Chem. *271*, 4134–4137.

Stassart, R.M., Fledrich, R., Velanac, V., Brinkmann, B.G., Schwab, M.H., Meijer, D., Sereda, M.W., and Nave, K.A. (2013). A role for Schwann cell-derived neuregulin-1 in remyelination. Nat. Neurosci. *16*, 48–54.

Subramanian, A., Tamayo, P., Mootha, V.K., Mukherjee, S., Ebert, B.L., Gillette, M.A., Paulovich, A., Pomeroy, S.L., Golub, T.R., Lander, E.S., and Mesirov, J.P. (2005). Gene set enrichment analysis: a knowledge-based approach for interpreting genome-wide expression profiles. Proc. Natl. Acad. Sci. U S A *102*, 15545–15550.

Taylor, R.L., Zhang, Y., Schoning, J.P., and Deakin, J.E. (2017). Identification of candidate genes for devil facial tumour disease tumourigenesis. Sci. Rep. *7*, 8761.

Tovar, C., Obendorf, D., Murchison, E.P., Papenfuss, A.T., Kreiss, A., and Woods, G.M. (2011). Tumor-specific diagnostic marker for transmissible facial tumors of Tasmanian devils: immunohistochemistry studies. Vet. Pathol. *48*, 1195–1203.

Tovar, C., Pye, R.J., Kreiss, A., Cheng, Y., Brown, G.K., Darby, J., Malley, R.C., Siddle, H.V., Skjodt, K., Kaufman, J., et al. (2017). Regression of devil facial tumour disease following immunotherapy in immunised Tasmanian devils. Sci. Rep. *7*, 43827.

Tsuruma, R., Ohbayashi, N., Kamitani, S., Ikeda, O., Sato, N., Muromoto, R., Sekine, Y., Oritani, K., and Matsuda, T. (2008). Physical and functional interactions between STAT3 and KAP1. Oncogene *27*, 3054–3059.

Van der Auwera, G.A., Carneiro, M.O., Hartl, C., Poplin, R., Del Angel, G., Levy-Moonshine, A., Jordan, T., Shakir, K., Roazen, D., Thibault, J., et al. (2013). From FastQ data to high confidence variant calls: the Genome Analysis Toolkit best practices pipeline. Curr. Protoc. Bioinformatics *43*, 11–33.

Villarino, A.V., Kanno, Y., and O'Shea, J.J. (2017). Mechanisms and consequences of Jak-STAT signaling in the immune system. Nat. Immunol. *18*, 374–384.

Wegrzyn, J., Potla, R., Chwae, Y.J., Sepuri, N.B., Zhang, Q., Koeck, T., Derecka, M., Szczepanek, K., Szelag, M., Gornicka, A., et al. (2009). Function of mitochondrial Stat3 in cellular respiration. Science *323*, 793–797.

Weiss, T., Taschner-Mandl, S., Bileck, A., Slany, A., Kromp, F., Rifatbegovic, F., Frech, C., Windhager, R., Kitzinger, H., Tzou, C.H., et al. (2016). Proteomics and transcriptomics of peripheral nerve tissue and cells unravel new aspects of the human Schwann cell repair phenotype. Glia *64*, 2133–2153.

Wingelhofer, B., Maurer, B., Heyes, E.C., Cumaraswamy, A.A., Berger-Becvar, A., de Araujo, E.D., Orlova, A., Freund, P., Ruge, F., et al. (2018). Pharmacologic inhibition of STAT5 in acute myeloid leukemia. Leukemia *32*, 1135–1146.

Wisniewski, J.R., Zougman, A., Nagaraj, N., and Mann, M. (2009). Universal sample preparation method for proteome analysis. Nat. Methods *6*, 359–362.

Xie, T.X., Wei, D., Liu, M., Gao, A.C., Ali-Osman, F., Sawaya, R., and Huang, S. (2004). Stat3 activation regulates the expression of matrix metalloproteinase-2 and tumor invasion and metastasis. Oncogene *23*, 3550–3560.

Yarden, Y., and Pines, G. (2012). The ERBB network: at last, cancer therapy meets systems biology. Nat. Rev. Cancer *12*, 553–563.

Yu, H., Kortylewski, M., and Pardoll, D. (2007). Crosstalk between cancer and immune cells: role of STAT3 in the tumour microenvironment. Nat. Rev. Immunol. *7*, 41–51.

Yu, H., Lee, H., Herrmann, A., Buettner, R., and Jove, R. (2014). Revisiting STAT3 signalling in cancer: new and unexpected biological functions. Nat. Rev. Cancer *14*, 736–746.

STAR★METHODS

KEY RESOURCES TABLE

REAGENT or RESOURCE	SOURCE	IDENTIFIER
Antibodies		
anti-HSC70 monoclonal mouse	Santa Cruz	Cat#sc-7298
anti-EGFR monoclonal mouse	Santa Cruz	Cat#sc-373746
anti-EGFR monoclonal mouse	BD Biosciences	Cat#610016
anti-phospho-EGFR (Y1068) monoclonal rabbit	Cell Signaling Technology	Cat#3777
anti-HER/ErbB2 monoclonal mouse	Santa Cruz	Cat#sc-7301
anti-HER2/ErbB2 monoclonal rabbit	Cell Signaling Technology	Cat#4290
anti-phospho-HER2/ErbB2 (Y1221/1222) monoclonal rabbit	Cell Signaling Technology	Cat#2243
anti-HER3/ErbB3 monoclonal rabbit	Cell Signaling Technology	Cat#12708
anti-phospho-HER3/ErbB3 (Y1289) monoclonal rabbit	Cell Signaling Technology	Cat#2842
anti-Periaxin/PRX	Sigma Aldrich	Cat#HPA001868
anti-STAT3 monoclonal mouse	BD Biosciences	Cat#610189
anti-STAT3 monoclonal mouse	Cell Signaling Technology	Cat#9139
anti-phospho-STAT3 (Y705) polyclonal rabbit	Cell Signaling Technology	Cat#9131
anti-phospho-STAT3 (Y705) polyclonal rabbit	Cell Signaling Technology	Cat#9134
anti-pY (4G10)	Merck Millipore	Cat#05-321
anti-ERK1/2 monoclonal rabbit	Cell Signaling Technology	Cat#4695
anti-phospho-ERK1/2 (T202/Y204) monoclonal rabbit	Cell Signaling Technology	Cat#4370
anti-STAT1	Cell Signaling Technology	Cat#9172
anti-Kap-1/TRIM28 rabbit polyclonal	Merck Millipore	Cat#ABE1859
anti-EGFL8 rabbit polyclonal	Abcam	Cat#ab58650
anti-SOCS1	Cell Signaling Technology	Cat#3950
anti-B2M	Siddle et al., 2013	N/A
anti-phospho-STAT1 (Y701) (58D6) monoclonal rabbit	Cell Signaling Technology	Cat#9167
Ki67 (NCL-Ki67p)	Leica Biosystems	Cat#KI67-MM1-L-CE
Cleaved Caspase 3 (Asp175)	Cell Signaling Technology	Cat#9661S
ECL anti rabbit IgG (NA934V)	GE Healthcare	Cat#NA934
anti-mouse (NA931) HRP	GE Healthcare	Cat#NA931
anti-MMP2 polyclonal rabbit	Abcam	Cat#ab37150
Biological Samples		
Devil facial tumor disease 1 (DFT1) biopsy: 08/0038, Mid Tumour T1	This paper	N/A
Devil facial tumor disease 1 (DFT1) biopsy: 08/0195, Mid Tumour T2	This paper	N/A
Tasmanian devil spleen biopsy: 08/0195, Spleen	This paper	N/A
Tasmanian devil skin biopsy: 08/0195, Skin	This paper	N/A
Devil facial tumor disease 1 (DFT1) biopsy: 08/0289, Early Tumour T2	This paper	N/A
Devil facial tumor disease 1 (DFT1) biopsy: 08/0289, Mid Tumour T1	This paper	N/A
Tasmanian devil spleen biopsy: 08/0289, Spleen	This paper	N/A
Tasmanian devil skin biopsy: 08/0289, Skin	This paper	N/A
Devil facial tumor disease 1 (DFT1) biopsy: 08/0590, Mid Tumour T2	This paper	N/A
Devil facial tumor disease 1 (DFT1) biopsy: 08/0590, Late Tumour T1	This paper	N/A

(*Continued on next page*)

Continued

REAGENT or RESOURCE	SOURCE	IDENTIFIER
Tasmanian devil spleen biopsy: 08/0590, Spleen	This paper	N/A
Tasmanian devil skin biopsy: 08/0590, Skin	This paper	N/A
Devil facial tumor disease 1 (DFT1) biopsy: 08/1818, Early Tumour T3	This paper	N/A
Devil facial tumor disease 1 (DFT1) biopsy: 08/1818, Mid Tumour T2	This paper	N/A
Devil facial tumor disease 1 (DFT1) biopsy: 08/1818, Late Tumour T1	This paper	N/A
Tasmanian devil spleen biopsy: 08/1818, Spleen	This paper	N/A
Tasmanian devil skin biopsy: 08/1818, Skin	This paper	N/A
Tasmanian devil nerve biopsy: Christine	This paper	N/A
Tasmanian devil nerve biopsy: Curley Shirley	This paper	N/A
Chemicals, Peptides, and Recombinant Proteins		
Recombinant devil interferon gamma (rIFNγ)	Siddle et al., 2013	N/A
Kinase Inhibitor Drug Library	TargetMol and Cayman Chemical	Cat# L1600 and Cat# 10505 respectively
2K Drug Library	Sdelci et al., 2016	N/A
Sapitinib	Adooq Bioscience	Cat#A10116
Lapatinib	Adooq Bioscience	Cat#A10514
PG-S3-009	Garg et al., 2017	N/A
DR-1-55	This paper	N/A
Deposited Data		
Proteomic Data	This paper	PRoteomics IDEentification database accession number 1-20180126-165173
DNA Methylation Data	This paper	Gene Expression Omnibus database accession number GSE108160
RNA Seq Data	This paper	Gene Expression Omnibus database accession number GSE108107
Experimental Models: Cell Lines		
Devil facial tumor disease 1 (DFT1) cell line: 06/2887, Strain 1	Deakin et al., 2012	N/A
Devil facial tumor disease 1 (DFT1) cell line: 05/2569, Strain 2	Deakin et al., 2012	N/A
Devil facial tumor disease 1 (DFT1) cell line: 06/0368, Strain 3	This paper	N/A
Devil facial tumor disease 1 (DFT1) cell line: 07/0192, Strain 4	Ingles and Deakin, 2015	N/A
Tasmanian devil fibroblast cell line	Murchison et al., 2012	N/A
Experimental Models: Organisms/Strains		
NOD scid gamma (NSG) mice	The Jackson Laboratory	Cat#005557
Oligonucleotides		
RPL13A forward: 5’-CCCCACAAGACCAAGCGAGGC-3’	Siddle et al., 2013	N/A
RPL13A reverse: 5’-ACAGCCTGGTATTTCCAGCCAACC-3’	Siddle et al., 2013	N/A
EGFR forward: 5’-GCAGATAGCCAAGGGTATGAGTTACC-3’	This paper	N/A
EGFR reverse: 5’-TTTTGCCAGCCCAAAATCTGT-3’	This paper	N/A
ERBB2 forward: 5’-GGAACCCAAGTGTGCACAGG-3’	This paper	N/A
ERBB2 reverse: 5’-TGGCATCAGCAGGCAGGTA-3’	This paper	N/A
ERBB3 forward: 5’-TACATGGTCATGGTTAAGTGCTGG-3’	This paper	N/A
ERBB3 reverse: 5’-GGTGGATCTCGGGCCATT-3’	This paper	N/A
MHC-1 (SAHA-UC; KY194695) forward: 5’-CCGTGGGC TACGTGGACGATCAGC-3’	Siddle et al., 2013	N/A
MHC-1 (SAHA-UC; KY194695) reverse: 5’-GTCGTAGG CGAACTGAAG-3’	Siddle et al., 2013	N/A
B2M forward: 5’-TGTGCATCCTTCCCTACCTGGAGG-3’	Siddle et al., 2013	N/A

(*Continued on next page*)

Continued		
REAGENT or RESOURCE	SOURCE	IDENTIFIER
B2M reverse: 5'-CATTGTTGAAAGACAGATCGGACCGC-3'	Siddle et al., 2013	N/A
STAT1 forward: 5'-GGAAAAGCAAGACTGGGACTATGC-3'	This paper	N/A
STAT1 reverse: 5'-GCGGCTATAGTGCTCATCCAA-3'	This paper	N/A
STAT3 forward: 5'-GGAAGCTGACCCAGGTAGTGC-3'	This paper	N/A
STAT3 reverse: 5'-CGGCAGGTCAATGGTATTGC-3'	This paper	N/A
TRIM28 forward: 5'-AAGTGCGCACCTCCATCC-3'	This paper	N/A
TRIM28 reverse: 5'-CCCGCTTGTTGAGCTCCTT-3'	This paper	N/A
EGF forward: 5'-TATGACTGGTACCGGCCCTG-3'	This paper	N/A
EGF reverse: 5'-TGCCAGCATTAGCTACCACTTGT-3'	This paper	N/A
NRG1 forward: 5'-CAGATACTCGTGCAAGTGCCC-3'	This paper	N/A
NRG1 reverse: 5'-TGCAGATGCCAGTGATGGTC-3'	This paper	N/A
EGFL8 forward: 5'-TCCATACAGCAAGGGAGTTTGC-3'	This paper	N/A
EGFL8 reverse: 5'-ATCCGCTGACCTGCACACA-3'	This paper	N/A
HBEGF forward: 5'-GGCTGCTCATGTTCAGGTACC-3'	This paper	N/A
HBEGF reverse: 5'-TTTCCATCAGTGGGCAATAGG-3'	This paper	N/A
MMP2 forward: 5′-CAGACAAAGAGTTGGCTGTACAATACC-3′	This paper	N/A
MMP2 reverse: 5′-CCTTCAGCACAAACAGGTTGC-3′	This paper	N/A

CONTACT FOR REAGENT AND RESOURCE SHARING

Further information and requests for resources and reagents should be directed to and will be fulfilled by the Lead Contact, Andreas Bergthaler (abergthaler@cemm.oeaw.ac.at).

EXPERIMENTAL MODEL AND SUBJECT DETAILS

Cell Culture and Tissue Biopsies

DFTD cells were grown from primary cell cultures derived from fine needle aspirates that have been collected from the wild (see Table S1) (Deakin et al., 2012; Ingles and Deakin, 2015; Murchison et al., 2012). Devil Facial Tumor cell lines T1-4 were grown in RPMI (Gibco 21875-034) supplemented with 10% Fetal bovine serum (PAA A15-101), 1% Pen Strep Glutamine (Gibco 10378-016) and 50 μM 2-Mercaptoethanol (Sigma M-3148). Fibroblasts were grown in Advanced DMEM (Gibco 12491-015) supplemented with 10% Fetal bovine serum (PAA A15-101), 1% Pen Strep Glutamine (Gibco 10378-016). Cells were grown at 35°C in 5% CO_2 and lifted with either PBS containing 1 mM EDTA or 0.05% Trypsin-EDTA (Gibco 25300-054). CHO supernatants containing recombinant devil interferon gamma (rIFNγ) was used 1:3 diluted (Siddle et al., 2013). Control cells received supernatants of wild type CHO cells. The ERBB inhibitors sapitinib (Adooq Bioscience, Cat# A10116) and lapatinib (Adooq Bioscience, Cat# A10514) were dissolved in DMSO and used at the indicated concentrations. The STAT3 inhibitors PG-S3-009 and DR-1-55 were dissolved in DMSO and used at the indicated concentrations.

Primary biopsies from Tasmanian devils were obtained from the Department of Primary Industries, Parks, Water and Environment (DPIPWE) (Tasmanian Government, Australia). All animal procedures were performed under a Standard Operating Procedure approved by the Secretary, Wildlife Management Branch, Tasmanian Government Department of Primary Industries, Parks, Water and the Environment (DPIPWE), in agreement with the DPIPWE Animal Ethics Committee. Biopsies were received frozen on dry ice and stored in liquid nitrogen until processed or embedded in paraffin blocks (Table S1).

Mouse Xenograft Studies

NOD scid gamma (NSG) mice (JAX # 005557) were maintained under pathogen-free conditions at the University of Veterinary Medicine, Vienna. Mice were at the age of 8-14 weeks at the time of cell implantation. Mice were kept at 12 hr/12 hr light cycle and received standard food and water *ad libidum*. All animal experiments were approved by the institutional ethics and animal welfare committee of the University of Veterinary Medicine, Vienna and the national authority according to §§ 26ff. of Animal Experiments Act, TVG 2012 BMWFW-68.205/0130-WF/V/3b/2016. The experimental design and number of mice assigned to each treatment arm were based on prior experience with similar models and provided sufficient statistical power to discern significant differences.

For sapitinib treatment mice were matched according to initial tumor size and randomized to treatment with sapitinib or vehicle (ddH_2O + 1% Tween80). No mice were excluded from the analysis. Mice were implanted subcutaneously in both flanks with $1x10^6$ Devil Facial Tumor cell line 1 (Table S1) in 100 μl PBS, using a 27G needle. Tumor growth was measured every second day using Vernier calipers for the duration of the experiment and tumor volumes were calculated with the following formula: tumor

volume = (length × width^2)/2. Treatment was initiated when the average tumor volume reached approximately 3 mm^3 and experiments were terminated once tumor volume in control group reached 1 cm^3. 1 out of 10 transplanted tumors did not grow in each group of mice (n=5). Tumors were resected and used for analysis of tumor weight, immunohistochemistry, real-time PCR and immunoblotting as described.

In the case of DR-1-55 treatment, the mice were randomly divided into two groups and, once the tumor volume reached approximately 3 mm^3, the mice were treated each second day with DR-1-55 (10 mg/kg) or vehicle (1% DMSO, 5% Tween80, 50% PEG400 in PBS) until tumors in control group reached approximately 1 cm^3.

METHODS DETAILS

Drug Viability Screen

We used a combined library of selected 1847 drugs (Sdelci et al., 2016) and 684 kinase inhibitors (Targetmol catalog no. L1600 and Cayman Chemical item no 10505), which were transferred onto 384-well plates using an acoustic liquid handler (Echo, Labcyte). 5000 cells per well of DFTD cell lines T1-4, and 2500 cells per well of fibroblasts were added on top of the drugs (50 nl in DMSO) with a dispenser (Thermo Fisher Scientific) to a total of 50 μl/well and incubated at 37°C. Cell viability was measured after 72 hr using the CellTiter-Glo® Luminescent Cell Viability Assay (Promega G7573) in a multilabel plate reader (EnVision, PerkinElmer). Initially, all drugs were tested on DFTD cell line #1 at a single dose (typically 10 μM). 434 drug hits with effects on cell viability were subsequently tested in a 4-dose response in triplicate wells of DFTD cell lines 1 to 4 as well as fibroblasts. Drug candidates were selected based on the difference between the Area Under the Curve (AUC) of each tumor cell line to the control fibroblast higher than 50. The addition of 2 standard deviations of the mean AUC of each strain should also result in a lower value than the subtraction of two standard deviations from the fibroblast AUC (Equation 1). This yielded 69 candidates that killed at least one DFTD cell line. Out of those, 41 drug candidates killed at least three out of four tumor cell lines but not fibroblasts. These 41 drugs were re-tested as 8-point dose-response in triplicates on the aforementioned five cell lines and cell viability was assessed by CellTiter-Glo as described previously. The STAT3 inhibitors PG-S3-009 (Garg et al., 2017) and DR-1-55 were tested separately as 5-point dose-response curves in triplicates. The percentage of control (POC) was calculated by using linear regression for each plate individually, setting the mean signal of the negative control wells (DMSO) to 100% and the mean signal of the positive control wells to 0%.

$$\overline{AUC_{fibroblast}} - 2Sd(AUC_{fibroblast}) > \overline{AUC_{tumor\ strains}} - 2Sd(AUC_{tumor\ strains}) \quad \text{(Equation 1)}$$

Equation 1: Equation identifying significant drugs killing the DFTD tumor strains compared to the fibroblast. AUC stands for area under the curve. Sd stands for standard deviation.

DNA and RNA Isolation

For DNA methylation analysis, approximately 20 mg of primary Tasmanian devil tissue was isolated and homogenised using the Tissue Lyser II (Qiagen, Hilden, Germany, 12 x 30 sec, 30 Hz). DNA and RNA were isolated using the AllPrep DNA/RNA Mini Kit (Qiagen, 80204), according to the manufacturer's instructions. For expression analysis from cell lines, total RNA was isolated from approximately 1x10^6 cells using QIAzol lysis reagent according to the manufacturer's instructions (Qiagen).

Real-Time PCR

Isolated RNA was reverse transcribed into cDNA using the First Strand cDNA Synthesis Kit (Fermentas) according to the manufacturer's instructions. Subsequent gene expression was then analysed using SYBR Select Master Mix (Applied Biosystems; 4472908). We designed and used the following gene-specific primers: 5'- CCCCACAAGACCAAGCGAGGC -3' and 5'- ACAGCCTGGTATTTC CAGCCAACC -3' for *RPL13A* (Siddle et al., 2013), 5'- GCAGATAGCCAAGGGTATGAGTTACC-3' and 5'- TTTTGCCAGCCCA AAATCTGT-3' for *EGFR*, 5'- GGAACCCAAGTGTGCACAGG-3' and 5'- TGGCATCAGCAGGCAGGTA-3' for *ERBB2*, and 5'- TACATG GTCATGGTTAAGTGCTGG-3' and 5'- GGTGGATCTCGGGCCATT-3' for *ERBB3*, 5'- CCGTGGGCTACGTGGACGATCAGC -3' and 5'- GTCGTAGGCGAACTGAAG -3' for *MHC-1* (*SAHA*-UC; KY194695) (Siddle et al., 2013), 5'-TGTGCATCCTTCCCTACCTGGAGG -3' and 5'-CATTGTTGAAAGACAGATCGGACCGC -3' for *B2M* (Siddle et al., 2013), 5'- GGAAAAGCAAGACTGGGACTATGC -3' and 5'- GCGGCTATAGTGCTCATCCAA -3' for *STAT1*, 5'- GGAAGCTGACCCAGGTAGTGC -3' and 5'- CGGCAGGTCAATGGTATTGC -3' for *STAT3,* 5'-AAGTGCGCACCTCCATCC-3' and 5'-CCCGCTTGTTGAGCTCCTT-3' for *TRIM28*, 5′-TATGACTGGTACCGGCC CTG-3′ and 5′-TGCCAGCATTAGCTACCACTTGT-3′for *EGF*, 5′-CAGATACTCGTGCAAGTGCCC-3′ and 5′-TGCAGATGCCAGT GATGGTC-3′for *NRG1*, 5′-TCCATACAGCAAGGGAGTTTGC-3′ and 5′-ATCCGCTGACCTGCACACA-3′for *EGFL8,* 5′-GGCTGCTC ATGTTCAGGTACC-3′ and 5′-TTTCCATCAGTGGGCAATAGG-3′ for *HBEGF,* 5′-CAGACAAAGAGTTGGCTGTACAATACC-3′ and 5′-CCTTCAGCACAAACAGGTTGC-3′ for *MMP2.* Designed forward primers span exon-exon junctions where possible.

Western Blotting

Approximately 5x10^6 cells from DFTD strains 1-4 and 2.5x10^6 Tasmanian devil fibroblasts were pelleted (260 *g*, 5 min, 4°C; Table S1), washed three times in cold PBS, snap frozen in liquid nitrogen and frozen at -80°C until processed. Sample preparation and Western blotting was performed using standard techniques. Nitrocellulose membranes (0.45 μm Amersham Protran 10600002, GE Healthcare, Buckinghamshire, UK) were incubated with the following antibodies in the dilution as indicated (see also Table S1): specific

anti-phospho-STAT3 (Y705) polyclonal rabbit (1:1000; 9131; Cell Signaling Technology, Cambridge, UK), anti-STAT3 monoclonal mouse (1:1000; 610189; BD Biosciences, Franklin Lakes, NJ, USA) or (9139; Cell Signaling; 1:1000), anti-phospho-STAT3 (S727) polyclonal rabbit (1:1000; 9134; Cell Signaling Technology, Cambridge, UK), anti-STAT1 (rabbit; 9172; Cell Signaling; 1:1000), anti-phospho-STAT1 (Y701) monoclonal rabbit (1:1000; 9167; Cell Signaling Technology), anti-EGFL8 polyclonal rabbit (1:1000; ab58650; Abcam, Cambridge, UK), anti-phospho-EGFR (Y1068) monoclonal rabbit (1:1000; 3777; Cell Signaling Technology), anti-EGFR monoclonal rabbit (1:1000; sc-373746; Santa Cruz, Dallas, TX, USA), anti-phospho-HER2/ERBB2 (Y1221/1222) monoclonal rabbit (1:1000; 2243; Cell Signaling Technology), anti-ERBB2 monoclonal mouse (1:1000, sc-7301; Santa Cruz Biotechnology), anti-phospho-HER3/ERBB3 (Y1289) monoclonal rabbit (1:1000; 2842; Cell Signaling Technology), anti-HER3/ERBB3 monoclonal rabbit (1:1000; 12708; Cell Signaling Technology), anti-HSC70 monoclonal mouse (1:1000; sc-7298; Santa Cruz), anti-TRIM28 polyclonal rabbit (1:1000; ABE1859; Millipore); anti-ERK1/2 monoclonal rabbit (1:1000; 4695; Cell Signaling Technology, Cambridge, UK), anti-phospho-ERK1/2 (T202/Y204) monoclonal rabbit (1:1000; 4370; Cell Signaling Technology, Cambridge, UK), anti-pY (4G10; Merck Millipore; 1:1000), anti-SOCS (1:1000; 3950; Cell Signaling Technology, Cambridge, UK), anti-B2M (1:1000; (Siddle et al., 2013)), ECL anti-rabbit IgG (NA934V) or anti-mouse (NA931) HRP (1:10000; GE Healthcare, Buckinghamshire, UK).

Immunoprecipitation

Cells were lysed in HE buffer (10 mM HEPES (pH 7.35), 1 mM EDTA) supplemented with protease inhibitors using a dounce tissue grinder. Human HT-29 cells (ATCC HTB-38) served as control. For immunoprecipitation, 1 mg protein lysate was incubated with 2 μg of anti-STAT3 (9139; Cell Signaling) or anti-STAT1 (9172; Cell Signaling) at 4°C overnight and immunoprecipitated with 25 μl Dynabeads Protein G (10004D; Thermo Fisher Scientific, Waltham, MA, USA) for 2 hr at 4°C. Beads were washed 3x with HE buffer and samples were eluated in 40 μl Laemmli buffer at 95°C for 10 min.

Histology

DFTD tissues of diseased animals (328T1 and 463T1) were fixed in 10% neutral buffered formalin and paraffin-embedded. 2 μm FFPE consecutive tumor sections were stained with Hematoxylin (Merck, Darmstadt, Germany) and Eosin G (Carl Roth). For immunohistochemical stainings, heat-mediated antigen retrieval was performed in citrate buffer at pH 6.0 (S1699; Dako, Agilent, Santa Clara, CA, USA), EDTA at pH 8.0 or TE at pH 9.0. Sections were stained with antibodies specific to STAT3 (1:200; pH 6; 9139; Cell Signaling Technology), phospho-STAT3 (S727) monoclonal rabbit (1:80; pH 9; 9134; Cell Signaling Technology); EGFR monoclonal mouse (1:300; 610016; pH 9; BD Biosciences), HER2/ERBB2 monoclonal rabbit (1:200, 4290; Cell Signaling Technology), HER3/ERBB3 monoclonal rabbit (1:200, 12708; Cell Signaling Technology), Periaxin/PRX (1:200, HPA001868, Sigma Aldrich), MMP2 (1:200; ab37150, Abcam), Ki67 (1:1000; NCL-Ki67p; Novocastra, Leica Biosystems;) or Cleaved Caspase 3 (Asp175) (1:200, 9661S, Cell Signaling Technology) using standard protocols (see also Table S1). Images were photographed using an Olympus BX 53 microscope, and were quantified using HistoQuest TM software (TissueGnostics GesmbH, Vienna, Austria).

Immunofluorescence

Cells were grown on sterile glass coverslips, rinsed with PBS, fixed in 4% paraformaldehyde for 10 min and permeabilized using 0.5% Triton X-100 in PBS for 8 min. Cells were blocked in 3% BSA+0.1% Triton in PBS for 1 hr, incubated with primary antibodies (anti-STAT1 1:500, 9172 Cell Signaling Technology; anti-STAT3 1:1500, 9139 Cell Signaling Technology) for 1 hr or at 4°C overnight, washed and probed with the secondary antibodies conjugated to Alexa Fluor 488 and Alexa Fluor 568 (Molecular Probes) for 1 hr. Cells were stained with DAPI and mounted in VECTASHIELD Antifade Mounting Medium with DAPI (Vector Laboratories). The images were acquired with a Zeiss LSM 880 Confocal Laser Scanning Microscope.

Mass-Spectrometry Based Proteomics

Sample Preparation for MS

Approximately 1x10^7 cells from DFT1 tumor cell line T1 were pelleted (260*g*, 5 min, 4°C), washed three times in PBS and frozen at -80°C until processed. Primary biopsies were thawed and placed in a petri dish. Using a scalpel blade, 5×5 to 5×8 mm pieces of tissue were excised from the solid mass and placed in a 2 ml Eppendorf tube. Depending on the size of the piece of tissue, 500-1000 μl of lysis buffer (50 mM HEPES, pH 8.0, 2% SDS, 1 mM PMSF, and protease inhibitor cocktail (Sigma-Aldrich)) was added to the tumour and skin samples. Samples were homogenised using the Tissue Lyser II (Qiagen, Hilden, Germany) for 4×2 min, 30 Hz. For some samples, it was necessary to repeat the homogenization procedure. Spleen samples were pre-cleared of blood before tissue lysis. Tissue pieces were placed in a 2 ml Eppendorf tube containing 1 ml red blood cell (RBC)-lysis buffer (eBioscience, San Diego, USA). Spleen tissue was homogenised using the Tissue Lyser II (Qiagen, Hilden, Germany) for 3×30s, centrifuged at 20000 *g* for 10 min and supernatant containing the lysed red blood cells removed. Lysis was performed at room temperature (RT) for 20 min. Lysed samples were heated at 99°C for 5 min and then cooled to RT. The cell lysate was sonicated using a Covaris S2 high performance ultrasonicator (Covaris Inc., Brighton, UK). The lysate was centrifuged at 20000 *g* for 15 min at 20°C, and the protein extract was collected from the supernatant. Total protein content of the whole tissue lysates was determined using the BCA protein assay kit (Pierce Biotechnology, Rockford, IL) following the recommendations of the manufacturer. The assay was performed in a 96-well plate using 10 μl of each lysate and standard protein. The samples were measured in triplicates. Bovine serum albumin (BSA) (Pierce Biotechnology, Rockford, IL) was used as the standard protein.

Filter-Aided Sample Preparation (FASP)

100 μg total protein per tissue was used for FASP digestion. Dithiothreitol (DTT; SIGMA-Aldrich Chemie, Vienna, Austria) was added to sample to a final concentration of approx. 83 mM. After incubation of the samples at 99°C for 5 min, FASP digestion was performed using a 30 kDa molecular weight cut-off filter (Microcon-30, Ultracel YM-30, Merck-Millipore Co., Cork, IRL) (Wisniewski et al., 2009). Briefly, 200 μL 8 M urea in 100 mM Tris-HCl (pH 8.5) (UA) was added to the samples. If the volume exceeded 50 μl, then 400 μl UA was added. After equilibration of the filter units with 200 μl UA and centrifugation at 14000×*g* for 15 min, the lysed samples were applied in steps of 250 μl to the filter unit and centrifuged at 14000 *g* for 15 min at 20°C to remove SDS. Any remaining SDS was exchanged by urea in a second washing step with 200 μl UA. The proteins were alkylated with 100 μl 50 mM iodoacetamide (Sigma-Aldrich Chemie, Vienna, Austria) for 30 min at RT. Afterwards, three washing steps with 100 μL UA solution were performed, followed by three washing steps with 100 μL 50 mM TEAB buffer (Sigma-Aldrich, Vienna, Austria). Proteins were digested with Trypsin overnight at 37°C. Peptides were recovered using 40 μl 50 mM TEAB buffer followed by 50 μl of 0.5 M NaCl (Sigma-Aldrich, Vienna, Austria).

Two-dimensional liquid chromatography was performed by reverse-phase chromatography at high and low pH. FASP digests were purified by solid-phase extraction (SPE) (MacroSpin Columns, 30-300 μg capacity, Nest Group Inc. Southboro, MA, USA) and reconstituted in 23 μl 5% acetonitrile, 10 mM ammonium formate. Peptides were separated on a Gemini-NX C18 (150 × 2 mm, 3 μm, 110 Å, Phenomenex, Torrance, US) using a 30 min gradient from 5 to 90% acetonitrile containing 10 mM ammonium formate buffer, pH 10, at a flow rate of 100 μl/min, using an Agilent 1200 HPLC system (Agilent Biotechnologies, Palo Alto, CA). Details of the methodology are as described previously (Bennett et al., 2011). Ten time-based fractions were collected. Samples were acidified by the addition of 5μl 5% formic acid. Solvent was removed in a vacuum concentrator, and samples were reconstituted in 5% formic acid. Liquid chromatography mass spectrometry was performed on a hybrid linear trap quadrupole (LTQ) Orbitrap Velos mass spectrometer (ThermoFisher Scientific, Waltham, MA) using the *Xcalibur version 2.1.0* coupled to an Agilent 1200 HPLC nanoflow system (dual pump system with one trap-column and one analytical column) via a nanoelectrospray ion source using liquid junction (Proxeon, Odense, Denmark). Solvents for HPLC separation of peptides were as follows: solvent A consisted of 0.4% formic acid (FA) in water, and solvent B consisted of 0.4% FA in 70% methanol and 20% 2-propanol. From a thermostatted microautosampler, 8 μl of the tryptic peptide mixture were automatically loaded onto a trap column (Zorbax 300SB-C18 5 μm, 5×0.3 mm, Agilent Biotechnologies) with a binary pump at a flow rate of 45 μl/min. 0.1% trifluoroacetic acid (TFA) was used for loading and washing the precolumn. After washing, the peptides were eluted by back-flushing onto a 16 cm fused silica analytical column with an inner diameter of 50 μm packed with C18 reversed phase material (ReproSil-Pur120 C18-AQ, 3 μm, Dr. Maisch GmbH, Ammerbuch-Entringen, Germany). The peptides were eluted from the analytical column with a 27 min gradient ranging from 3% to 30% solvent B, followed by a 25 min gradient from 30% to 70% solvent B, and finally a 7 min gradient from 70% to 100% solvent B at a constant flow rate of 100 nl/min (Bennett et al., 2011). The analyses were performed in a data-dependent acquisition mode, and dynamic exclusion for selected ions was 60s. A top 15 collision-induced dissociation (CID) method was used, and a single lock mass at *m/z* 445.120024 ($Si(CH_3)_2O)_6$) was employed (Olsen et al., 2005). Maximal ion accumulation time allowed in CID mode was 50 ms for MSn in the LTQ and 500 ms in the C-trap. Automatic gain control (AGC) was used to prevent overfilling of the ion traps and was set to 5000 in MS^2 mode for the LTQ and 10^6 ions for a full MS^1 FTMS scan. Intact peptides were detected in the Orbitrap Velos at a resolution of 60000 resolution (at *m/z* 400). The threshold for switching from MS^1 to MS^2 was 2000 counts.

DNA Methylation Analysis

DNA methylation profiling by RRBS was performed as described previously using 100 ng of genomic DNA isolated from RNA-later preserved tissue samples through the Allprep DNA/RNA Mini kit (QIAGEN) (Klughammer et al., 2015). Methylated and unmethylated spike-in controls were added in a concentration of 0.1% to assess bisulfite conversion efficiency independent of CpG context. DNA was digested using the restriction enzymes MspI and TaqI in combination (as opposed to only MspI in the original protocol) in order to increase genome-wide coverage. Restriction enzyme digestion was followed by fragment end repair, A-tailing, and adapter ligation. The amount of effective library was determined by qPCR, and samples were multiplexed in pools of 13 with similar qPCR C_t values. The pools were then subjected to bisulfite conversion followed by library enrichment by PCR. Enrichment cycles were determined using qPCR and ranged from 9 to 13 (median: 11). After confirming adequate fragment size distributions on Bioanalyzer High Sensitivity DNA chips (Agilent), libraries were sequenced on Illumina HiSeq 3000/4000 machines in a 50 bp single-read setup.

Transcriptome Expression Analysis and Variant Calling

The amount of total RNA was quantified using Qubit 2.0 Fluorometric Quantitation system (Life Technologies) and the RNA integrity number (RIN) was determined using Experion Automated Electrophoresis System (Bio-Rad). RNA-seq libraries were prepared with TruSeq Stranded mRNA LT sample preparation kit (Illumina) using Sciclone and Zephyr liquid handling robotics (PerkinElmer). Library amount was quantified using Qubit 2.0 Fluorometric Quantitation system (Life Technologies) and the size distribution was assessed using Experion Automated Electrophoresis System (Bio-Rad). For sequencing 6 libraries were pooled, diluted and sequenced on Illumina HiSeq 3000/4000 using 75 bp paired-end chemistry. Base calls provided by the Illumina Realtime Analysis software were converted into BAM format using Illumina2bam and demultiplexed using BamIndexDecoder (https://github.com/wtsi-npg/illumina2bam).

Measurement of Biochemistry Parameters

Serum was prepared by centrifugation of the whole blood for 20 min at 7000 rpm. Serum concentration of alanine aminotransferase, aspartate aminotransferase and blood urea nitrogen was measured using a chemistry analyzer (IDEXX VetTest 8008, IDEXX GmbH, Ludwigsburg, Germany).

QUANTIFICATION AND STATISTICAL ANALYSIS

Immunohistochemistry and Immunofluorescence Quantification

Immunohistochemical images were taken with Olympus BX 53 microscope and the quantification was performed using HistoQuest TM software (TissueGnostics GesmbH, Vienna Austria). Quantification of immunofluorescence was carried out using a co-localization pipeline of CellProfiler (version 3.1.5). Briefly, illumination was corrected using the "Regular" function and "Fit polynomial" as smoothing method. Subsequently, images were aligned with the "Mutual information" option and objects were identified with the following settings: size (pixels) 3-15, Threshold strategy: global, Threshold method: Otsu, with three classes thresholding, pixels in the middle intensity class were assigned as background and objects touching the image boundary were discarded. Objects were classified as co-localized if they touched, overlapped or enclosed each other and as not co-localized when none of the above applied. Percentages were calculated on a per image basis and for each condition 10 images were used. Data shown is representative of three similar experiments. Displayed is the percentage of STAT1 objects that were colocalized with a STAT3 object.

Mass-Spectrometry Based Proteomics - Quantification

The data were analyzed using Proteome Discoverer version 2.2 against the Uniprot Tasmanian Devil fasta database version 2016.11 including isoforms obtained by VARSPLIC (Kersey et al., 2000) and appended with known contaminants (91064 sequences total). The precursor masses were first recalibrated using the recalibration node (parameters: precursor mass tolerance: 20 ppm, fragment mass tolerance 0.5 Da, Carbamidomethylation of cysteine as static modification). The recalibrated data were then searched using Sequest HT (Eng et al., 1994) and Mascot (v2.3.02, MatrixScience, London, U.K.) (Perkins et al., 1999) search engines with precursor mass tolerance being 4 ppm, fragment ion tolerance was 0.3 Da, methionine oxidation used as dynamic modification, and carbamidomethylation of cysteine as static modification in both search engines. Moreover protein N-terminal acetylation was considered as dynamic modification in Sequest HT. Minimum length of peptides was set to 6 and maximum number of missed cleavages was set to two in Sequest HT and to one in Mascot. Percolator (Kall et al., 2007) was used to filter peptide-spectra matches (PSMs) at 1% false discovery rate (FDR) and QVALITY (Kall et al., 2009) to filter identified peptides at 1% FDR. Identified proteins were also filtered at 1% FDR by considering sum of negative logarithm of posterior error probabilities of connected PSMs. Matches against reversed fasta database were used to estimate FDR at PSM, peptide, and protein level. Abundance of proteins was quantified using the Minora feature detection node and integrating area under the MS1 chromatogram.

Principal component analysis was performed on 3894 out of 6672 proteins quantified in all 19 samples. We further focused on 4981/6672 proteins quantified in at least 80% of the samples. The protein abundance data was normalized by variance stabilizing transformation (Huber et al., 2002). Missing values are imputed on normalized abundance values with the k Nearest Neighbors (kNN) algorithm implemented in the R Bioconductor package impute (Hastie et al., 2017).

For each protein with a missing abundance in any of the samples, the kNN algorithm identifies the set of 10 most similar proteins based on non-missing abundance values. Missing abundance is then imputed as an average abundance in that set. An average of the signal is then performed on the k closest neighbours. Differential analysis, tumor versus the healthy, was performed between biopsies (excluding the tumor cell line) using the limma Bioconductor package (Ritchie et al., 2015). Proteins were considered as differentially modulated if their adjusted p value was <= 0.05 and their absolute log2 Fold Change was >=1 between tumor and healthy biopsies. Hierarchical clustering of the 987 differentially modulated proteins was performed with Pearson's distance measure and the average clustering method.

DNA Methylation Analysis and Quantification

The DNA methylation (RRBS) data were analyzed using the RefFreeDMA pipeline as described previously to avoid potential biases in read mapping and methylation calling related to the scaffold assembly status of the published Tasmanian Devil reference genome sarHar1 (Klughammer et al., 2015). In brief, a custom *ad-hoc* reference genome was deduced directly from the RRBS sequencing reads and used for read mapping and methylation calling. Based on the thus produced DNA methylation profiles, differential DNA methylation analysis was performed as part of the RefFreeDMA pipeline and as originally described in (Assenov et al., 2014). Gene annotations were transferred by mapping the deduced genome fragments to the published scaffold-level Tasmanian Devil genome sarHar1, downloaded from the UCSC genome browser. A gene annotation file was produced by joining the UCSC transcript annotation file with the Ensembl v86 transcript annotation file of sarHar1 based on the common Ensembl transcript identifiers. Gene promoters were defined as the region between 5000 bases downstream and 2500 bases upstream of the transcription start site. 487540 (11.37%) of the individual CpGs are situated in promoters of annotated genes. For promoter methylation analysis we focused on a total of 69754 CpGs that are also covered in at least 80% of the samples. The significance of differential methylation throughout promoters or deduced genome fragments was assessed by combining the p values for single CpGs within the respective promoter regions or deduced genome fragments using an extension of the Fisher's method as described previously (Assenov et al., 2014; Klughammer et al., 2015).

Western Blot Quantification

Immunoblots were quantified using ImageJ (2.0.0) software. Three independent immunoblots were used for each quantification. Phospho-STAT3 levels were normalized to STAT3 and to loading control HSC70.

Transcriptome Expression Analysis and Variant Calling

Paired-end reads were trimmed for adaptor sequences and filtered with the trimmomatic tool (Bolger et al., 2014). Trimmed reads were aligned on the version 7.0 of the Tasmanian Devil with the STAR aligner (Dobin et al., 2013). Counting of reads on annotated transcripts (Sarcophilus_harrisii.DEVIL7.0.90.gtf from Ensembl) was performed with featureCounts (Liao et al., 2014). The DESeq2 Biconductor library has been used for counts normalization and differential analysis between the transcriptomes of the four DFTD and fibroblast cell lines (Love et al., 2014). Differentially expressed genes were identified based on the following cutoffs: an average minimum expression value between conditions of 50 reads, an absolute log fold change of 1, and an adjusted p value of maximum 0.05. The pipeline for variant calling is based on the GATK version 3.7, following the best-practices (Van der Auwera et al., 2013). Called variants were annotated with SNPEff v4.2 (Cingolani et al., 2012). Variants are filtered on strand bias (<30), quality (QD > 2), coverage (DP >= 10) and allele frequency (AF = 0.5).

Network Analysis

We performed Transcription Factor and Pathway Maps enrichment on the differentially modulated entities (proteins, genes) with the MetaCore™ (Thomson Reuters, version 6.32 build 69020; cut-off for p value of enrichment 0.05). The results are reported based on the z-score of enrichment for the transcription factors and as -log10 of the False Discovery Rate for the Pathway Maps. The 987 differentially modulated proteins were integrated together with the 166 genes with differentially modulated promoters and the candidates ERBB2 and ERBB3 from the drug-screen at the level of MetaCore interactions (Build Network functionality). Only high-confidence direct interactions between pairs of genes are considered: protein binding, transcription factor regulation, other functional interaction. 632 of the previous candidates form a direct network connection.

Motif Enrichment Analysis

We filtered individual CpG regions with less than 8 or more than 200 reads. On the remaining CpGs, we only kept those with less than 20% missing values across the 19 samples. Modulated individual CpGs between healthy and tumor samples were selected based on an adjusted p value smaller than 0.05, and a log fold-change greater than 1 for the UP dataset (4299 CpGs), and smaller than -1 for the DOWN dataset (1727 CpGs). Fasta sequences of these fragments were extracted. We performed motif enrichment analysis with the AME tool from the MEME package (McLeay and Bailey, 2010). We used the motif JASPAR CORE 2014 Vertebrate database (Mathelier et al., 2014). The motif enrichment for each fasta file accounts for background sequence composition by using the other sequence file as control.

Integrating DFTD Quantifications across Techniques

The DFTD tumor cell line T1 was the only sample quantified with all three techniques: RNA-seq, proteomics and methylation. We kept only the annotated (no SNO) genes from the RNA-seq data and computed the expression as the number of counts divided by the length of the gene as returned by featureCounts (Liao et al., 2014). The genes were ranked according to expression from the most to the least expressed. The top 15% most abundant proteins (817) were selected. We performed a Gene Set Enrichment Analysis (GSEA) (Mootha et al., 2003; Subramanian et al., 2005). Pre-ranked between the top DFTD 15% most abundant proteins and the ranked gene expression data using default parameters. For the annotated gene promoters with CpG fragments, we computed the average methylation level in the promoter regions defined as -5 kb to 2.5 kb around the TSS. Similarly, we ranked the genes according to their promoter methylation level. We selected the top 15% least methylated gene promoters (936) and performed GSEA Preranked on the ranked RNA-seq expression. Alternatively, we ranked the annotated genes detected in the DFTD cell line by both RNA-seq and methylation data with a random method for ties. We compared the ranks of methylation and transcriptomics using the hexbin plot in R (hexbin R package (version 1.27.2). A Spearman correlation is performed between the ranks of the two datasets.

To study potential effects on chemotaxis and cytoskeleton remodeling, we performed MetaCore Pathway Enrichments on all proteins that were modulated, be it UP or DOWN (abs(logFC) >= 1, adj.p.val <= 0.05; Table S5). Subsequently, we extracted proteins that were differentially modulated and led to the enrichments in the following pathways linked to chemotaxis and cytoskeleton remodeling: Cytoskeleton remodeling_Integrin outside-in signaling (4), Chemotaxis_SDF-1/CXCR4-induced chemotaxis of immune cells (5), Cytoskeleton remodeling,_Regulation of actin cytoskeleton organization by the kinase effectors of Rho GTPases (9) and Cell adhesion_Role of tetraspanins in the integrin-mediated cell adhesion (13). The respective proteins are reported in Table S5 the chemotaxisGenes.xlsx file together with their respective modulation in the Proteomics and RNAseq datasets. Next, we generated a heatmap based on the protein abundance values of these proteins across the biopsies, cell line, nerve, skin and spleen (clustering distance : pearson, clustering method: average). On the right genes that were found as being bound by STAT3 across any of the ENCODE Chip-seq datasets (Rouillard et al., 2016) were color-coded. In addition, we color-coded whether the genes were found and/or modulated in the RNA-seq data (DFTD tumor cell lines T1-T4 vs. fibroblasts).

Characterization of the STAT3 Inhibitor DR-1-55

To characterize drug-target binding, we performed a ^{19}F NMR protein-ligand experiment as done previously (Ali et al., 2016; Garg et al., 2017; Wingelhofer et al., 2018). Incubation of purified recombinant STAT3 with DR-1-55 results in line broadening of the ^{19}F peaks from the compound, indicating a longer correlation time for the drug, which is a consequence of protein-drug binding. Concomitantly, a release of fluoride ions is observed, the by-product of a covalent reaction of the protein and a pentafluorobenzene containing molecule. In addition, we performed a thermal shift assay and observed a dose-dependent decrease in the denaturation temperature of recombinant STAT3, indicative of covalent protein-ligand binding interaction (de Araujo et al., 2017).

DATA AND SOFTWARE AVAILABILITY

The data reported in this paper are tabulated in the Supplemental Information and archived at the following databases: proteomic data in the PRoteomics IDEentification (PRIDE) database with accession number (1-20180126-165173), DNA methylation data in the Gene Expression Omnibus (GEO) database with accession number (GSE108160) and RNA-seq data in the GEO database with accession number (GSE108107).

Cancer Cell, Volume *35*

Supplemental Information

The ERBB-STAT3 Axis Drives Tasmanian Devil Facial Tumor Disease

Lindsay Kosack, Bettina Wingelhofer, Alexandra Popa, Anna Orlova, Benedikt Agerer, Bojan Vilagos, Peter Majek, Katja Parapatics, Alexander Lercher, Anna Ringler, Johanna Klughammer, Mark Smyth, Kseniya Khamina, Hatoon Baazim, Elvin D. de Araujo, David A. Rosa, Jisung Park, Gary Tin, Siawash Ahmar, Patrick T. Gunning, Christoph Bock, Hannah V. Siddle, Gregory M. Woods, Stefan Kubicek, Elizabeth P. Murchison, Keiryn L. Bennett, Richard Moriggl, and Andreas Bergthaler

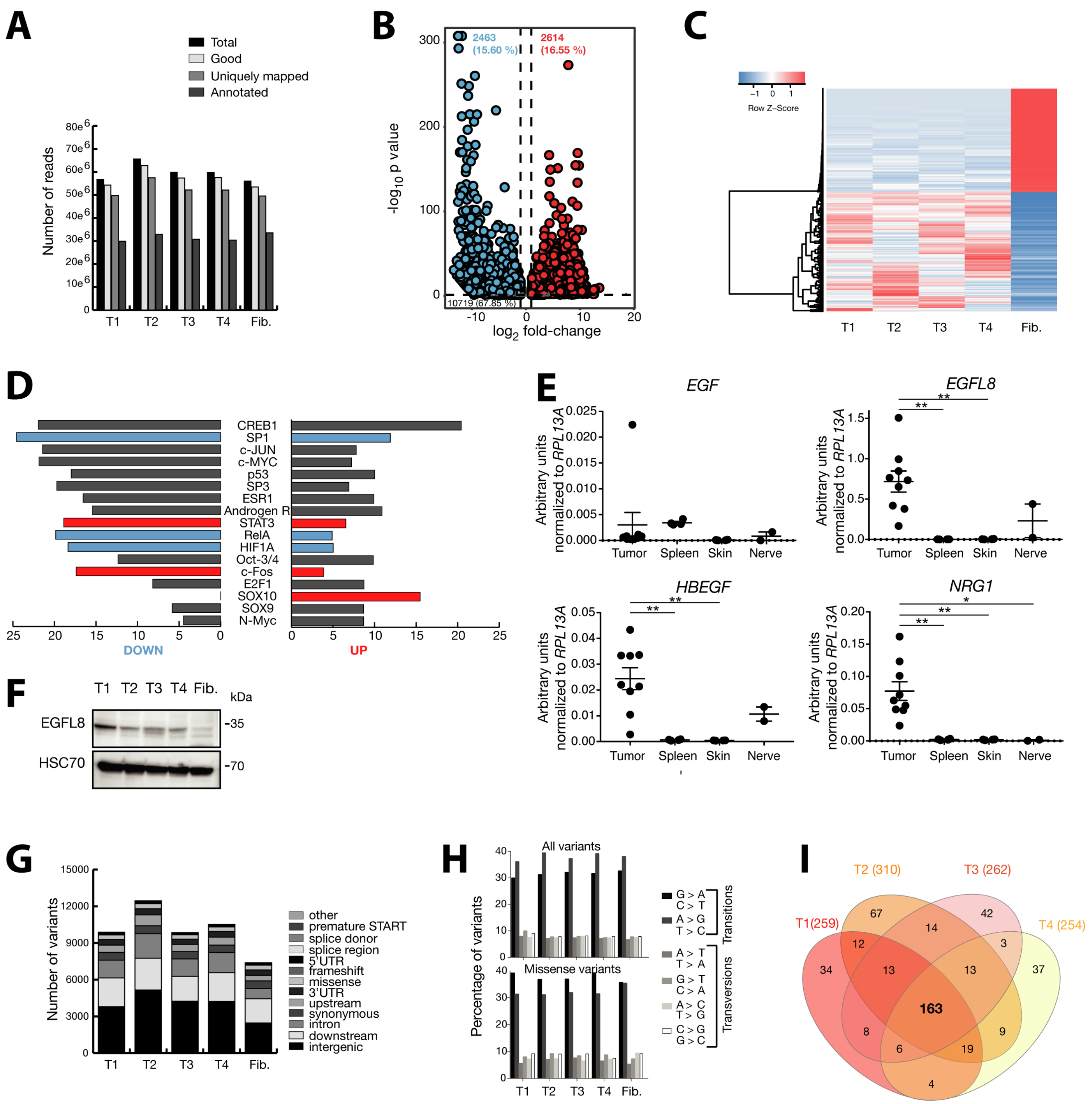
A
Total
Good
Uniquely mapped
Annotated
Number of reads
T1 T2 T3 T4 Fib.
B
2463 (15.60 %)
2614 (16.55 %)
10719 (67.85 %)
-log10 p value
log2 fold-change
C
Row Z-Score
T1 T2 T3 T4 Fib.
D
CREB1
SP1
c-JUN
c-MYC
p53
SP3
ESR1
Androgen R
STAT3
RelA
HIF1A
Oct-3/4
c-Fos
E2F1
SOX10
SOX9
N-Myc
DOWN
UP
E
EGF
EGFL8
HBEGF
NRG1
Arbitrary units normalized to RPL13A
Tumor Spleen Skin Nerve
F
T1 T2 T3 T4 Fib.
kDa
EGFL8
-35
HSC70
-70
G
Number of variants
other
premature START
splice donor
splice region
5'UTR
frameshift
missense
3'UTR
upstream
synonymous
intron
downstream
intergenic
H
All variants
Missense variants
Percentage of variants
G > A C > T
A > G T > C
Transitions
A > T T > A
G > T C > A
A > C T > G
C > G G > C
Transversions
I
T1(259)
T2 (310)
T3 (262)
T4 (254)
34
67
42
37
12
14
3
13
163
13
8
9
6
19
4

Figure S1, Related to Figure 1: RNA-seq and expression analysis

(**A**) Number of reads across four DFTD cell lines (T1-T4) and fibroblasts (Fib.). Total number of reads, reads passing the quality filters (good reads), reads mapping at unique positions on the genome and reads mapping on annotated genes are reported. (**B**) Volcano plot of the differentially expressed genes between the four DFTD and the fibroblast cell lines. 2463 genes are down-regulated (blue), and 2614 genes are up-regulated (red). (**C**) Heatmap of the 5077 differentially expressed genes between the DFTD and the fibroblast cell lines (expression ≥ 50 read counts, absolute log2 fold-change ≥ 1, and adjusted p value ≤ 0.05). (**D**) MetaCore Transcription Factor enrichment analysis. Enrichments analysis has been performed separately on the genes UP and DOWN modulated in the DFTD cells compared to fibroblasts. Transcription factors that are themselves modulated in our dataset are reported in bold and their bars colored in red or blue, for up and respectively down modulated. All transcription factors with a reported bar have significant enrichment p values (≤ 0.05). SOX10 that is highly expressed in the tumor cell lines and enriched exclusively for up-regulated genes is a transcription factor critical for Schwann cell development. Other enriched factors, like STAT3 are more abundant in the DFTD cell lines while, ESR1 is not expressed in the tumor cell lines. (**E**) *EGF, EGFL8, HBEGF* and *NRG1* were measured by real-time PCR from primary biopsies of DFTD tumors, spleen, skin and peripheral nerve. Statistical significance was calculated by One-way ANOVA. Scatter plots are shown with mean +/- SEM. (**F**) Western blot for EGFL8 from DFTD cell lines and Tasmanian devil fibroblasts. (**G**) Annotation classes of variant effects across the five cell lines. The vast majority of variants are localized in intergenic, downstream or intronic regions. (**H**) Transitions and transversions distribution of single nucleotide variants (SNVs) across samples. All the five samples are enriched for transitions across all or only missense SNVs. (**I**) Venn diagram of missense variants shared or specific for the tumor strains and absent from fibroblasts. 38% (163 out of 444 variants) are shared across the four strains.

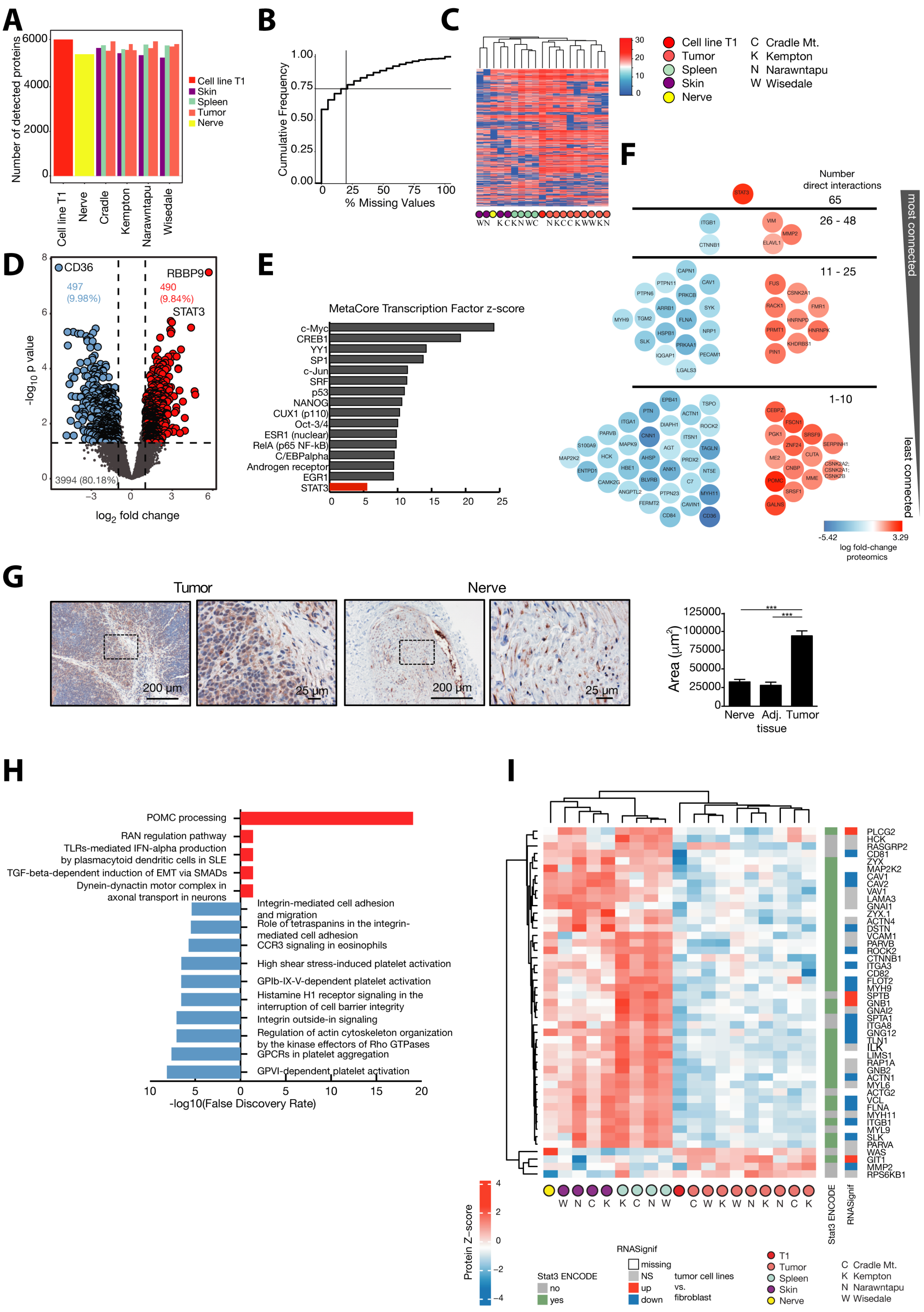
A
Number of detected proteins
6000
4000
2000
0
Cell line T1
Skin
Spleen
Tumor
Nerve
Cell line T1
Nerve
Cradle
Kempton
Narawntapu
Wisedale
B
Cumulative Frequency
1.00
0.75
0.50
0.25
0.00
0
25
50
75
100
% Missing Values
C
30
20
10
0
Cell line T1
Tumor
Spleen
Skin
Nerve
C Cradle Mt.
K Kempton
N Narawntapu
W Wisedale
D
CD36
RBBP9
497
(9.98%)
490
(9.84%)
STAT3
3994 (80.18%)
-log10 p value
log2 fold change
E
MetaCore Transcription Factor z-score
c-Myc
CREB1
YY1
SP1
c-Jun
SRF
p53
NANOG
CUX1 (p110)
Oct-3/4
ESR1 (nuclear)
RelA (p65 NF-kB)
C/EBPalpha
Androgen receptor
EGR1
STAT3
0
5
10
15
20
25
F
Number
direct interactions
65
26 - 48
11 - 25
1-10
most connected
least connected
-5.42
3.29
log fold-change
proteomics
G
Tumor
Nerve
200 μm
25 μm
200 μm
25 μm
Area (μm2)
125000
100000
75000
50000
25000
0
Nerve
Adj.
tissue
Tumor
H
POMC processing
RAN regulation pathway
TLRs-mediated IFN-alpha production
by plasmacytoid dendritic cells in SLE
TGF-beta-dependent induction of EMT via SMADs
Dynein-dynactin motor complex in
axonal transport in neurons
Integrin-mediated cell adhesion
and migration
Role of tetraspanins in the integrin-
mediated cell adhesion
CCR3 signaling in eosinophils
High shear stress-induced platelet activation
GPIb-IX-V-dependent platelet activation
Histamine H1 receptor signaling in the
interruption of cell barrier integrity
Integrin outside-in signaling
Regulation of actin cytoskeleton organization
by the kinase effectors of Rho GTPases
GPCRs in platelet aggregation
GPVI-dependent platelet activation
10
5
0
5
10
15
20
-log10(False Discovery Rate)
I
PLCG2
HCK
RASGRP2
CD81
ZYX
MAP2K2
CAV1
CAV2
VAV1
LAMA3
GNAI1
ZYX.1
ACTN4
DSTN
VCAM1
PARVB
ROCK2
CTNNB1
ITGA3
CD82
FLOT2
MYH9
SPTB
GNB1
GNAI2
SPTA1
ITGA8
GNG12
TLN1
ILK
LIMS1
RAP1A
GNB2
ACTN1
MYL6
ACTG2
VCL
FLNA
MYH11
ITGB1
MYL9
SLK
PARVA
WAS
GIT1
MMP2
RPS6KB1
Stat3 ENCODE
RNASignif
Protein Z-score
4
2
0
-2
-4
Stat3 ENCODE
no
yes
RNASignif
missing
NS
up
down
tumor cell lines
vs.
fibroblast
T1
Tumor
Spleen
Skin
Nerve
C Cradle Mt.
K Kempton
N Narawntapu
W Wisedale

Figure S2, Related to Figure 2: Proteomic analysis

(**A**) Number of quantified proteins for each sample. Individual Tasmanian devils are named according to their geographical sampling locations. “Cell line” denotes the DFTD cell line 06/2887 (labeled as T1 throughout the study) and “Nerve” stands for a healthy nerve biopsy (**Table S1**). (**B**) Cumulative frequency distribution of the percentage missing (non-quantified) protein abundance across the samples. We focus on proteins quantified in at least 80% of the samples (less than 20% missing values). This represents 4981/6672 proteins, almost 75% of the total identified proteins. (**C**) Hierarchical clustering of missing values (0-blue values) across samples. Missing values are not specific to one sample or condition but are distributed across all samples. (**D**) Volcano plot of the 4981 proteins on which we performed differential analysis. (**E**) Top 15 enriched transcription factors (TF) and STAT3 for the differentially modulated proteins: tumor versus healthy samples. The enrichment was performed with MetaCore™ (Thomson Reuters, version 6.32 build 69020) and the reported TF have all p values ≤ 0.05 and are ordered according to their z-score of enrichment. STAT3 is the most enriched TF that is itself differentially modulated in the proteomics dataset. (**F**) Proteins directly connected to and downstream of STAT3 (based on MetaCore database). Red proteins are more abundant in tumors compared to healthy biopsies, while blue stands for reduced protein in tumors. Proteins are ordered vertically according to how many connections they have inside the STAT3 downstream network, from the most connected (UP) to the least connected (DOWN). (**G**) Immunohistochemical stainings for MMP2 from tumor and peripheral nerve tissue biopsies. Shown images are consecutive images related to **Fig. 1E**. Dotted rectangle indicates magnified area. Quantification of MMP2 in tumor, adjacent tissue and peripheral nerve tissue. Scale bars, 200 μm and 25 μm. Statistical significance was calculated by unpaired t-test. Bars represent mean +/- SEM. (**H**) MetaCore Pathway Maps enrichments separately for UP (red) and DOWN-regulated (blue) proteins in tumor versus healthy tissues. Only the first ten pathways and with a false discovery rate ≤ 0.05 are reported. (**I**) Heatmap of the modulated proteins between tumor biopsies

versus the healthy tissue and involved in chemotaxis and cytoskeleton remodeling (46 proteins). The vast majority of these proteins (42/46) show down-regulation both in the tumor biopsies as well as the DFTD cell line. 34 out of these 46 genes have been shown to be bound by STAT3 across the ENCODE Chip datasets. The modulation status of the transcripts of each gene in the RNA-seq dataset tumor cell lines versus fibroblast is also reported.

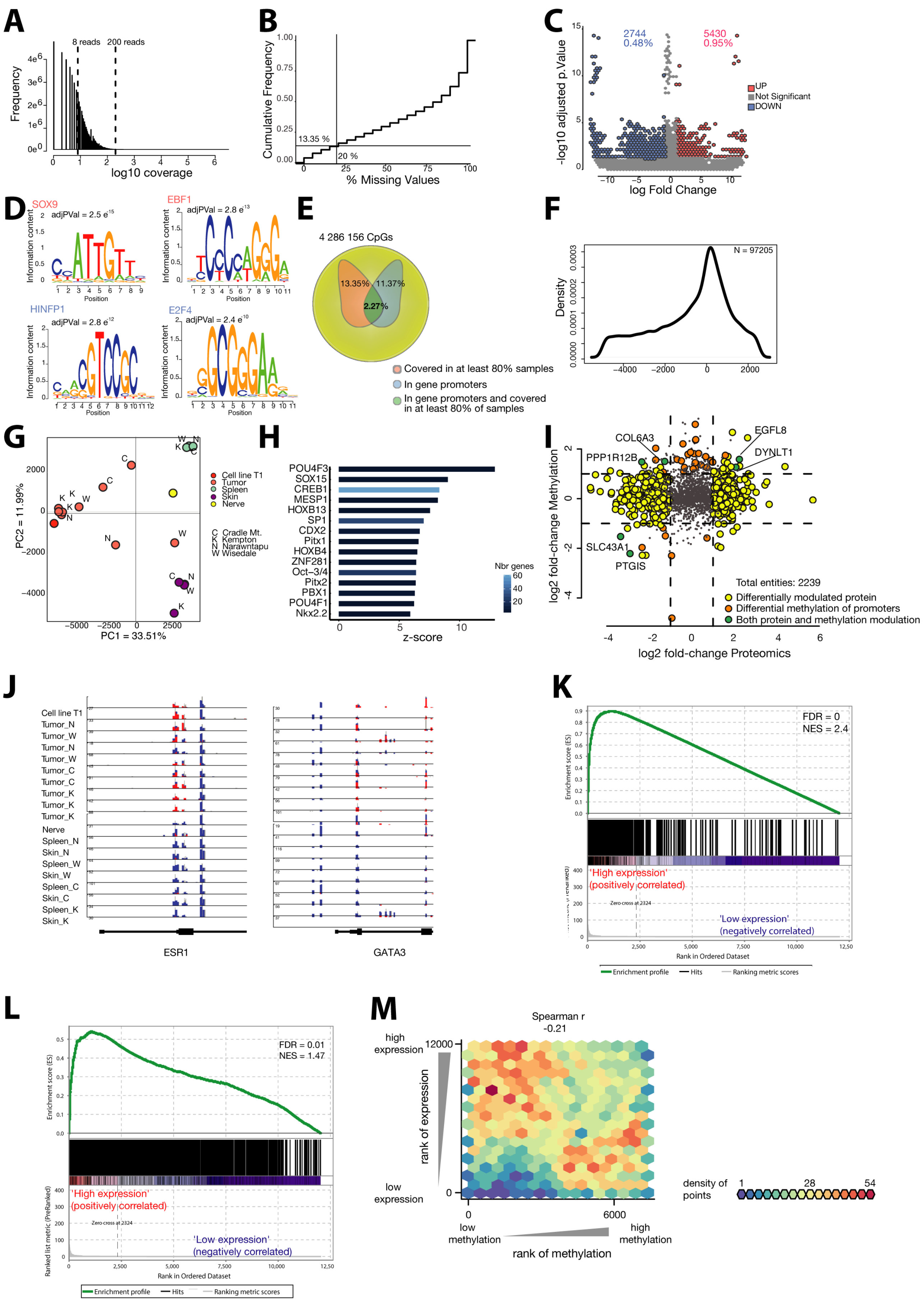

A
8 reads
200 reads
Frequency
log10 coverage
B
Cumulative Frequency
13.35 %
20 %
% Missing Values
C
2744
0.48%
5430
0.95%
UP
Not Significant
DOWN
-log10 adjusted p.Value
log Fold Change
D
SOX9
adjPVal = 2.5 e15
EBF1
adjPVal = 2.8 e13
HINFP1
adjPVal = 2.8 e12
E2F4
adjPVal = 2.4 e10
Information content
Position
E
4 286 156 CpGs
13.35%
11.37%
2.27%
Covered in at least 80% samples
In gene promoters
In gene promoters and covered in at least 80% of samples
F
N = 97205
Density
G
Cell line T1
Tumor
Spleen
Skin
Nerve
C Cradle Mt.
K Kempton
N Narawntapu
W Wisedale
PC2 = 11.99%
PC1 = 33.51%
H
POU4F3
SOX15
CREB1
MESP1
HOXB13
SP1
CDX2
Pitx1
HOXB4
ZNF281
Oct-3/4
Pitx2
PBX1
POU4F1
Nkx2.2
Nbr genes
z-score
I
COL6A3
PPP1R12B
EGFL8
DYNLT1
SLC43A1
PTGIS
Total entities: 2239
Differentially modulated protein
Differential methylation of promoters
Both protein and methylation modulation
log2 fold-change Methylation
log2 fold-change Proteomics
J
Cell line T1
Tumor_N
Tumor_W
Tumor_N
Tumor_W
Tumor_C
Tumor_C
Tumor_K
Tumor_K
Tumor_K
Nerve
Spleen_N
Skin_N
Spleen_W
Skin_W
Spleen_C
Skin_C
Spleen_K
Skin_K
ESR1
GATA3
K
FDR = 0
NES = 2.4
Enrichment score (ES)
'High expression' (positively correlated)
Zero cross at 2324
'Low expression' (negatively correlated)
Rank in Ordered Dataset
Enrichment profile
Hits
Ranking metric scores
L
FDR = 0.01
NES = 1.47
Enrichment score (ES)
Ranked list metric (PreRanked)
'High expression' (positively correlated)
Zero cross at 2324
'Low expression' (negatively correlated)
Rank in Ordered Dataset
Enrichment profile
Hits
Ranking metric scores
M
Spearman r
-0.21
high expression
low expression
rank of expression
low methylation
high methylation
rank of methylation
density of points

Figure S3, Related to Figure 2: Global DNA methylation analysis and correlations

(**A**) Coverage histogram across the individual CpGs (4,286,156). CpGs covered by at least eight and at most 200 reads are considered for further analysis. Coverage lower than eight reads was deemed insufficient for computing accurate DNA methylation levels. Furthermore, sites covered by more than 200 reads might harbor unreliable measurements introduced by excessive amplification or repetitive sequences. (**B**) Cumulative frequency distribution of the percentage of not detected CpGs across the samples. 572018 (13.35%) of CpGs are detected in at least 80% of samples (15 out of 19). (**C**) Healthy versus Tumor volcano plot of individual detected CpGs in more than 80% of the samples (572018 CpGs). The differential analysis of healthy versus tumor samples (cell line excluded) revealed a total of 2744 tumor hypermethylated and 5430 tumor hypomethylated CpGs (absolute log Fold-change ≥1, adjusted p value ≤ 0.05). (**D**) AME motif enrichment in fragments hypomethylated or hypermethylated in tumor samples compared to healthy tissues, red and blue respectively. The top two enriched motifs (sorted according to the adjusted p value of enrichment) are shown for each class of CpGs. (**E**) Characterizing individual CpGs: detected, annotated. 97 205 individual CpGs are detected in at least 80% of the samples and localized in the promoters of annotated genes (from -5 kb to 2.5 kb around the TSS). (**F**) Density distribution of the CpG distance to the Transcription Start Site (TSS) of annotated genes. (**G**) Principal component analysis on the promoter averaged CpGs across the 19 samples. Samples separate mainly on the tumor versus healthy profiles (PC1 – 33.5% of the variability), with spleen versus skin differences driving the PC2 (11.99% of the variability). Sampling locations are indicated in capital letters (C Cradle Mountain, K Kempton, N Narawntapu, W Wisedale). (**H**) Top 15 enriched transcription factors (TF) for the differentially methylated gene promoters: healthy versus tumor samples. The enrichment was performed with MetaCore and the reported TF have all p values <= 0.05 and are ordered according to their z-score of enrichment. (**I**) Comparison of the proteomics and methylation hits. Log fold-change of tumor versus healthy analysis was compared to the log fold-change of promoter averaged CpGs healthy versus tumor. 2239 entities were detected in both analyses. Yellow, orange and green circles stand for

entities found as differentially modulated only in proteomics, only in methylation or in both, respectively. (**J**) Integrative Genomics Viewer plots of promoter CpG methylation across samples for *ESR1* and *GATA3*. Red stands for methylated, while blue stands for un-methylated. (**K** and **L**) Gene set enrichment analysis pre-ranked by the RNA-seq expression of the top 15% most abundant proteins (817) (**K**) and the top 15% least methylated (936) gene promoters (**L**) of DFTD cell line (T1). (**M**) Comparison of ranked gene expression and ranked gene promoter methylation of the DFTD cell line (T1) (6974 genes). Spearman correlation is reported.

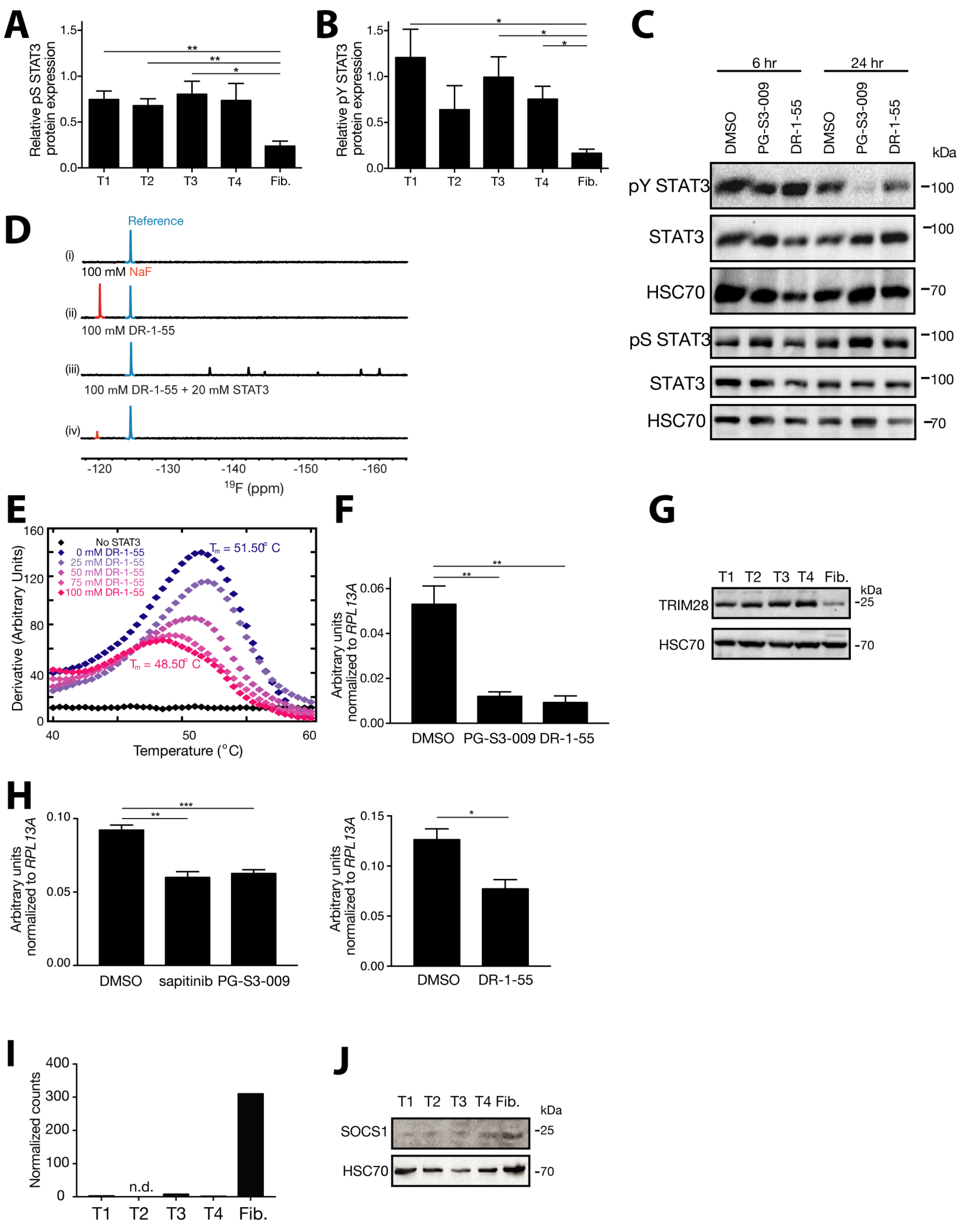
A
Relative pS STAT3 protein expression
1.5
1.0
0.5
0.0
T1 T2 T3 T4 Fib.
B
Relative pY STAT3 protein expression
T1 T2 T3 T4 Fib.
C
6 hr
24 hr
DMSO PG-S3-009 DR-1-55 DMSO PG-S3-009 DR-1-55
kDa
pY STAT3
STAT3
HSC70
pS STAT3
STAT3
HSC70
100
70
D
Reference
(i)
100 mM NaF
(ii)
100 mM DR-1-55
(iii)
100 mM DR-1-55 + 20 mM STAT3
(iv)
-120 -130 -140 -150 -160
19F (ppm)
E
No STAT3
0 mM DR-1-55
25 mM DR-1-55
50 mM DR-1-55
75 mM DR-1-55
100 mM DR-1-55
Tm = 51.50° C
Tm = 48.50° C
Derivative (Arbitrary Units)
Temperature (°C)
F
Arbitrary units normalized to RPL13A
DMSO PG-S3-009 DR-1-55
G
T1 T2 T3 T4 Fib.
kDa
TRIM28
HSC70
-25
-70
H
Arbitrary units normalized to RPL13A
DMSO sapitinib PG-S3-009
DMSO DR-1-55
I
Normalized counts
n.d.
T1 T2 T3 T4 Fib.
J
T1 T2 T3 T4 Fib.
kDa
SOCS1
HSC70
-25
-70

Figure S4, Related to Figure 3: Analyses of STAT3, TRIM28, SOCS1 and characterization of STAT3 inhibitor DR-1-55

(**A** and **B**) Quantification of pS-STAT3 (**A**) and pY-STAT3 (**B**) normalized to HSC70 and total STAT3 from Western blots of total STAT3, pS-STAT3, pY-STAT3 shown in **Figure 3A**. Statistical significance was calculated by unpaired t-test. Bars represent mean +/- SEM. (**C**) Western blots of total STAT3, pS-STAT3 and pY-STAT3 upon treatment with the STAT3 inhibitors PG-S3-009 (2 μM) and DR-1-55 (4 μM). (**D**) ^{19}F NMR spectra of (i) 100 μM reference (5-fluorotryptophan). (ii) 100 μM sodium fluoride with reference. (iii) 100 μM DR-1-55 with reference. (iv) 100 μM DR-1-55 and with 20 μM STAT3 protein and the reference. All samples were prepared in PBS buffer with 2% glycerol, 10% DMSO and 10% D_2O. (**E**) Thermal shift assays of 2 μM STAT3 protein with varying concentrations of DR-1-55. STAT3 was incubated with varying concentrations of DR-1-55 for 10 min and Sypro orange (at a final concentration of 5x) was introduced to the sample well. The temperature was increased in 0.5 °C increments from 20 – 80 °C and the fluorescence intensity was recorded at each step. The first derivative plot of the melting curve was determined and the denaturation temperature (local maxima) was calculated from the average of three independent measurements and a representative denaturation curve is shown. (**F**) *MMP2* was measured by real-time PCR from DFTD cells treated with PG-S3-009, DR-1-55 or DMSO as control for 24 hours (n=3 replicates). One out of two similar experiments shown. Statistical significance was calculated by One-way ANOVA. Bars represent mean +/- SEM. (**G**) Western blot for TRIM28 from DFTD cell lines and Tasmanian devil fibroblasts. (**H**) *TRIM28* was measured by real-time PCR from DFTD cells treated with sapitinib, PG-S3-009, DR-1-55 or DMSO as control for 24 hours (n=3 replicates). One out of two similar experiments shown. Statistical significance was calculated by One-way ANOVA. Bars represent mean +/- SEM. (**I**) RNAseq-derived expression values of *SOCS1* from DFTD cell lines and Tasmanian devil fibroblasts. (**J**) Western blot for SOCS1 from DFTD cell lines and Tasmanian devil fibroblasts.

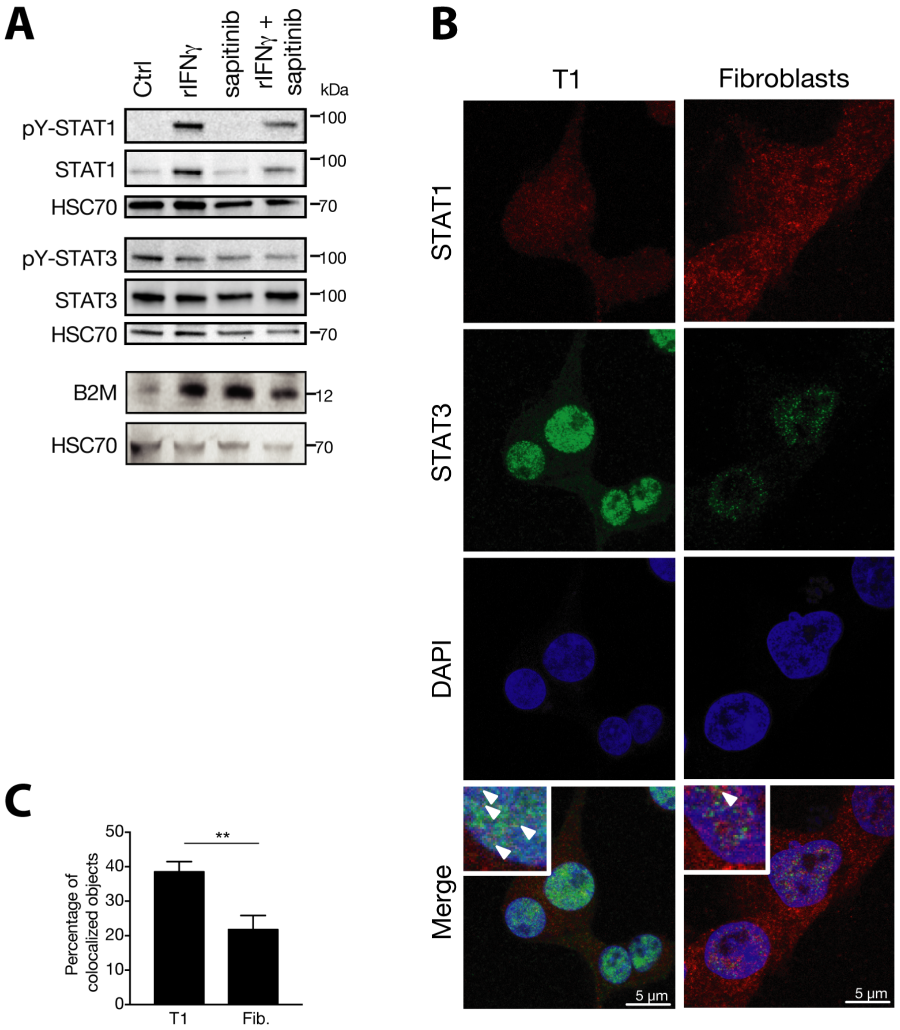

Figure S5, Related to Figure 4: Effects of recombinant interferon gamma, sapitinib and STAT3-STAT1 co-localization

(**A**) Western blots for pY-STAT1, STAT1, pY-STAT3, STAT3 and B2M from DFTD cell line (T1) upon treatment with rIFNγ, sapitinib or rIFNγ and sapitinib for 6 hours. (**B**) Representative images of immunofluorescent staining for STAT1 and STAT3 co-localization in T1 and Fib. White arrows point toward co-localization events. (**C**) Quantification of STAT3-STAT1 signal. Statistical significance was calculated by unpaired t-test. Bars represent mean +/- SEM.

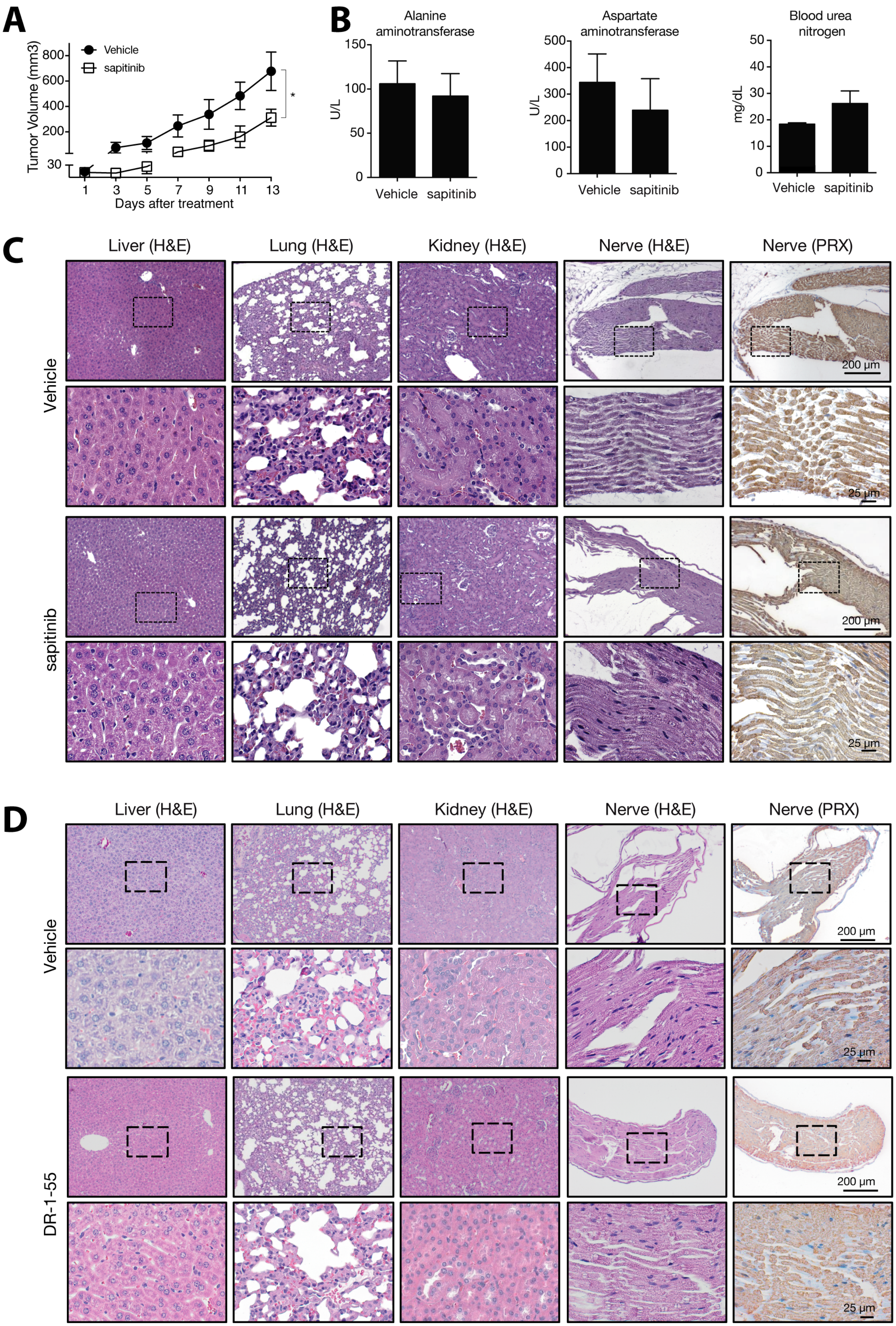

A
Tumor Volume (mm3)
800
600
400
200
30
Vehicle
sapitinib
*
1
3
5
7
9
11
13
Days after treatment
B
Alanine aminotransferase
U/L
150
100
50
0
Vehicle
sapitinib
Aspartate aminotransferase
U/L
500
400
300
200
100
0
Vehicle
sapitinib
Blood urea nitrogen
mg/dL
50
40
30
20
10
0
Vehicle
sapitinib
C
Liver (H&E)
Lung (H&E)
Kidney (H&E)
Nerve (H&E)
Nerve (PRX)
Vehicle
sapitinib
200 µm
25 µm
200 µm
25 µm
D
Liver (H&E)
Lung (H&E)
Kidney (H&E)
Nerve (H&E)
Nerve (PRX)
Vehicle
DR-1-55
200 µm
25 µm
200 µm
25 µm

Figure S6, Related to Figures 4 and 5: Pathologic examination of xenograft mouse models

(**A**) Tumor volume of NSG mice transplanted with DFTD tumor cells 27 days prior to treatment with either vehicle or 50 mg/kg sapitinib daily (bilateral tumors, n = 5 mice per group). Symbols represent mean +/- SEM. (**B**) Serum concentration of alanine aminotransferase, aspartate aminotransferase and blood urea nitrogen from mice treated with either vehicle or 50 mg/kg sapitinib daily for 13 days. Bars represent the mean of 3 to 5 mice +/- SEM. (**C**) H&E stainings for liver, lung, kidney and nerve tissue of mice treated with either vehicle or 50 mg/kg sapitinib daily for 13 days. Nerve tissue was also immunohistochemically stained for Periaxin (PRX). Scale bars, 200 μm and 25 μm. (**D**) H&E stainings for liver, lung, kidney and nerve tissue of mice treated with either vehicle or 10 mg/kg DR-1-55 each second day. Nerve tissue was also immunohistochemically stained for Periaxin (PRX). Scale bars, 200 μm and 25 μm. Statistical significance was calculated by (**A**) Two-way ANOVA with Bonferroni correction or (**B**) unpaired t-test.
